# Nuclear Transglutaminase 2 interacts with topoisomerase II⍺ to promote DNA damage repair in lung cancer cells

**DOI:** 10.1186/s13046-021-02009-2

**Published:** 2021-07-05

**Authors:** Xiao Lei, Kun Cao, Yuanyuan Chen, Hui Shen, Zhe Liu, Hongran Qin, Jianming Cai, Fu Gao, Yanyong Yang

**Affiliations:** 1 Department of Radiation Medicine, Faculty of Naval Medicine, Naval Medical University, 800, Xiangyin Road, 200433 Shanghai, P.R. China; 2 Department of Radiation Oncology, The First Medical Center of PLA General Hospital, Beijing, P.R. China; 3 Department of Nuclear Radiation, Shanghai Pulmonary Hospital, Tongji University, 507, Zhengmin Road, 200433 Shanghai, P.R. China; 4 School of Public Health and Management, Wenzhou Medical University, University Town, Wenzhou, Zhejiang P.R. China

**Keywords:** DNA damage repair, Transglutaminase 2 (TG2), DNA double strand breaks (DSBs), Topoisomerase IIα (TOPOIIα), Cancer therapy

## Abstract

**Background:**

To block repairs of DNA damages, especially the DNA double strand break (DSB) repair, can be used to induce cancer cell death. DSB repair depends on a sequential activation of DNA repair factors that may be potentially targeted for clinical cancer therapy. Up to now, many protein components of DSB repair complex remain unclear or poorly characterized. In this study, we discovered that Transglutaminase 2 (TG2) acted as a new component of DSB repair complex.

**Methods:**

A bioinformatic analysis was performed to identify DNA damage relative genes from dataset from The Cancer Genome Atlas. Immunofluorescence and confocal microscopy were used to monitor the protein localization and recruitment kinetics. Furthermore, immunoprecipitation and mass spectrometry analysis were performed to determine protein interaction of both full-length and fragments or mutants in distinct domain. In situ lung cancer model was used to study the effects cancer therapy in vivo.

**Results:**

After DSB induction, cytoplasmic TG2 was extensively mobilized and translocated into nucleus after phosphorylated at T162 site by DNA-PKcs. Nuclear TG2 quickly accumulated at DSB sites and directly interacting with Topoisomerase IIα (TOPOIIα) with its TGase domain to promote DSB repair. TG2 deficient cells lost capacity of DSB repair and become susceptible to ionizing radiation. Specific inhibition of TG2-TOPOIIα interaction by glucosamine also significantly inhibited DSB repair, which increased sensitivity in lung cancer cells and engrafted lung cancers.

**Conclusions:**

These findings elucidate new mechanism of TG2 in DSB repair trough directly interacting with TOPOIIα, inhibition of which provided potential target for overcoming cancer resistance.

**Supplementary Information:**

The online version contains supplementary material available at 10.1186/s13046-021-02009-2.

## Background

Integrity of genome DNA is continuously maintained in eukaryotic cells throughout their whole lifecycles. Mostly, the damaged DNA can be repaired under a comprehensive machinery protecting them from endogenous and exogenous genotoxic stresses[1, 2]. Deficiencies in functions of DNA damage repair result in accumulation of genetic mutations, leading to cell senescence, carcinogenesis or cell death [3, 4]. On the other hand, cancer cells abnormally carry stronger capacity for DNA damage repair, making them more resistant against various anti-cancer therapies[5, 6]. Therefore, to completely elucidate the mechanisms of DNA damage repair is helpful of improving our realizations on the precise cellular response to multiple DNA damages, which can be applied to overcome the relative resistances of cancer cells during various clinical cancer therapies[7].

DNA double strand break (DSB) is one of the most severe types of DNA damage[8]. If un-repaired, DSB resulted in chromosome aberration, genome instability, carcinogenesis or cell death[3, 9]. When DSB occurs, protein factors accumulate at sites of DSB to compose an activating DNA repair complex[10]. However, many members of these factors are either still unknown or poorly characterized, which becomes a difficulty for intentionally controlling the process of DSB repair during clinical applications.

In an early stage of our current study, transglutaminase 2 (TG2) was firstly discovered as a new factor to be recruited to DSB sites. Although TG2 was previously suggested for its possible existence in the processes of DNA damages [11, 12], its functions were not realized, including whether TG2 directly took part in the processes of DNA damage repair and what kind of function it actually performed. On the other hand, some earlier studies also revealed that TG2 performed diverse roles during cell apoptosis, autophagy, as well as epithelial-mesenchymal transition through its crosslink or GTP binding activities[13–16], which showed little relevance to DNA damage repair. Moreover, under normal conditions, it is worth to note that most of TG2 (93 %) resides in cytoplasm while almost all of DNA damage processes occur inside nucleus[17]. Therefore, it was especially hard to explain how to let cytoplasmic TG2 actively move to DSB sites in nucleus and play a role during DNA damage repair.

Through our continuing investigation on TG2 after DNA damage, we successfully discovered that TG2 was phosphorylated at T162 site by DNA-PKcs and translocated from cytoplasm into nucleus immediately after occurrence of DSB. Inside nucleus, TG2 was further recruited to DSB sites and bound to topoisomerase IIα (TOPOIIα) through its TGase domain. Remarkably, TG2 deficiencies in cancer cells resulted in the defective function of DSB repair, which increased cancer cells death during chemotherapy or radiotherapy. Therefore, a novel realization was obtained on TG2’s roles of DNA damage repair in our present study. Furthermore, inhibiting TG2-TOPOIIα interaction could become a novel and potential strategy to overcome cancer cell resistance to clinical therapies.

## Methods

### Bioinformatic data mining

Bioinformatic analysis was performed by using the data from radiotherapy treated patients of lung cancer, colorectal cancer, head and neck carcinoma, which were obtained from The Cancer Genome Atlas (TCGA). Patients resistant to radiotherapy were defined as those who had residual lesions after therapy and experienced local recurrence of death within 3 years. Patients sensitive to radiotherapy were defined as those who achieved complete remission after therapies or survived more than 3 years. Differential expressed genes were identified from these two groups including the radiotherapy sensitive group and radiotherapy resistant group. Overall survival and progression-free survival were estimated using Limma software and univariate analysis were performed using Cox proportional hazard model.

### Cells and treatment

Lung cancer cell lines (A549, H460, H1299, H1975, H358), human bronchial epithelial cell line BEAS-2B, and mouse lewis lung cancer cells (LLC) were all purchased from the ATCC (USA). YFP-53BP1-HT1080 cells were a kind gift from Department of Radiation Oncology, UT Southwestern Medical Center. M059K and M059J were purchased from KeyGENE BioTech Co., China. A549, H460, H1975, H358 and HT1080 were maintained in DMEM with 10 % fetal bovine serum at 37℃ in a 5 % CO_2_ humidified chamber. BEAS-2B, H1299 and LLC cells was maintained in RMPI 1640 medium with the same supplement. Cells were treated with ionizing radiation, Etoposide, CPT, H_2_O_2_, 4NQO or UV to induce different types of DNA damages. Specific inhibitors for ATM, ATR and DNA-PKcs, known as KU55933, VE821 and NU7441, were purchased from Sellecks Biotech. For laser irradiation, a 365-nm pulsed nitrogen laser was directly coupled to the fluorescence path of the microscope (Axiovert 200 M; Carl Zeiss).

### Plasmids and transfection

TGM2 shRNA plasmids, CRISPR Cas9 knockout plasmid, TGM2 overexpressing plasmid and TOPOIIα overexpressing plasmid were constructed by Biolink BioTECH. (Shanghai, China). Plasmids encoding TGM2 fragments (AB, ABC, AB + C1, BC, CD) and different mutants for loss of functions (C277S, W241A, R580A, Y516F), were cloned in pLenO-GTP (Table S3; Fig. S5). TG2 S68A mutant, T162A mutant and S68A + T162A mutant expressing plasmids were constructed by Zorin BioTECH (Shanghai, China). For stable clones, cells were infected with lentivirus and selected with puromycin, after which single clone was picked out. Then cells were subjected to further experiment.

### Clonogenic survival

Clonogenic survival was used to assess cell sensitivity to DNA damage treatments. Cells were calculated and seeded in the 6-well plates, and subjected to DNA damage treatments including ionizing radiation, etoposide, CPT. After incubated for 10–14 days, cells were fixed with paraformaldehyde and stain with crystal violet. The survival fractions were analyzed using a more target and one-hit model.

### Apoptosis assay

At 24 h after irradiation, cell apoptosis was detected by using an Annexin V-FITC and PI apoptosis detection kit according to the manufacturer’s instructions (Yeasen, Shanghai, China). After Annexin V and PI staining, cells apoptosis was analyzed with an Cytoflex flow cytometry (Beckman, USA).

#### Neutral Comet Assay

DSB were determined by ameliorating the a forementioned neutral comet assay. Firstly, slides were immersed in a 1 % NMA and dry thoroughly. Next, the single cell suspension prepared (2 × 10^4^ cells / ml), was immersed in LMA under 40℃ water bath. Thirdly, cell suspension was mixed and rapidly pipetted onto the surface of the precoated slide. The slides were then incubated at 4℃ for 25 min at 25 V in TBE. Then the gel was stained with PI (10 µg/ml) for 20 min and then rinsed gently with ddH_2_O. Finally, all gels were examined by Olympus BX60 fluorescence microscope. Total 100 images in each slide were analyzed using CASP 1.2.3b2 software (CASPlab, Poland).

### Western blotting and immunoprecipitation

Total proteins were obtained using ProteoJET Mammalian Cell Lysis Reagent. ProteinExt® Mammalian Nuclear and Cytoplasmic Protein Extraction Kit was used for extracting nuclear and cytoplasmic protein, according to the manufacturer’s instructions. Then the samples were analyzed by western blotting with chemiluminescent detection. IP was performed by using a Pierce™ Co-Immunoprecipitation Kit according to the manufacturer’s instructions. All the primary antibodies were listed in Table S2.

### Label-free quantitative proteomics of TG2 immunoprecipitated protein complex

In untreated or irradiated A549 cells, protein complex was immunoprecipitated with TG2 specific antibody as described above. After then, protein samples were analyzed with a Label-free LC-MS analysis as previous described [18]. Briefly, after quantification, protein samples were digested by using a Filter-Aided Sample Preparation (FASP) method. Then the acidified tryptic peptides were separated on an Easy nLC High performance liquid chromatography system (Thermo Scientific EASY column). Finally, the samples were analyzed with an orbitrap Q Exactive mass spectrometry (Thermo/Finnigan).

### Chromatin fraction

Chromatin fraction was performed to isolate the chromatin binding protein as well as soluble proteins, as previous described [19]. Briefly, A549 cells with and without irradiation were firstly incubated on ice for 10 min with protein extraction buffer supplemented with protease inhibitor cocktail (Selleck Biotech.) and 0.5 % Triton X-100. Then cells were centrifuged at 1300 g for 5 min to get supernatant 1 (S1), which was further centrifuged to get S2 at 20,000 g for 5 min. Nuclei (P1) were treated with micrococcal nuclease at 37℃ for 30 min and then on ice for 30 min. P2 and S3 was isolated through low speed centrifuge. P2 was then incubated with a EDTA containing buffer and centrifuged to get P3. Then the protein S3 and P3 were subjected to Western blot analysis.

### Immunofluorescence analysis

We used an immunofluorescence assay to detect γH2AX foci, the subcellular location of 53BP1, TG2 and TOPOIIα. Briefly, cells were seeded on 22 × 22mm^2^ cover glasses. After different treatment, cells were fixed in 4 % paraformaldehyde and permeabilized in 0.5 % Triton X-100. After blockage, cells were stained with primary antibody and then with the secondary antibody. Cellular images were obtained using a confocal microscope (Nikon). Image Pro Plus (Media Cybernetics) were used to count the γH2AX foci per cell, and at least 100 cells per group were counted. The colocalization of proteins was also analyzed with the Image Pro Plus software.

### Animals and glucosamine treatments

The whole protocols were approved by the Ethics Committee of Naval Medical University, China. Female C57BL/6 mice, 8 weeks old, obtained from the Experimental Animal Center of Chinese Academy of Sciences. Mice were implanted with LLC cells (25ul, 2 × 10^5^cells) in fixed location which was 5mm distance from the lower end of the xiphoid process. Glucosamine (150 mg/kg/d) was delivered to the corresponding groups by intraperitoneal injection 3 days before expose to whole lung irradiation at a single dose of 15 Gy.

### Histopathology and immunohistochemistry

On 7th and 20th days after lung irradiation, tumor tissues were isolated, fixed and subjected to sectioning. Tissues were stained with H&E and antibodies for Ki67 and TUNEL. Two pathologists independently examined 30 fields per group.

### Statistical analysis

Data were expressed at the means ± standard error of mean. GraphPad Prism 8 software was applied for statistical analysis. For comparison among multiple groups, one-way ANOVA was performed. Student’s t test was used to compare between two groups. All experiments were performed at least 3 independent times.

## Results

### Activities of TG2 dynamically correlated with various activities of DNA damage repair

**Fig. 1:**
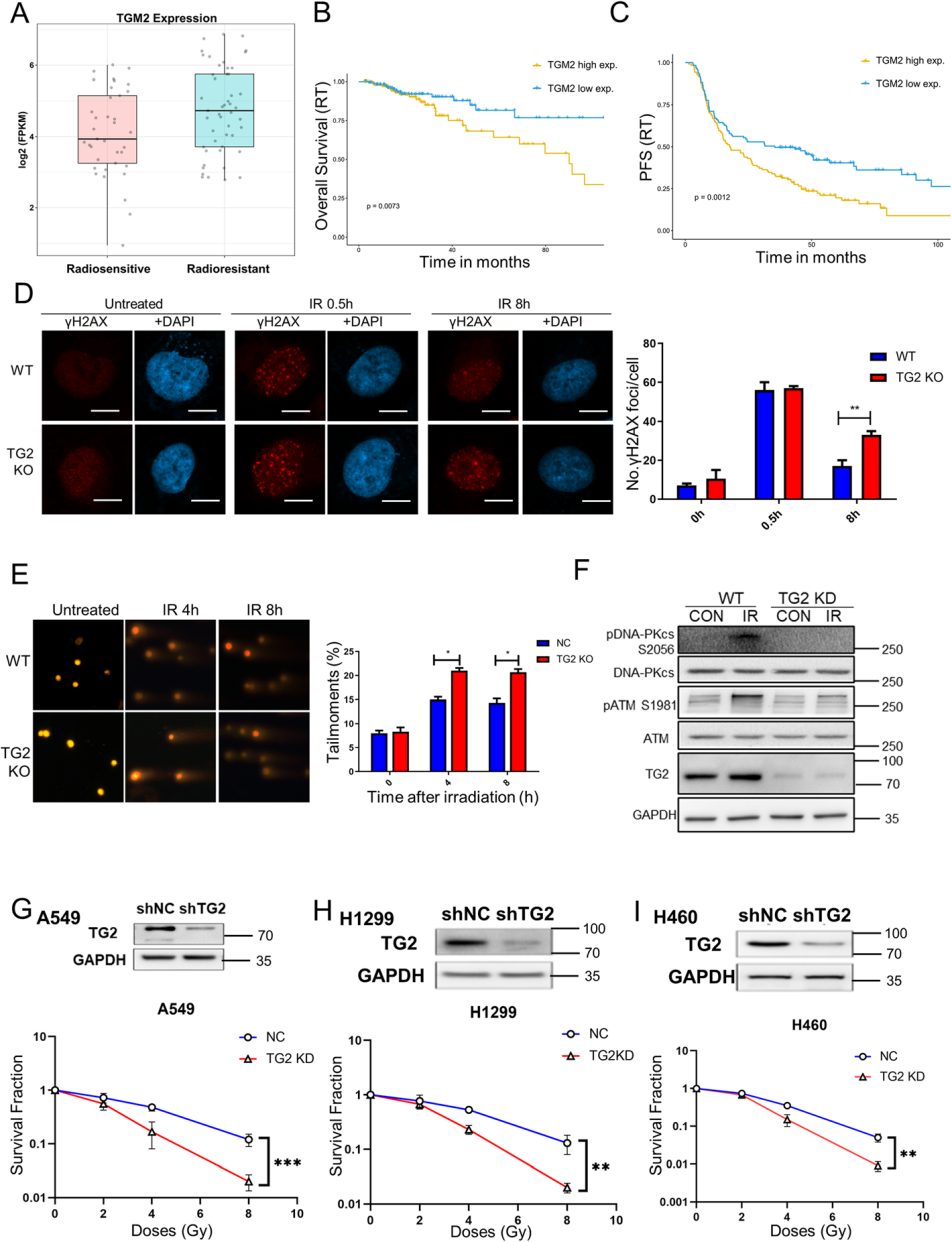
TG2 was necessary for effective DNA damage repair. **A**: mRNA expressions of TGM2 (TG2’s gene name) gene in both radiosensitive (*n* = 38) and radioresistant (*n* = 49) cancer patients, which were identified by using an integrative TCGA analysis on various cancers including lung cancer, colorectal cancer, head and neck carcinoma. **B**, **C**: The correlations of TGM2 gene mRNA expression with both overall survival (OS) (*P* = 0.0073) and progression-free survival (PFS) (*P* = 0.0012) of radiotherapy treated cancer patients. **D**: Both original A549 cells and the A549 cells of TG2 KO were exposed to 8 Gy γ-irradiation. Afterward, cells were immune-stained for recognizing γ-H2AX with proper anti-γ-H2AX antibody and merged with DAPI stained result at 0, 0.5 and 8 h after irradiation. Scale bar, 20 μm. Panel D showed the representative images with average numbers of γ-H2AX foci per cell after quantificational analyses. **E**: Representative images of neutral comet assay showed the tail moment in both original A549 cells and the A549 cells of TG2 KO for evaluating their DNA damage (8 Gy). Scale bar, 50 μm. Cells from different groups were quantificationally analyzed for their tail moments in comet assay. **F**: Western blotting analysis activation of DNA-PKcs and ATM in TG2 KD A549 cells after 8 Gy irradiation. **G**-**I**: Colonogenic assay was used to detect colony formation of TG2 knockdown A549, H1299 and H460 cells after 0, 2, 4 or 8 Gy irradiation. *P < 0.05. **P < 0.01

DNA damage repair acted in many types of tumor cells to mainly resist to both radiotherapy and chemotherapy[5]. In order to identify the unknown resistant genes relative to the DNA damage response (DDR), a screening of bioinformatics data mining was performed by us through analyzing TCGA datasets of radiotherapy treated patients, covering head and neck carcinoma, lung cancer as well as colorectal cancer. Total of 480 genes had significantly upregulated expressions (> 1.5 fold) in patients who were known to have resistances to radiotherapy, when compared with those sensitive to treatments (Fig. S1A). Among these genes, TGM2 (gene name of TG2 protein) was especially recognized for its significantly elevated expression levels in those patients who are resistant to DNA damages (Fig. 1 A). Remarkably, TGM2 expression was also negatively correlated to both OS and PFS in the radiotherapy treated cancer patients (Fig. 1B, C), as well as the overall survival of all patients (Fig. S1B, C). We collected 80 pairs of clinical lung cancer tissues, and found that high expression of TG2 is related to patient survival of the patients (Figure S1D-F; Table S7). After this finding, the expression of TG2 protein were broadly analyzed in several types of cancer cell lines. Remarkably, expression of TG2 was found to have high expression in lung cancer cells (Fig. S2A), which were selected in this study.

To pursuit whether TG2 play essential role in DNA damage and cancer resistance, TG2 expression was either knockdown with shRNA or knockout with CRISPR Cas9 system in several lung cancer cells, including A549, H1299, H460 cells (Fig. 1G-I, S2C, E; Table S1). Afterward, these TG2 deficient cells were subjected to different DNA damage treatments, including ionizing radiation (IR), Etoposide and Camptothecin (CPT). Our data showed that the number of γH2AX foci were also significantly higher in irradiated TG2 deficient A549 cells than that in TG2 proficient cells (Fig. 1D). Furthermore, at 4 and 8 h after 8 Gy γ-irradiation, the contents of tail moment representing the overall damaged DNA were significantly higher in TG2 deficient A549 cells than those in control A549 cells (Fig. 1E). DNA damage initiates particular repair signaling pathways, which were mainly orchestrated by the upstream factors of DNA-PKcs and ATM[4, 10]. Here, in TG2 deficient cells, radiation-induced phosphorylation levels of both DNA-PKcs (pS2056) and ATM (pS1981) were both attenuated (Fig. 1 F), indicating that the early activations of DNA damage repair were disabled. To characterize the influence of TG2 on cellular sensitivity to DNA treatments, colony-formation efficiency and cell apoptosis was detected in both TG2 proficient and TG2 deficient cells. In clonogenic assay, TG2 deficiency resulted in less colony formation after radiation in A549, H1299 and H460 cells, when compared with each normal cell lines, respectively (Fig. 1G, H, I). CRISPR Cas9-derived TG2 knockout A549 cells also became more sensitive to radiation induced cell killing (Fig. S2C). Overexpression of TG2 in H1299 cells with low TG2 expression significantly increased cell survival after irradiation (Fig. S2D). More apoptotic cells were also observed in TG2 KD A549 cells than that in normal A549 cells after irradiation (Fig. S2E). TG2 deficiency also resulted in reduced colony-formation efficiency in Etoposide treated cells (Fig. S2F), while no significant difference was observed in CPT treated cells (Fig. S2G). These data clarified that TG2 was indispensable for successful DNA damage repair, and suggested importance to further realize details of its critical roles.

### TG2 was phosphorylated and translocated from cytoplasm into nucleus in a manner of DNA-PKcs dependence after DSB induction

**Fig. 2:**
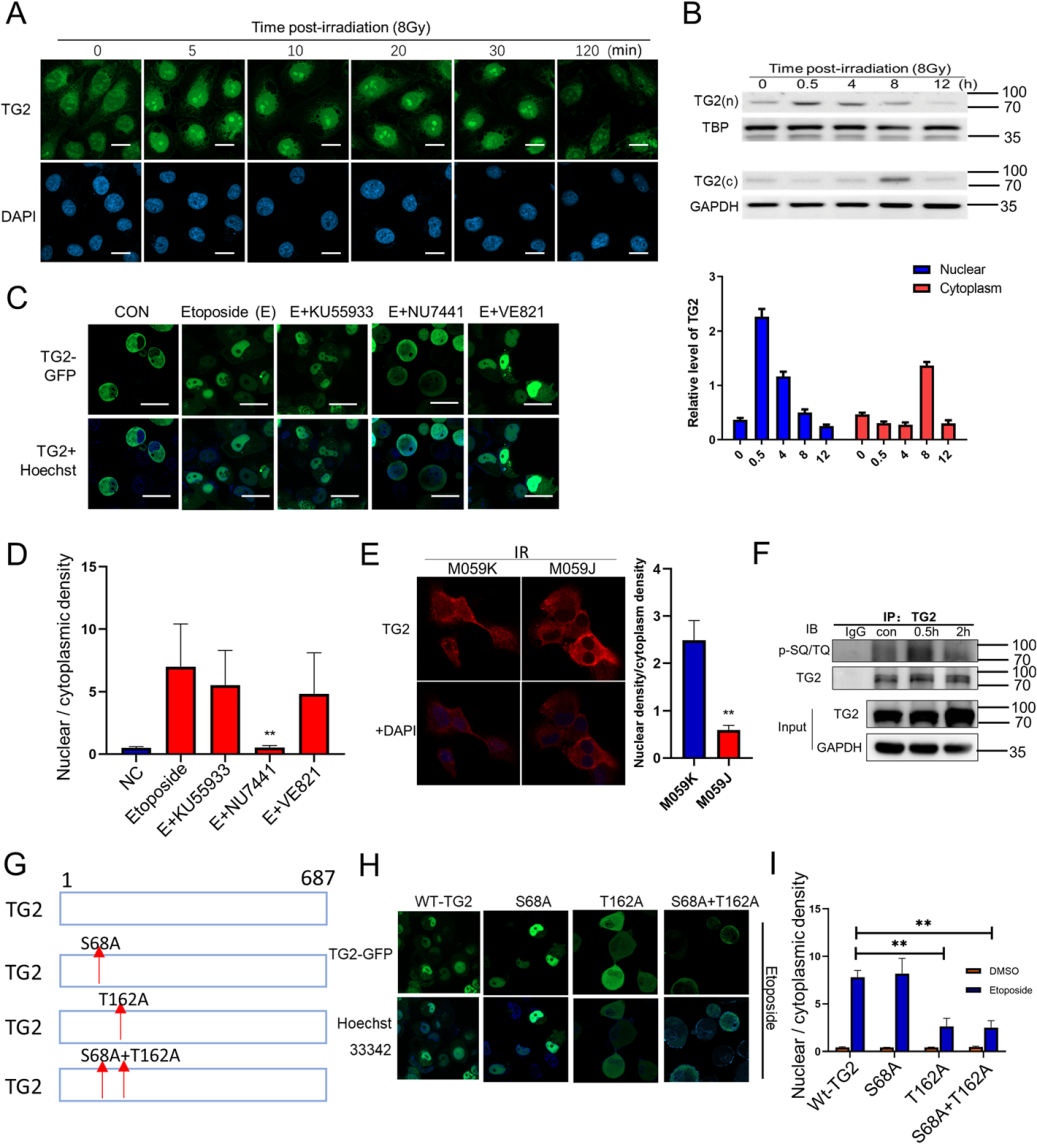
Occurrence of DNA-PKcs dependent nuclear translocation of TG2 in response to DNA double strand breaks. **A**: Representative images for immunofluorescence staining of TG2 (green) in A549 cells at different times after exposure to 8 Gy γ-irradiation. Scale bar, 20 μm. **B**: Immunoblot of TG2 in both nucleus and cytoplasm lysates isolated from irradiated A549 cells. The levels of TG2 protein expressions in both Nuclear and cytoplasm were quantified with image J software. **C**: Localizations of TG2 in the same cells were determined in response to the DSB treatments of KU55933 (10µM), NU7441 (10µM) or VE821 (10µM). Scale bar, 20 μm. **D**: The ratio of nuclear TG2 and cytoplasm TG2 was quantified and analyzed. **E**: Representative images for immunofluorescence staining of TG2 in both DNA-PKcs proficient cells (M059K cells) and deficient cells (M059J cells) at 0.5 h after 8 Gy irradiation. **F**: Immunoprecipitations of TG2 were performed with TG2 antibody for the irradiated A549 cells, which was followed by the performed detection of p-SQ/TQ motif on TG2. **G**: The design of DNA-PKcs relative TG2 phosphorylation site mutant. **H**, **I**: wt-TG2-GFP, S68A-GFP, T162A-GFP and S68A + T162A-GFP expressing cells were treated with Etoposide (100 µg/ml, 0.5 h) and images were taken. Scale bar, 50 μm. Hoechst 33,343 was used as an indicator of nucleus. The ratio of nuclear TG2 and cytoplasm TG2 was quantified and analyzed. **P < 0.01

In order to pursuit how TG2 respond to DNA damage and confer cancer resistance, mobilization of TG2 was detected in lung cancer cells after treated with various kinds of DNA damaging reagents. Firstly, expression and localization of TG2 were detected through WB and Immunofluorescence staining after DNA damage (Fig. 2 A, S2B). Remarkably, most of TG2 was observed to translocate from cytoplasm to nucleus in A549 cells as early as 30 min after γ-ray-induced DSB (Fig. 2 A). The immediate nuclear translocation after DSB induction was further confirmed on the relative detections of nuclear protein and cytoplasm protein (Fig. 2B). Then we determined whether activation of TG2 responded to comprehensive DNA damages, A549 cells were treated with various types of genotoxic agents including: the group of Etoposide and H_2_O_2_ mainly for inducing DSB; as well as the other group including CPT, 4-Nitroquinoline N-oxide (4NQO) and Ultraviolet radiation (UV) for inducing DNA single strand break (SSB) or DNA crosslink. Interestingly, TG2 was also found to translocate into nucleus at 30 min in response to DSB inducible agents of both Etoposide and H_2_O_2_, however, minor level of nuclear translocation could be observed in the CPT, 4NQO or UV treated cells (Fig. S3A, B). Together, these data revealed that TG2 had specific response to DSB through a quick mobilization from cytoplasm into nucleus.

It was previously known that DSB triggered the activations and recruitment for multiple factors, most of which were orchestrated by the upstream kinases of DNA-PKcs, ATM/ATR [4, 10]. Then the H1299 cells with stable expression of TG2-GFP fusion protein were pretreated with DNA-PKcs inhibitor (NU7441), ATM inhibitor (KU55933) or ATR inhibitor (VE821), after which DSB were induced. Surprisingly, it was found that nuclear translocations of TG2 were mostly abrogated in cells treated with NU7441, but remained normal in cells treated with either KU55933 or VE821 (Fig. 2 C, D). It was also confirmed that TG2 nuclear translocation was abrogated in DNA-PKcs deficient M059J cells, but not in the M059K cells (Fig. 2E). These data suggested that DNA-PKcs or its substrates played important roles in mediating nuclear translocation of TG2. Because DNA-PKcs was known to function as a core kinase during DSB repair, we hypothesized whether TG2 could be phosphorylated by DNA-PKcs during the process. By using a phosphor-SQ/TQ antibody, phosphorylation of TG2 was detected in the immunoprecipitated protein complex with TG2 specific antibody, in response to DNA damage (Fig. 2 F). Then we performed an online analysis with an NetPhos 3.1 software, and found that TG2 could to be possibly phosphorylated at total of 48 sites, among which Ser68 (S68) and Tyr162 (T162) exhibited the highest rates for the phosphorylation by DNA-PKcs (Table S4). Then we generated S68A, T162A and S68A + T162A mutant constructs, and found that TG2 with T162A mutant loss the capacity of nuclear translocation after DSB (Fig. 2 H, I). Together, these findings suggested that DNA-PKcs dependent TG2 phosphorylation at T162 site provide a critical step for its nuclear translocation and directly responding to DSB.

### TG2 directly participated in DNA damage repair after its enrichment at DSB sites

**Fig. 3:**
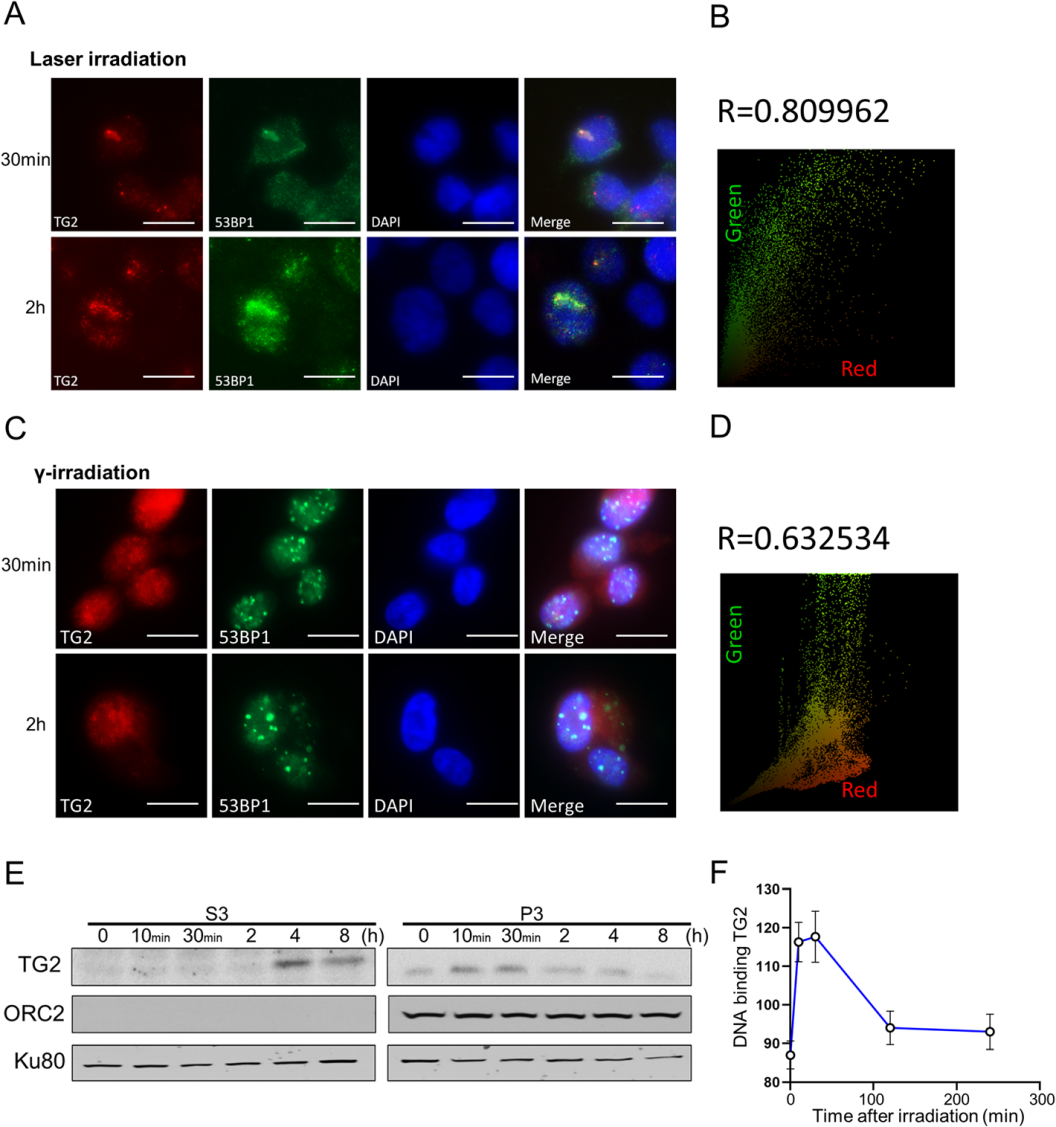
Nuclear TG2 was recruited to DSB sites. **A**: Representative images for immunofluorescence staining of TG2 (Red) in the HT-1080 cells with 53BP1-GFP stable expressions at 30 min and 2 h after laser irradiation. Scale bar, 20 μm. **B**: colocalization of TG2 and 53BP1 was performed by Image Pro Plus 6.0 software. **C**: Representative images for immunofluorescence staining of TG2 in the same HT-1080 cells with 53BP1-GFP stable expressions after 2 Gy γ-irradiation. Scale bar, 20 μm. Different from Panel A, here, the cells were analyzed in immunofluorescence staining assay of TG2 after extraction of free TG2 with CSK buffer. **D**: colocalization of TG2 and 53BP1 was performed by Image J software. **E**: Total Nuclei proteins were isolated and lysed in a no-salt buffer. Afterward, the soluble nuclear proteins (S3) were separated through low speed centrifugation. The chromatin enriched proteins were further isolated from the final pellet of soluble nuclear proteins (P3). Both soluble proteins and chromatin binding proteins were subjected to SDS page assay, and were immune-blotted by TG2, ORC2 and Ku80 antibodies. **F**: Chromatin enriched proteins were quantified through image J software to detect the levels of DNA binding

In order to determine whether TG2 directly participated in DNA damage repair, the details of activation process, were investigated spatially and temporally. Briefly, a laser irradiation was performed to induce *in situ* DSBs inside nucleus. Results indicated that TG2 immediately gathered to DSB sites after laser irradiation, and that it colocalized with 53BP1, a critical component of DSB repair complex (Fig. 3 A, B). Because abundant TG2 was enriched in nucleus after excessive DSBs induced by γ-irradiation, cytoskeletal extraction buffer was specially used to release the free TG2 for clearly tracing and observing the microscopic localization of TG2. Foci formation of TG2, together with 53BP1, was clearly found in nucleus after γ-irradiation (Fig. 3 C, D). In order to confirm whether TG2 directly bound with DNA, chromatin-binding proteins were specifically isolated through chromatin fraction assay and subjected to SDS-PAGE gel analysis. Remarkably, our data revealed that TG2 bound with DNA as early as 30 min after DSB induction (Fig. 3E, F), suggesting that the nuclear TG2 could directly bind to the damaged DNA site, at which it started to promote activations of DNA damage repair.

### TG2 directly bound to TOPOIIα protein with TGase domain during process of DNA damage repair

**Fig. 4:**
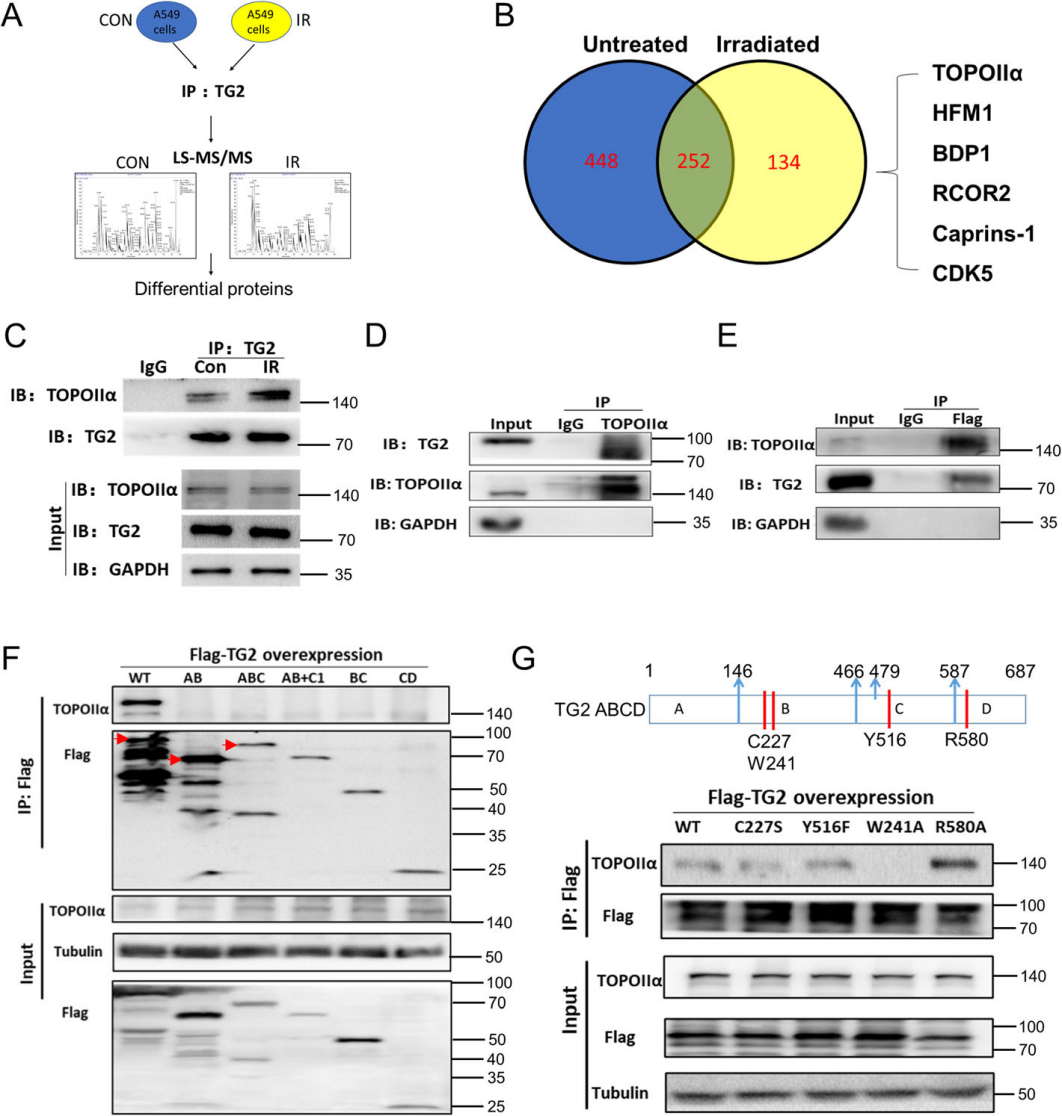
Nuclear TG2 bound to TOPOIIα after aggregations to DSB sites. **A**: The workflow of immunoprecipitation and mass spectrometry assays to identify TG2 interacting proteins. **B**: Venn diagram summarized the results of differentially enriched proteins in irradiated A549 cells, compared with the untreated A549 cells. **C**: Whole cell lysates were prepared from the A549 cells at 0 and 0.5 h after exposure to γ-irradiation, which were subjected to immunoprecipitation with anti-TG2 antibody and immunoblotted with TOPOIIα primary antibody. **D**: Whole cell lysates were prepared from the 293T cells transfected with both Flag-tagged TG2 and TOPOIIα constructs after IR (8 Gy) for 30 min, which were subjected to immunoprecipitation with anti-TOPOIIα antibody and immunoblotted with both anti-TG2 and anti-TOPOIIα antibodies. **E**: In similar, whole cell lysates were also prepared from the 293T cells transfected with FLAG-tagged TG2 and TOPOII α constructs after IR (8 Gy) for 30 min, which were subjected to immunoprecipitation with anti-Flag antibody and immunoblotted with both anti-TOPOIIα and anti-TG2 antibodies. **F**: Representative images of immunoprecipitation assay for various 293T cells transfected with each TG2 fragments (AB, ABC, AB + C1, BC, CD) and TOPOIIα. **G**: Representative images of immunoprecipitation assay for various 293T cells transfected with each TG2 mutant fragments (WT, C227S, Y516F, W241A, R580A) and TOPOIIα. Quantification analyses were performed based on the ratio of TOPOIIα and Flag-TG2 mutants density

After realizing that TG2 rapidly aggregated to DSB sites for DNA damage repair (Figs. 1 and 2), details of activations along with its controlling mechanism were investigated. Here, mass spectrometry analysis was performed with the protein complex immunoprecipitated with TG2 antibody in order to screen the potential targets interacting with TG2 (Fig. 4 A). From the original IP-MS data, we compared the protein complex immunoprecipitated with TG2 specific antibody in control group with that in the radiation group, from which 134 proteins were identified in radiation group, but not in control groups. These data indicates that TG2 potentially bound to these proteins after radiation-induced DNA damage (Fig. 4B, Table S5, 6). Then Regarding to the functions of these proteins, 6 candidates were selected for further analyses and validations, including TOPOIIα, HFM1, BDP1, RCOR2, Caprins-1 and CDK5 (Fig. 4B). Among them, TOPOIIα was abundantly enriched based on the results that several of its fragments were identified in mass spectrometry analysis (Table S5). Furthermore, immunoprecipitation (IP) data showed that endogenous TOPOIIα was found to bind with TG2 only in response to DNA damage, while little binding observed in A549 cells under normal condition (Fig. 4 C). To further confirm this data, TOPOIIα expressing plasmid and flag-TG2 expressing plasmid were co-transfected into 293T cells. Next, IP was performed in 293T cells after 8 Gy irradiation. Consistently, TOPOIIα was detectable in IP extracts with flag antibody, and TG2 was also detectable in the IP extracts by using TOPOIIα specific antibody (Fig. 4D, E). Next, the specific TG2-TOPOIIα interaction was investigated precisely. Known from previous studies, TG2 was a multifunction protein with complicated conformation changes presented by its different domains[20]. In order to investigate which functional domain of TG2 specifically bound with TOPOIIα, a group of expression vectors of Flag-tagged TG2 fragments were constructed and transfected into the TG2 low-expressing H1299 cells (Fig. S4A: full length, Fragment AB, ABC, AB + C1, BC, CD; Table S3) [21]. Next, IP were performed with suing specific anti-Flag primary antibody. Briefly, none of TG2 fragments was found to bind with TOPOIIα (Fig. 4 F), indicating it was possible that the interaction with TOPOIIα could require some cooperation from multiple domains. Based on previous studies[22], additional expression vectors with various forms of TG2 with loss function mutations were constructed, including W241A, C277S, R580A, Y516F mutants (Fig. S4B; Table S3). Among them, the efficacy for binding between W241A mutant of TG2 and TOPOIIα reduced significantly when compared to that for binding between wild type TG2 and TOPOIIα (Fig. 4G), while other mutants of TG2 did not affect their TOPOIIα binding efficacies, indicating that TGase function could be responsible for the direct interaction between TG2 and TOPOIIα. To find out whether these mutants affect cellular sensitivity to DNA damage treatments, colony formation assay were performed in H1299 cells with these TG2 mutants overexpression. Our data showed that the over-expression of TG2 W241A mutant lost the function to improve colony formation efficacy, while over-expressions of all other mutants were same as wild-type TG2 (Fig. S5).

### TOPOIIα is critical for mediating TG2 promoted DNA damage repair

**Fig. 5:**
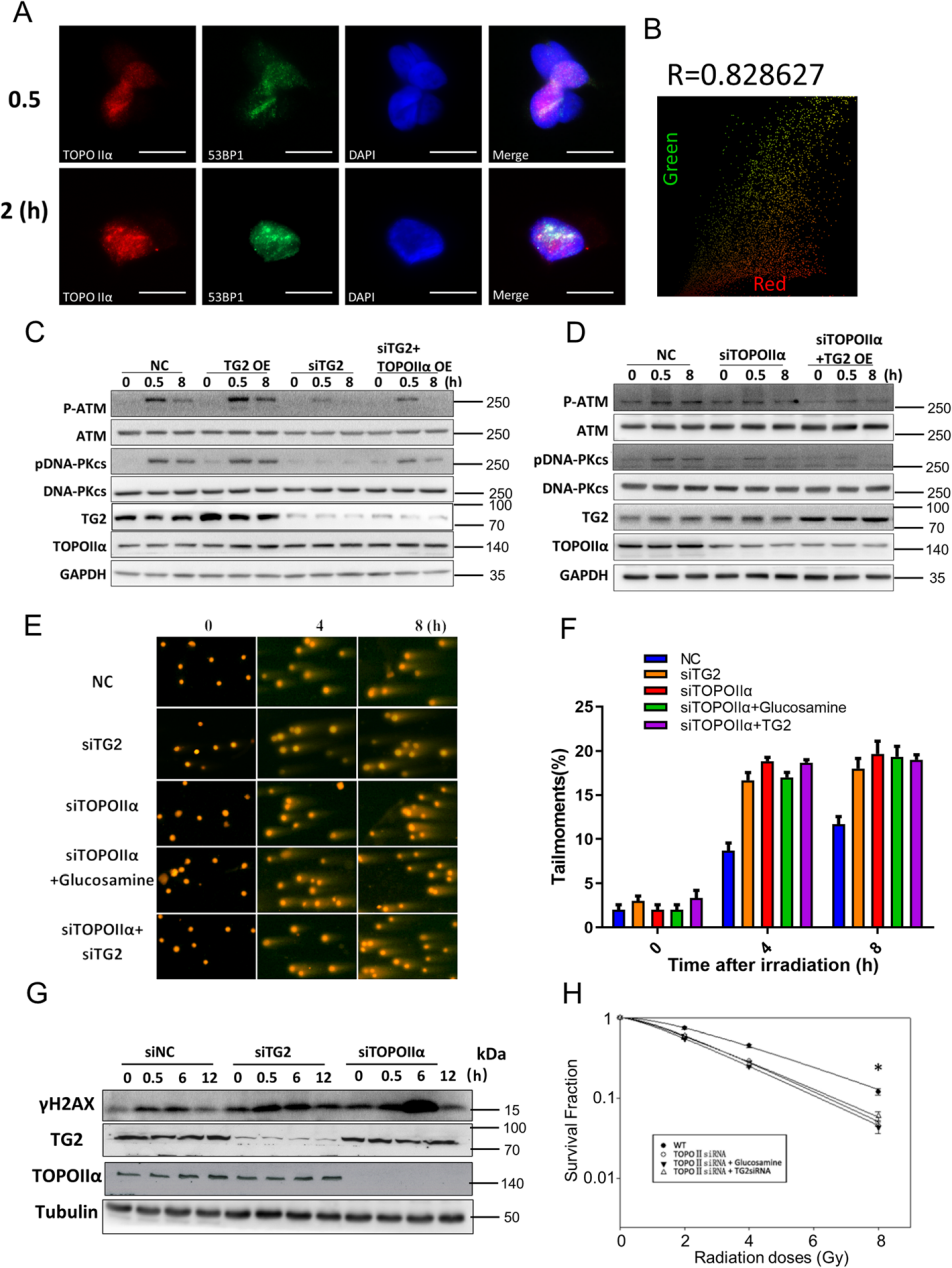
TOPOIIα is critical for mediating TG2 promoted DNA damage repair. **A**, **B**: Representative immunofluorescence of TOPOIIα and 53BP1 at DSB sites, in the HT1080 cells with 53BP1 expression after laser micro-irradiation. Colocalization analysis was also performed by using Image Pro Plus 6.0 software. **C**: Whole cell lysates were harvested from the A549 cells transfected with TG2 construct/TG2 siRNA/TG2 siRNA + TOPOIIα at the indicated time points of IR (8 Gy) treatments. Whole cell lysates were blotted with pDNA-PKcs, DNA-PKcs, pATM and ATM antibodies. **D**: Whole cell lysates were harvested from the A549 cells transfected with TOPOIIα siRNA/ TOPOIIα siRNA + TG2 construct at the indicated time points of IR (8 Gy) treatments. Whole cell lysates were analyzed with indicated antibodies. **E**: representative images of comet assay in cells with TG2 and /or TOPOII α knockdown after irradiation. **F**: Quantitate analysis of comet assay showed the tail moment of A549 cells transfected with TOPOII α siRNA and siTG2 after treatment of ionizing radiation (IR). **G**: Immunoblot of γ-H2AX in original A549 cells, and the A549 cells with either TG2 knockdown or TOPOIIα knockdown after IR. **H**: colony formation assay of cells with TG2 and /or TOPOII α knockdown after 0, 2, 4, 6 Gy irradiation. *P < 0.05

Then we determined whether TOPOIIα is the key downstream factor mediating the effects of TG2 in DDR. Consistent with TG2, TOPOIIα was also observed to bind to the broken DNA after induction of DSB (Fig. 5 A, B). These findings also supported that TG2 directly bound with TOPOIIα at DSB sites. Next, we determined whether TG2-TOPOIIα interaction was a necessary step to mediate DNA damage repair. For the DNA damage response, overexpression of TOPOIIα protein in TG2 knockdown cells partially restored levels of their phosphorylation and activations (Fig. 5 C), while overexpression of TG2 in the TOPOIIα knockdown cells showed minor effect on the activations of DNA-PKcs or ATM (Fig. 5D). Then we performed comet assay to evaluate the overall DNA damage, results indicated that DNA damage repair was inhibited in the cells with either TG2 or TOPOIIα knockdown, and no additive effect was observed in cells with double knockdown of TOPOIIα and TG2 (Fig. 5E, F). In addition, we treated TOPOIIα with a TG2 inhibitor, glucosamine, and no further additive effects was observed (Fig. 5E F). Alternatively, either knockdown of TG2 or TOPOIIα resulted in unrepaired DNA damage revealed by γH2AX (Fig. 5G). Knockdown of TG2 and TOPOIIα did not show additive effects on cell survival, compared to single knockdown of TG2 or TOPOIIα (Fig. 5 H). Thus, these results suggested that interacting with TOPOIIα was a key step for TG2 to elucidate the new mechanism of DSB repair.

### Inhibition of TG2-TOPOIIα interaction inhibited DSB repair to enhance cancer cell therapy

**Fig. 6:**
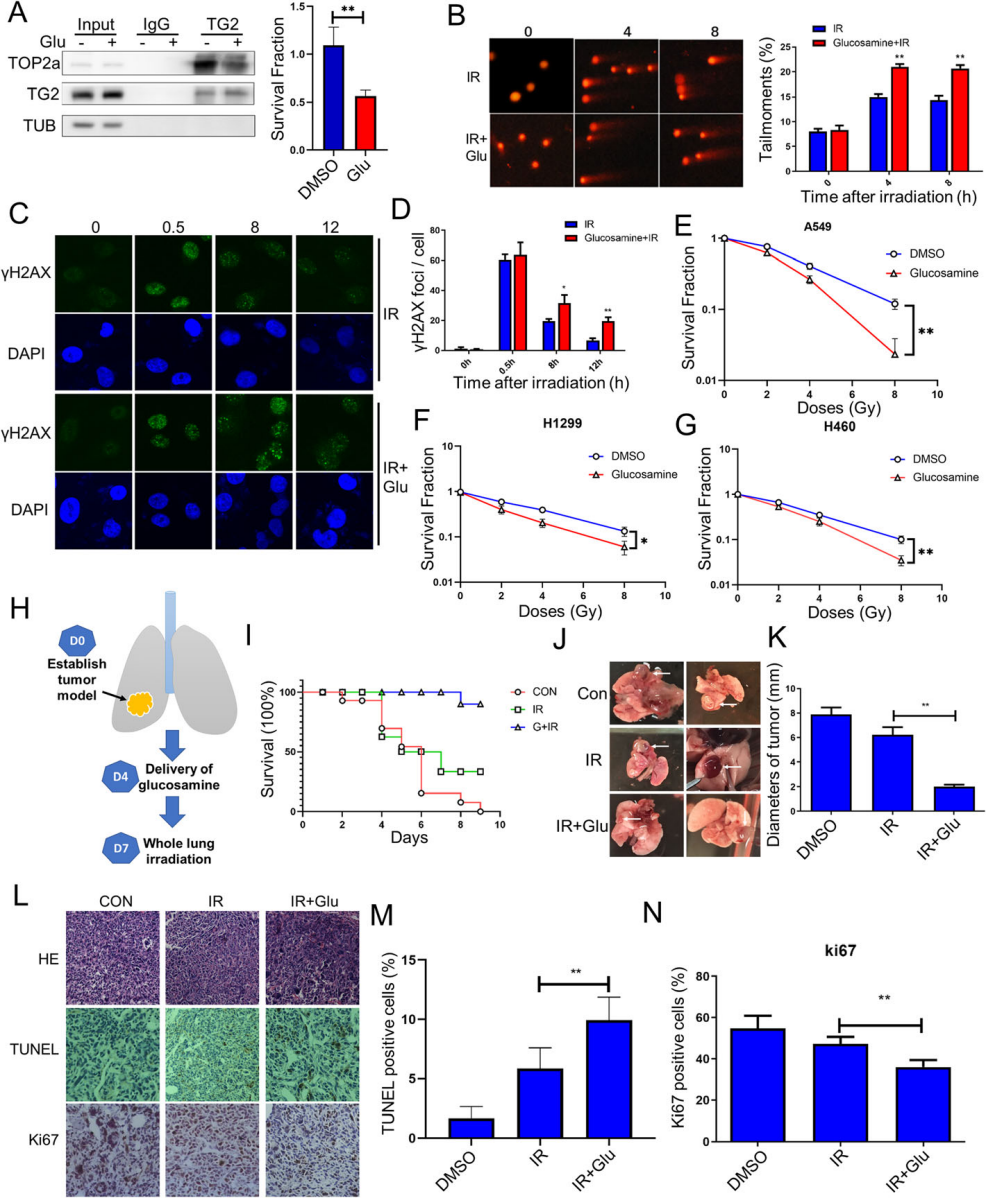
Inhibition of TG2- TOPOIIα interaction with Glucosamine treatment improved the efficacy of radiotherapy in lung cancer. **A**: Immunoblots of anti-TG2 IPs. Whole cell lysates were prepared from the irradiated A549 cells (8 Gy) with/without glucosamine treatment, which were subjected to IP with anti-TG2 antibody and immunoblotted with TOPOIIα antibody. B: Representative images of comet assay of 8 Gy irradiated A549 cells in glucosamine (5mM) or DMSO treated groups. Scale bar, 50 μm. Results were further and quantitatively analyzed. **C**,** D**: Representative images for immunofluorescence staining of γ-H2AX in DMSO or glucosamine (5mM) treated A549 cells after different times of irradiation. Scale bar, 20 μm. **E**-**G**: Colonogenic assay was used to detect the colony formation of glucosamine or DMSO treated A549 cells, H1299 cells and H460 cells after 0, 2, 4 or 8 Gy irradiation. *P < 0.05. **P < 0.01. **H**: A flow diagram of combined treatments of both glucosamine and radiotherapy. LLC cells (2 × 10^5^cells) mixed in 25 ul Matrigel were injected directly into the right leaf of lung on the first day (D0). The tumor bearing mice were treated with glucosamine (150 mg/kg/d) from D4 to D7 for 3 days, after which the whole lung areas were irradiated with a single dose of 15 Gy. **I**: Tumor bearing mice were treated with the combined treatments of whole lung irradiation and glucosamine (*n* = 10). Survival rate of treated mice was monitored for up to 10 days after radiotherapy. **J**, **K**: Representative images of the isolated tumors in different animal groups (*n* = 8), compared with summarized diameters of these isolated tumors. **L**: Representative images for HE staining, TUNEL and Ki67 IHC staining of tumors in each group (*n* = 8). **M**, **N**: Quantification for the levels of both TUNEL and Ki67 staining on lung tumors after radiotherapy with/without glucosamine treatments. (*n* = 8) *P < 0.05 and **P < 0.01 versus single radiation group

Based on our findings, how to inhibit such interaction was tested for the purpose to inhibit DNA damage repair for overcoming the cancer resistance. Therefore, candidate chemicals were broadly searched, including TGase inhibitors of glucosamine and KCC009, competitive amine inhibitor, reversible inhibitors as well as irreversible inhibitors, etc. Among these blockage candidates, glucosamine was found to have significant effect for inhibiting TG2-TOPOIIα interaction in irradiated A549 cells (Fig. 6 A, B). Next, glucosamine was further analyzed to determine the effect on inhibition of DNA damage repair. Briefly, our results indicated that significant levels of comet tail content still remained higher in the glucosamine (5mM) pretreated cells than that in the DMSO treated cells after DNA damage (Fig. 6B). The inhibition of TG2-TOPOIIα interaction with glucosamine also resulted in significant collections of unrepaired DNA damage reflected by the numbers of γH2AX foci (Fig. 6 C, D). Then, the exact levels of cell death were specifically analyzed in various cells to determine whether they had different sensitivities to radiation induced cell deaths. Results indicated that survival fractions in glucosamine-treated A549, H1299 and H460 cells significantly reduced when compared with the DMSO treated cells after irradiation (Fig. 6E, F, G). We also showed that glucosamine had no influence on the colony formation efficacy in TG2 KD A549 cells (Fig. S6A), which confirmed that the glucosamine increased radiation-induced cell killing in a TG2 dependent manner. Glucosamine combined with radiotherapy also inhibited the activation of DNA damage signaling pathway, including phosphorylation of ATM, ATR and DNA-PKcs (Fig. S6B, C, D, E). Together, these results confirmed that glucosamine inhibited TG2-TOPOIIα interaction, which could be potentially used to increase cancer cell death when combined with DNA damage relative therapies.

### Glucosamine inhibited TG2-TOPOIIα interaction to inhibit DSB repair and enhanced therapeutic effects of radiotherapy on lung cancers

**Fig. 7:**
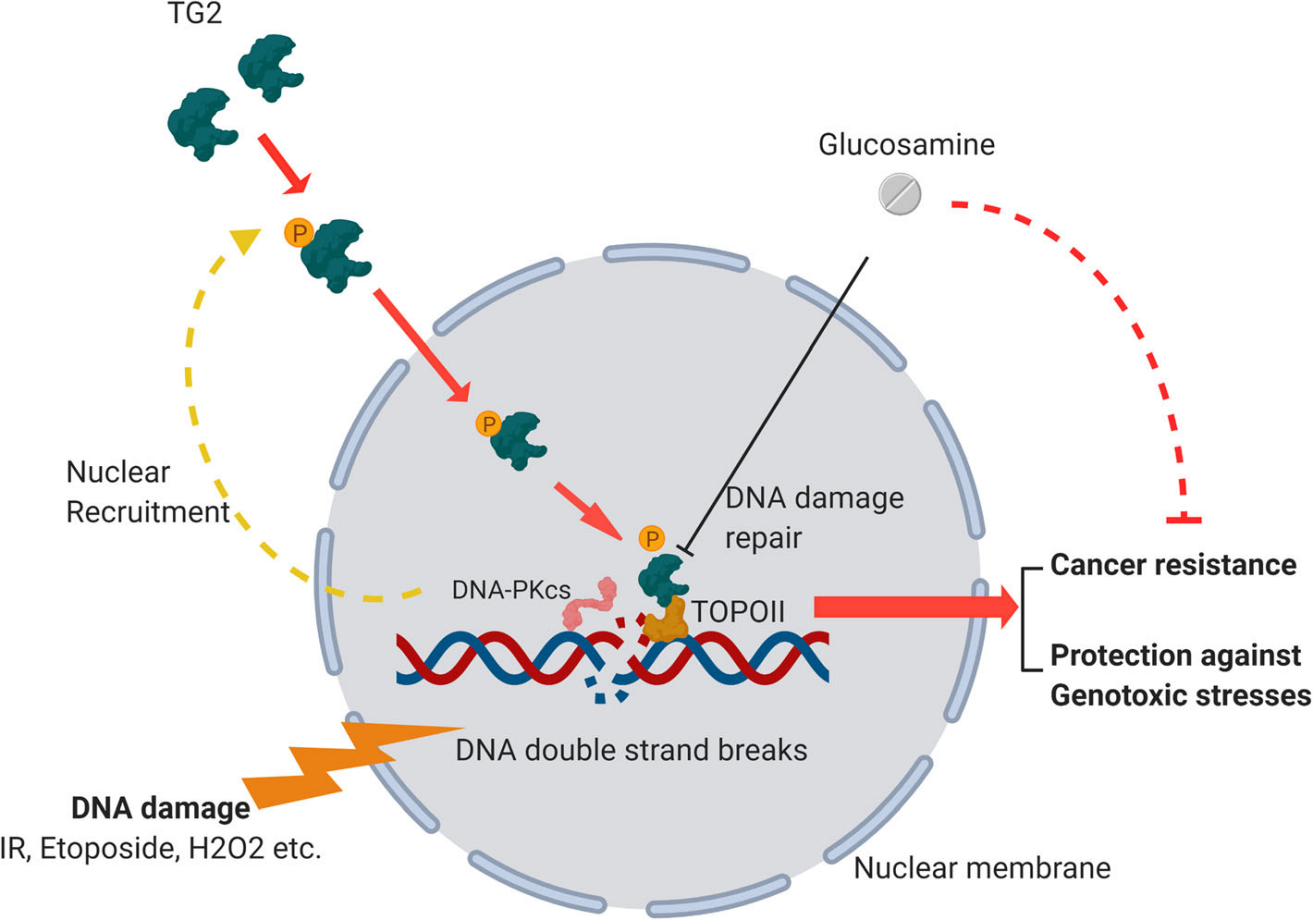
A working model to illustrate how TG2 dynamically takes part into activities of DSB repair. Briefly, in response to the extensive DNA double strand breaks, TG2 is initially activated by phosphorylation by DNA-PKcs. Then the phosphorylated TG2 translocates from cytoplasm into nucleus, where it is further recruited or actively aggregates onto DSB sites and binds to TOPOIIα. TG2-TOPOIIα interaction is necessary for DSB repair and enhance the radiotherapy of lung cancer

Next, the inhibition of TG2-TOPOIIα interaction and DNA damage repair with glucosamine was carefully investigated as potential application of targeting strategy to conquer cancer resistance. For this goal, an *in situ* lung cancer xenograft model was established through injecting LLC cells into the inferior lobe of lung tissues of C57BL/6 mice, in order to study the impacts of combinational treatments of glucosamine and radiotherapy in vivo (Fig. S7A, B). Briefly, established mice of tumor bearings were pretreated with glucosamine, and then subjected to the thoracic radiotherapy at a single dose of 15 Gy (Fig. 6 H). Compared with those tumor bearing mice only treated with thoracic radiotherapy, glucosamine (150 mg/kg/d) pretreatment significantly increased the survival rate of tumor bearing mice (Fig. 6I). The average decreases of diameters of tumors were also shown in the group treated with both glucosamine and thoracic radiotherapy, when compared to single radiotherapy group (Fig. 6 J, K). Moreover, TUNEL positive cells had significant increases in tumors of mice with combined treatments, compared with the single radiation group (Fig. 6 L, M). On the other hand, levels of proliferating cells (Ki67 positive) were also significantly reduced after combined treatments (Fig. 6 L, N). Together, these findings strongly suggested that inhibition of TG2-TOPOIIα interaction with glucosamine could be a potent strategy to overcome the resistance to radiotherapy and possibly the other types of DNA damage relative therapies (Fig. 7).

## Discussion

Our findings could be regarded as a breakthrough to realize the direct role of TG2 during DSB, which could be helpful to understand the machinery for any living organisms to cope with genotoxic stresses, as well as the clinical practice to overcome cancer cell resistances to chemotherapy and radiotherapy.

Recently, TG2 was also suggested to be a protein relative to DNA damage treatments in other reports. For example, DNA damage inducible chemicals such as N-methyl-N’-nitro-N-nitrosoguanidine and Etoposide, increased the expression level of TG2 through ATM and NF-kB dependent transcription[11]. TG2 was found to be necessary for protecting against DNA damage induced cell death by suppressing the phosphorylation of p53 [12]. The above literatures raised an important question that TG2 might be a unique component responsive to DNA damages. However, these reports did not supply the important information about whether TG2 is physically involved DNA damage repair and what kind of functions it actually performs is still lacking.

Our study brought new understanding on the roles of TG2 during the DNA damage repair process in response to extensive cellular stresses, which is, how TG2 responds and how TG2 is activated by the upstream signaling activities. Under normal circumstances, TG2 usually located in cytoplasm, aside from the 5–7 % of TG2 inside the nucleus[17, 23]. In our study, we observed that TG2 was specially activated when it was in the dynamic state of phosphorylation and nuclear translocation. TG2 was found to accumulate in nucleus at 0.5 to 4 h, however, there is an increase of cytoplasmic TG2 at 8 h after irradiation, which could result from two reasons. On one hand, irradiation of cells induced the expression of overall TG2 expression in extract of whole cells (Fig. S2B), which peaked at 8 h post-irradiation. The indued expression of TG2 might accumulate in cytoplasm when nuclear TG2 is abundant. On the other hand, TG2 has both nuclear localization signal (NLS) and nuclear export signal (NES), which made it possible to shuttle between cytoplasm and nucleus [20]. It is possible that nuclear TG2 translocated into cytoplasm after performing its function in DNA damage repair. Moreover, TG2 nuclear translocation was only found in response to DSB, the most severe DNA damage, instead of SSB or DNA crosslink [24]. This finding indicates some unknown signals relative to DSB may be uniquely induced for active mobilization of TG2 nuclear translocation.

Cells utilize different repair machinery to repair distinct types of DNA damage, for example, NHEJ and HR mainly accounts for DSB repair [25], and evoke a protein network involving sensors, transducers, and effectors[26]. Therefore, we focused on the key upstream factors in these two pathways and found that DNA-PKcs was responsible for TG2 nuclear translocation. From this finding, we could hypothesize that DSB resulted in the phosphorylation of H2AX, as well as DNA-PKcs, which further caused TG2 phosphorylation and nuclear translocation (Fig. 7). Furthermore, we depict that T162 sites of TG2 may be phosphorylated with DNA-PKcs, which is critical for TG2 activation.

The finding in this study also helps to pursue future studies in several aspects. Firstly, TG2-TOPOIIα interaction provided a potential target in promoting DNA damage repair. However, how this interaction modifies TOPOIIα function remains unclear. It has been reported that TG2 usually performed its functions through protein crosslink, GTP binding, or as kinase[27–29]. The modification on TOPOIIα activity or other non-enzymic functions will be investigated. Moreover, whether TG2 activation involves in NHEJ or HR machinery still needs to be determined. Together, these considerations will be included in our future study, which will provide additional realization on both mechanism and applicable targeting sites for controlling TG2-TOPOIIα interaction.

One of the potential applications is to utilize the critical role of TG2 in DNA damage repair to overcome cancer resistance to various therapies. After TG2 mobilization signals are fully characterized, we will be able to deliver signals to trigger TG2 nuclear translocation or recruitment to promote DNA damage repair. Alternatively, either blocking TG2 translocation and recruitment, or blocking the site of TG2-TOPOIIα interaction, will provide further killing effects in cancer cells. For instance, in the present study, we found that glucosamine increased the effects of radiotherapy for lung cancers by inhibiting the interaction of TG2 and TOPOIIα. TG2 expression level was also aberrantly elevated in other types of cancers, such as colorectal cancer, breast cancer, melanoma, and ovarian cancer[30–34]. Inhibition on TG2-TOPOIIα interaction in these types of cancers may broadly overcome their resistances to DSB related therapies[35–37]. Our study has the opportunity to combine TG2 targeting therapy with radiotherapy or other DNA damage-related chemotherapies, including cisplatin, Etoposide, or 5-FU. In addition, novel function sites and targeted inhibitors should be screened to enlarge the clinical applications.

(Discussion: 773 words)

## Conclusions

In conclusion, our findings uncovered a common mechanism regarding TG2 mobilization in response to DSB in manner dependent on DNA-PKcs as a novel factor promoting DNA damage. Nuclear TG2 enriched at DSB sites interacted with TOPOIIα to promote DNA damage repair. Glucosamine, which has potential in clinical applications, was found to enhance the therapeutic effects of radiotherapy by inhibiting the interaction of TG2-TOPOIIα and increasing DNA damage-induced cell death (Fig. 7).

## Supplementary Information

**Additional file 1: Supplementary Figure S1.** Bioinformatic analysis of patient data from cancer patients from TCGA. A: Heatmap of differently expressed genes in patients sensitive or resistant to radiotherapy. B, C: TGM2 was found to be negatively correlated with both overall survival (OS) and progression-free survival (PFS) in the patients with cancers, including lung cancer, colorectal cancer, head and neck carcinoma. D: image of tissue array derived from clinical lung cancer patients. E: representative images of TG2 high expression and low expression tissues. F: overall survival of lung cancer patients of TG2 high expression as well as low expression.

**Additional file 2: Supplementary Figure S2.** High expressions of TG2 in lung cancer cells were relative to the activities of DNA damage. A: TG2 expression levels in the extracts from A549, H1299, H460, H358, H1975, LLC and BEAS-2B cells. B: TG2 expression at different times after irradiation. C, D: colony formation efficacy in TG2 knockdown cells with CRISPR Cas9 system, and in cells with TG2 overexpression (OE) after irradiation. E: Cells apoptosis was detected at 24 h after irradiation with an Annexin V/PI double staining kit. F: A549 cells with TG2 KD were treated with etoposide and survival fraction was quantified. G: Colony formation efficacy of both TG2 KD A549 cells and original A549 cells upon CPT treatments.

**Additional file 3: Supplementary Figure S3.** A: Cellular localization of TG2 in the H1299 cells with stable expression of transfected TG2-GFP. All cells were treated with different types of DNA damaging agents, including etoposide (100ug/ml), CPT (1µM), 4NQO (50µM), H_2_O_2_ (10mM) and UVB (600 J/cm^2^). B: the ratio of TG2 inside nucleus and cytoplasm was analyzed with Image J software. **P < 0.01.

**Additional file 4: Supplementary Figure S4.** Summary of information for the plasmids with expressions of various TG2 normal and mutant fragments. A: Schematic structure for full length of TG2, and the normal fragments including AB, ABC, AB + C, B + C, CD. B: Plasmids encoding normal fragment clones of TGM2, W241A, C277S, R580A, Y516F in pLenO-GTP were constructed by Biolink Biotechnology(Shanghai) Co.,Ltd.

**Additional file 5: Supplementary Figure S5.** Loss function mutation of TGase domain had no capacity to promote cell survival upon DNA damage. A: survival fractions of the H1299 cells with overexpression of TG2. B-E: Reduced levels survival fractions for the H1299 cells transfected with TG2 W241 mutant (B), C227S mutant (C), Y516F mutant (D) and R580A mutant (E).

**Additional file 6: Supplementary Figure S6.** Glucosamine did not increase the cellular sensitivity in TG2 KD cells and inhibited activation of DDR. A: colony formation efficacy of irradiated TG2 knockdown A549 cells (0, 2, 4, 8 Gy) pretreated with 5mM glucosamine. B: WB analysis of DDR signaling pathway in cells pretreated with glucosamine. C-E: quantitative analysis of raw density of pATR to ATR (C), pATM to ATM (D), and pDNA-PKcs to DNA-PKcs (E) in irradiated cells with/without glucosamine treatment.

**Additional file 7: Supplementary Figure S7.**Establishment of the xenografted lung cancer model ***in situ***. A: Representative image of position of injection in the mouse model for establishing lung cancer xenograft. B: Representative images of the xenografted lung cancer model *in situ*.

**Additional file 8.**

**Additional file 9.**

**Additional file 10.**

**Additional file 11.**

**Additional file 12.**

**Additional file 13.**

**Additional file 14.**

## Data Availability

All the data reported by the manuscript are publicly available and the materials are also freely available.

## Abbreviations

DSB: DNA double strand break
SSB: DNA single strand break
DDR: DNA damage response
TG2: Transglutaminase 2
TOPOIIα: Topoisomerase IIα
TCGA: The Cancer Genome Atlas
LLC: Lewis lung cancer cells
IR: Ionizing radiation
UV: Ultraviolet radiation
CPT: Camptothecin
