# Supplementary figures and images for: Nuclear Transglutaminase 2 interacts with topoisomerase II⍺ to promote DNA damage repair in lung cancer cells
Source: J Exp Clin Cancer Res. 2021 Jul 5;40:224. doi: 10.1186/s13046-021-02009-2 (PMC8258933; doi:10.1186/s13046-021-02009-2)

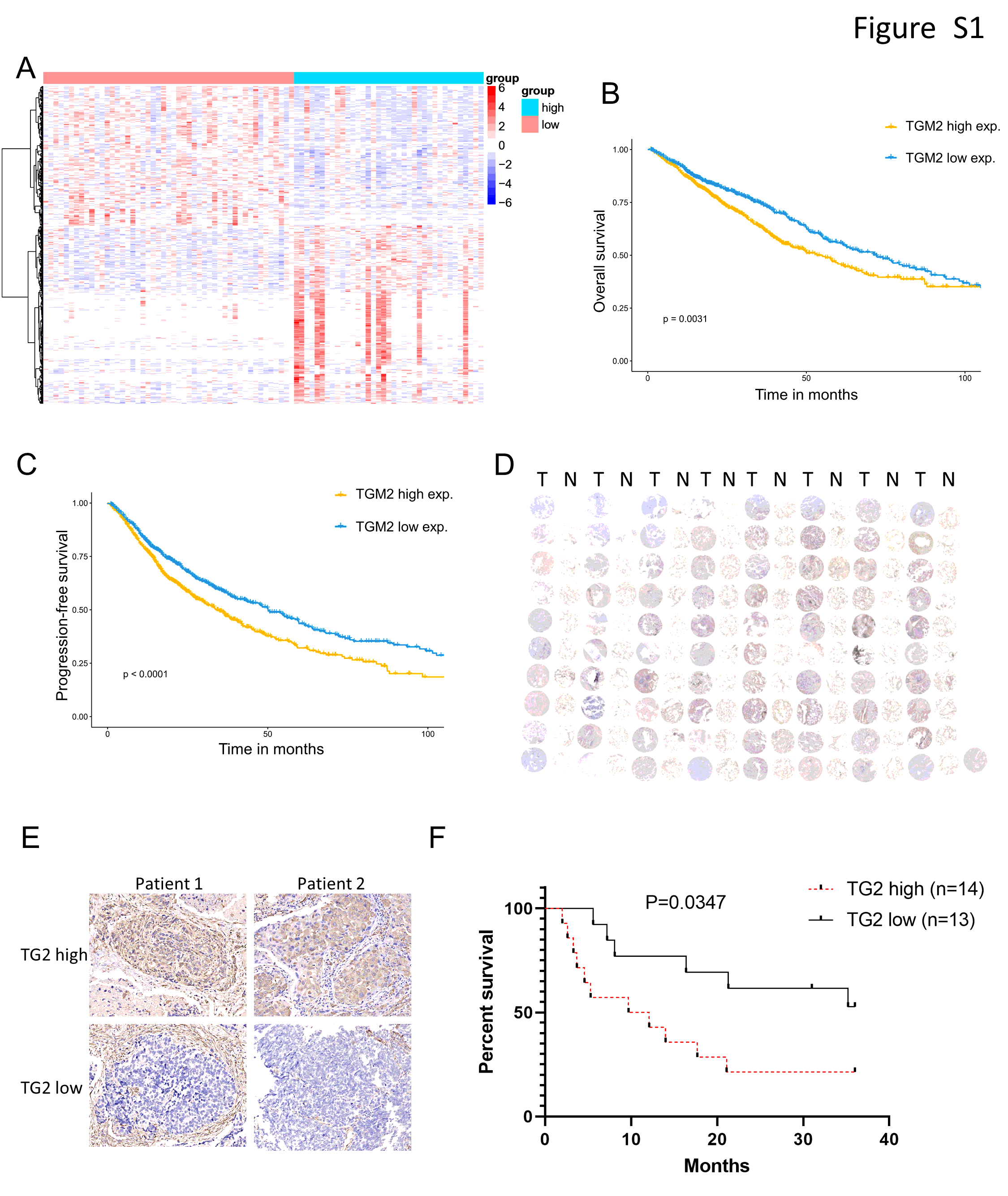

Supplement: Supplementary file 1 — Additional file 1: Supplementary Figure S1. Bioinformatic analysis of patient data from cancer patients from TCGA. A: Heatmap of differently expressed genes in patients sensitive or resistant to radiotherapy. B, C: TGM2 was found to be negatively correlated with both overall survival (OS) and progression-free survival (PFS) in the patients with cancers, including lung cancer, colorectal cancer, head and neck carcinoma. D: image of tissue array derived from clinical lung cancer patients. E: representative images of TG2 high expression and low expression tissues. F: overall survival of lung cancer patients of TG2 high expression as well as low expression. [file 13046_2021_2009_MOESM1_ESM.png]

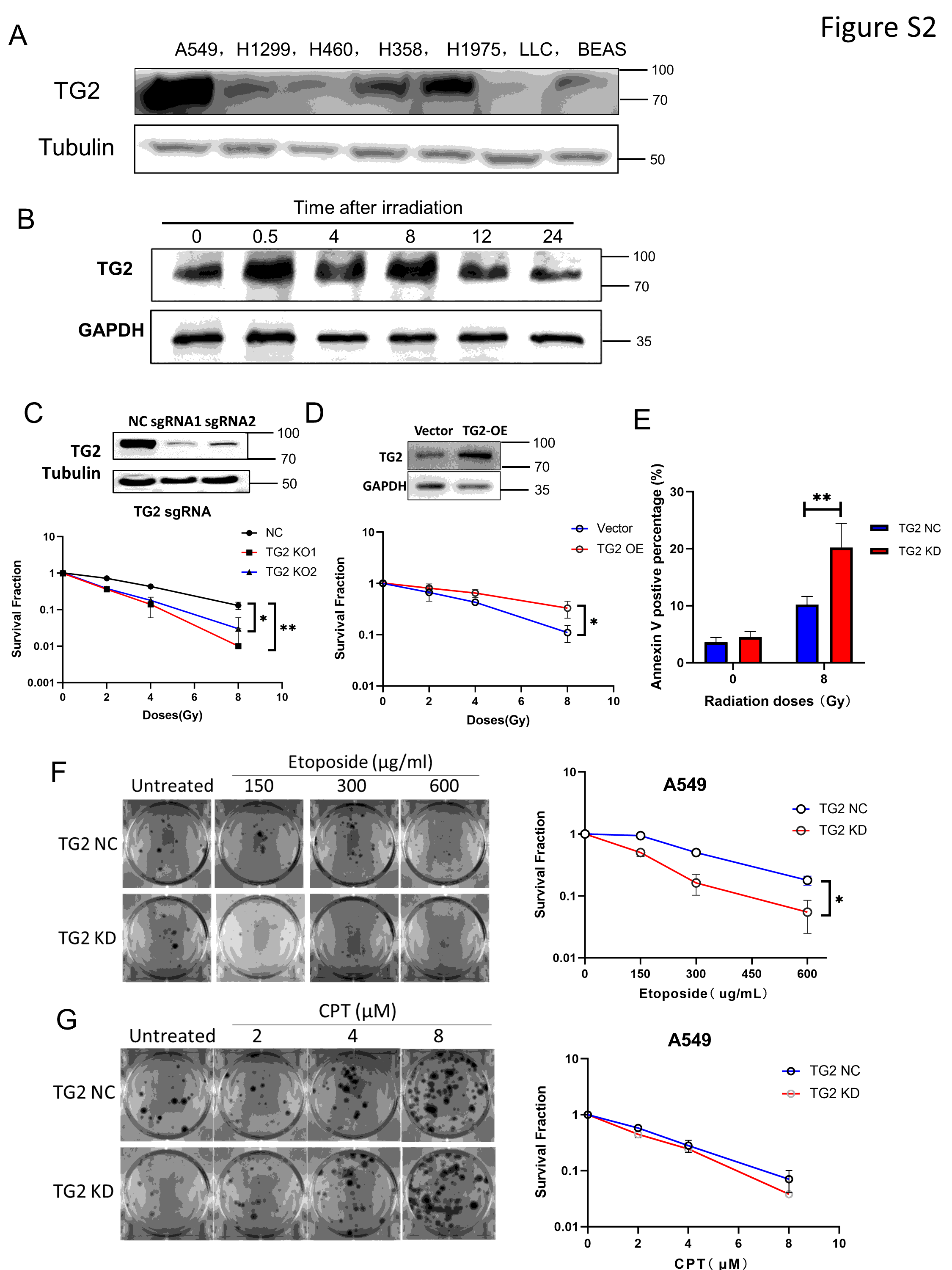

Supplement: Supplementary file 2 — Additional file 2: Supplementary Figure S2. High expressions of TG2 in lung cancer cells were relative to the activities of DNA damage. A: TG2 expression levels in the extracts from A549, H1299, H460, H358, H1975, LLC and BEAS-2B cells. B: TG2 expression at different times after irradiation. C, D: colony formation efficacy in TG2 knockdown cells with CRISPR Cas9 system, and in cells with TG2 overexpression (OE) after irradiation. E: Cells apoptosis was detected at 24 h after irradiation with an Annexin V/PI double staining kit. F: A549 cells with TG2 KD were treated with etoposide and survival fraction was quantified. G: Colony formation efficacy of both TG2 KD A549 cells and original A549 cells upon CPT treatments. [file 13046_2021_2009_MOESM2_ESM.png]

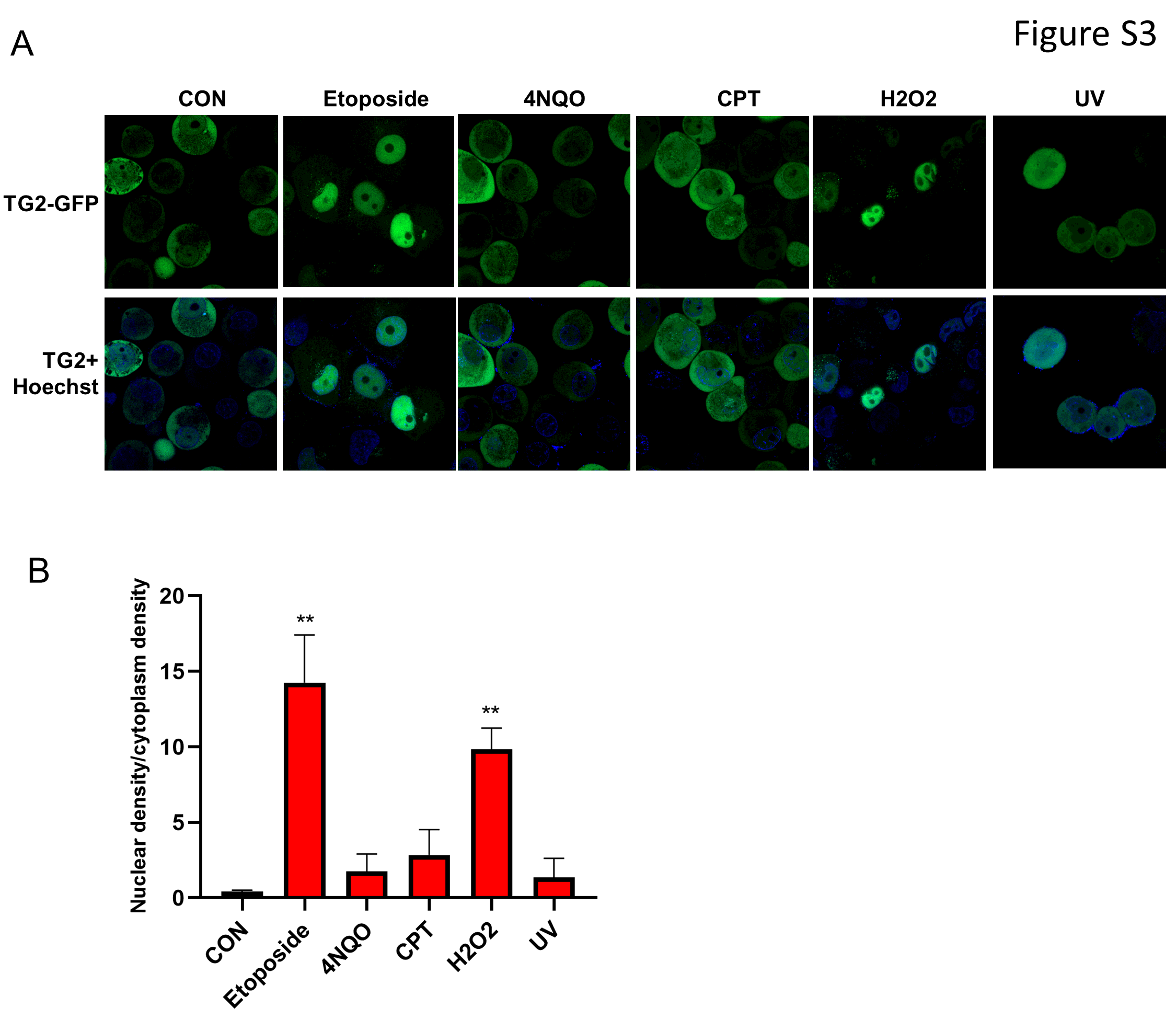

Supplement: Supplementary file 3 — Additional file 3: Supplementary Figure S3. A: Cellular localization of TG2 in the H1299 cells with stable expression of transfected TG2-GFP. All cells were treated with different types of DNA damaging agents, including etoposide (100ug/ml), CPT (1µM), 4NQO (50µM), H2O2 (10mM) and UVB (600 J/cm2). B: the ratio of TG2 inside nucleus and cytoplasm was analyzed with Image J software. **P < 0.01. [file 13046_2021_2009_MOESM3_ESM.png]

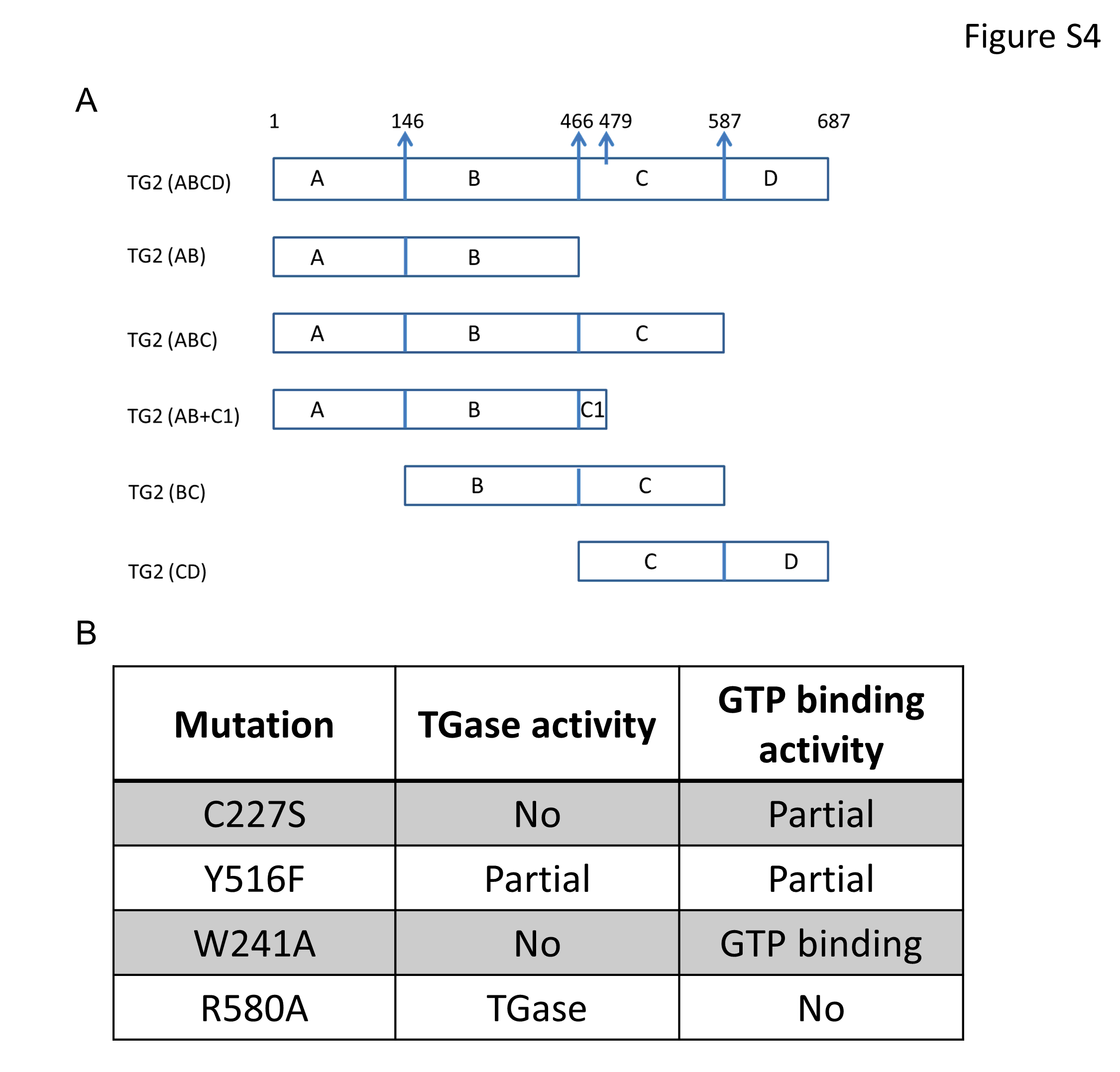

Supplement: Supplementary file 4 — Additional file 4: Supplementary Figure S4. Summary of information for the plasmids with expressions of various TG2 normal and mutant fragments. A: Schematic structure for full length of TG2, and the normal fragments including AB, ABC, AB + C, B + C, CD. B: Plasmids encoding normal fragment clones of TGM2, W241A, C277S, R580A, Y516F in pLenO-GTP were constructed by Biolink Biotechnology(Shanghai) Co.,Ltd. [file 13046_2021_2009_MOESM4_ESM.png]

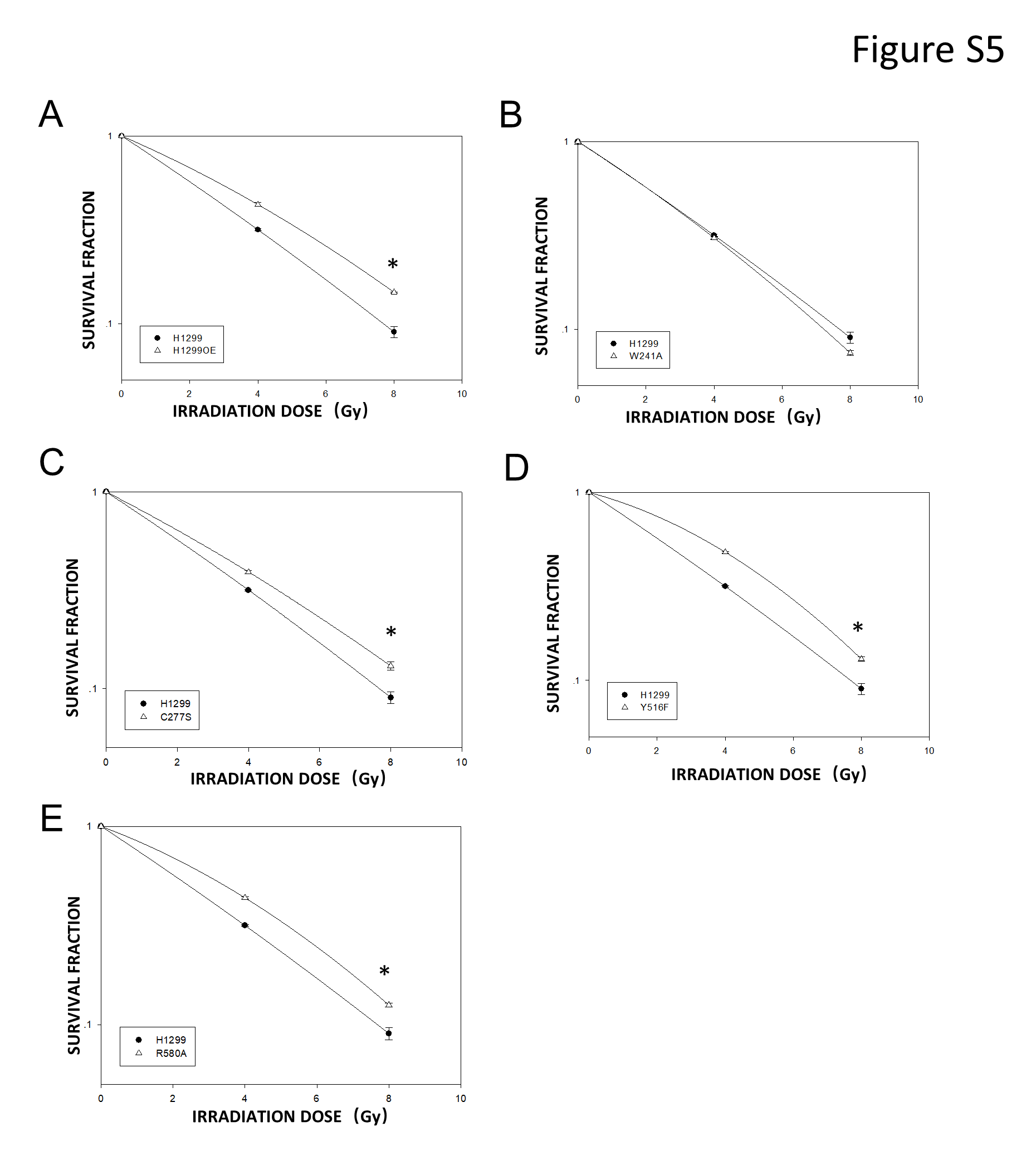

Supplement: Supplementary file 5 — Additional file 5: Supplementary Figure S5. Loss function mutation of TGase domain had no capacity to promote cell survival upon DNA damage. A: survival fractions of the H1299 cells with overexpression of TG2. B-E: Reduced levels survival fractions for the H1299 cells transfected with TG2 W241 mutant (B), C227S mutant (C), Y516F mutant (D) and R580A mutant (E). [file 13046_2021_2009_MOESM5_ESM.png]

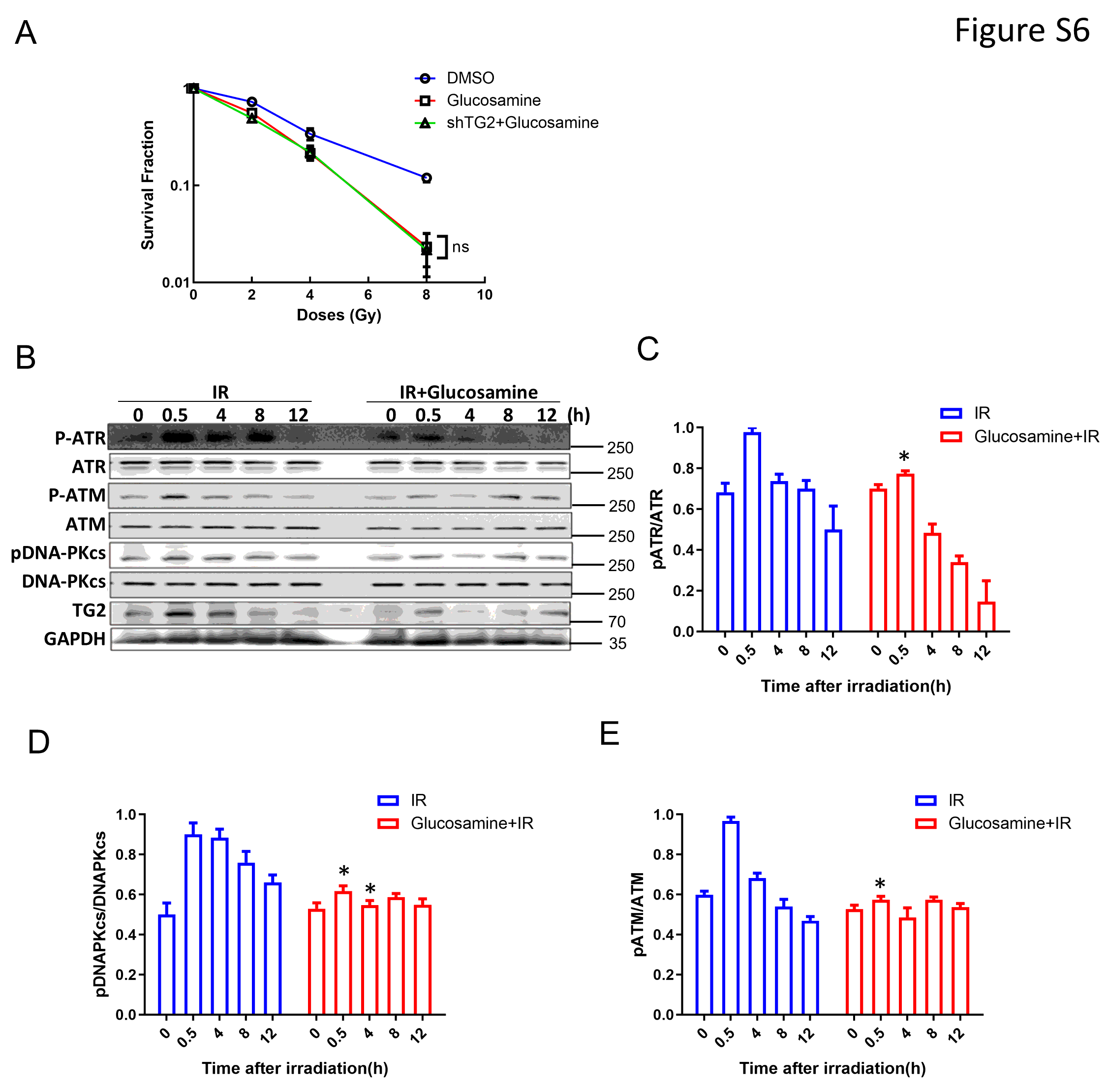

Supplement: Supplementary file 6 — Additional file 6: Supplementary Figure S6. Glucosamine did not increase the cellular sensitivity in TG2 KD cells and inhibited activation of DDR. A: colony formation efficacy of irradiated TG2 knockdown A549 cells (0, 2, 4, 8 Gy) pretreated with 5mM glucosamine. B: WB analysis of DDR signaling pathway in cells pretreated with glucosamine. C-E: quantitative analysis of raw density of pATR to ATR (C), pATM to ATM (D), and pDNA-PKcs to DNA-PKcs (E) in irradiated cells with/without glucosamine treatment. [file 13046_2021_2009_MOESM6_ESM.png]

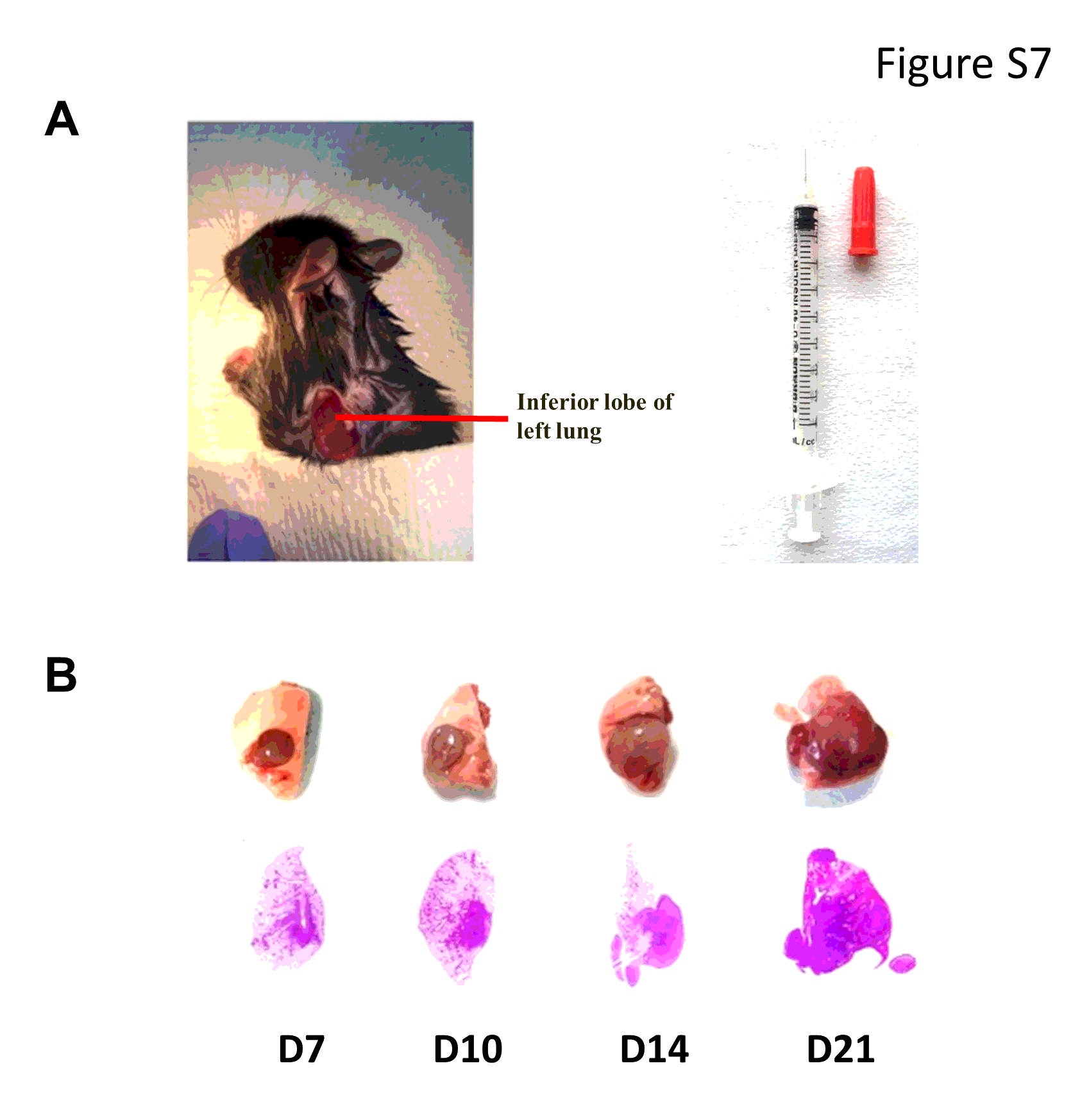

Supplement: Supplementary file 7 — Additional file 7: Supplementary Figure S7.Establishment of the xenografted lung cancer model in situ. A: Representative image of position of injection in the mouse model for establishing lung cancer xenograft. B: Representative images of the xenografted lung cancer model in situ. [file 13046_2021_2009_MOESM7_ESM.png]
