# Supplementary material for: Nuclear Transglutaminase 2 interacts with topoisomerase II⍺ to promote DNA damage repair in lung cancer cells
Source: J Exp Clin Cancer Res. 2021 Jul 5;40:224. doi: 10.1186/s13046-021-02009-2 (PMC8258933; doi:10.1186/s13046-021-02009-2)
Supplement: Supplementary file 10 — Additional file 10. [file 13046_2021_2009_MOESM10_ESM.pdf]

Table S3. Sequencing data of TGM2 overexpressing fragments and mutations	
TG2 wild type	atggccgaggagctggtcttagagaggtgtgatctggagctggagaccaatggccgagaccaccacacggcc<br>gacctgtgccgggagaagctggtggtgcgacggggccagcccttctggctgaccctgcactttgagggccgca<br>actacgaggccagtgtagacagtctcaccttcagtgtcgtgaccggcccagcccctagccaggaggccgggacc<br>aaggcccgttttccactaagagatgctgtggaggagggtgactggacagccaccgtggtggaccagcaagact<br>gcaccctctcgctgcagctcaccaccccggccaacgcccccatcggcctgtatcgcctcagcctggaggcctccac<br>tggctaccagggatccagctttgtgctgggccacttcattttgctcttcaacgcctggtgcccagcggatgctgtgta<br>cctggactcggaagaggagcggcaggagtatgtcctcacccagcagggctttatctaccagggctcggccaagt<br>tcatcaagaacataccttggaattttgggcagtttgaagatgggatcctagacatctgcctgatccttctagatgtca<br>accccaagttcctgaagaacgccggccgtgactgctcccgccgcagcagccccgtctacgtgggccgggtggtg<br>agtggcatggtcaactgcaacgatgaccagggtgtgctgctgggacgctgggacaacaactacggggacggc<br>gtcagccccatgtcctggatcggcagcgtggacatcctgcggcgctggaagaaccacggctgccagcgcgtca<br>agtatggccagtgctgggtcttcgccgccgtggcctgcacagtgctgaggtgcctgggcatccctacccgcgtcg<br>tgaccaactacaactcggcccatgaccagaacagcaaccttctcatcgagtacttccgcaatgagtttggggagat<br>ccagggtgacaagagcgagatgatctggaacttccactgctgggtggagtcgtggatgaccaggccggacctg<br>cagccggggtacgagggctggcaggccctggacccaacgccccaggagaagagcgaagggacgtactgctg<br>tggcccagttccagttcgtgccatcaaggagggcgacctgagcaccaagtacgatgcgccctttgtctttgcgga<br>ggtcaatgccgacgtggtagactggatccagcaggacgatgggtctgtgcacaaatccatcaaccgttccctgat
TGM2 ABC	atggccgaggagctggtcttagagaggtgtgatctggagctggagaccaatggccgagaccaccacacggcc<br>gacctgtgccgggagaagctggtggtgcgacggggccagcccttctggctgaccctgcactttgagggccgca<br>actacgaggccagtgtagacagtctcaccttcagtgtcgtgaccggcccagcccctagccaggaggccgggacc<br>aaggcccgttttccactaagagatgctgtggaggagggtgactggacagccaccgtggtggaccagcaagact<br>gcaccctctcgctgcagctcaccaccccggccaacgcccccatcggcctgtatcgcctcagcctggaggcctccac<br>tggctaccagggatccagctttgtgctgggccacttcattttgctcttcaacgcctggtgcccagcggatgctgtgta<br>cctggactcggaagaggagcggcaggagtatgtcctcacccagcagggctttatctaccagggctcggccaagt<br>tcatcaagaacataccttggaattttgggcagtttgaagatgggatcctagacatctgcctgatccttctagatgtca<br>accccaagttcctgaagaacgccggccgtgactgctcccgccgcagcagccccgtctacgtgggccgggtggtg<br>agtggcatggtcaactgcaacgatgaccagggtgtgctgctgggacgctgggacaacaactacggggacggc<br>gtcagccccatgtcctggatcggcagcgtggacatcctgcggcgctggaagaaccacggctgccagcgcgtca<br>agtatggccagtgctgggtcttcgccgccgtggcctgcacagtgctgaggtgcctgggcatccctacccgcgtcg<br>tgaccaactacaactcggcccatgaccagaacagcaaccttctcatcgagtacttccgcaatgagtttggggagat<br>ccagggtgacaagagcgagatgatctggaacttccactgctgggtggagtcgtggatgaccaggccggacctg
TGM2 B+C	ATGgcggatgctgtgtacctggactcggaagaggagcggcaggagtatgtcctcacccagcagggctttatct<br>accagggctcggccaagttcatcaagaacataccttggaattttgggcagtttgaagatgggatcctagacatctg<br>cctgatccttctagatgtcaaccccaagttcctgaagaacgccggccgtgactgctcccgccgcagcagccccgtc<br>tacgtgggccgggtggtgagtggcatggtcaactgcaacgatgaccagggtgtgctgctgggacgctgggac<br>aacaactacggggacggcgtcagccccatgtcctggatcggcagcgtggacatcctgcggcgctggaagaacc<br>acggctgccagcgcgtcaagtatggccagtgctgggtcttcgccgccgtggcctgcacagtgctgaggtgcctg<br>ggcatccctacccgcgtcgtgaccaactacaactcggcccatgaccagaacagcaaccttctcatcgagtacttcc<br>gcaatgagtttggggagatccagggtgacaagagcgagatgatctggaacttccactgctgggtggagtcgtg
TGM2 CD	ATGgccgagaaggaggagacagggatggccatgcggatccgtgtgggccagagcatgaacatgggcagtg<br>actttgacgtctttgcccacatcaccaacaacaccgctgaggagtacgtctgccgcctcctgctctgtgcccgcacc<br>gtcagctacaatgggatcttggggcccgagtgtggcaccaagtacctgctcaacctcaacctggagcctttctctg
TGM2 AB	atggccgaggagctggtcttagagaggtgtgatctggagctggagaccaatggccgagaccaccacacggcc<br>gacctgtgccgggagaagctggtggtgcgacggggccagcccttctggctgaccctgcactttgagggccgca<br>actacgaggccagtgtagacagtctcaccttcagtgtcgtgaccggcccagcccctagccaggaggccgggacc<br>aaggcccgttttccactaagagatgctgtggaggagggtgactggacagccaccgtggtggaccagcaagact<br>gcaccctctcgctgcagctcaccaccccggccaacgcccccatcggcctgtatcgcctcagcctggaggcctccac<br>tggctaccagggatccagctttgtgctgggccacttcattttgctcttcaacgcctggtgcccagcggatgctgtgta<br>cctggactcggaagaggagcggcaggagtatgtcctcacccagcagggctttatctaccagggctcggccaagt<br>tcatcaagaacataccttggaattttgggcagtttgaagatgggatcctagacatctgcctgatccttctagatgtca<br>accccaagttcctgaagaacgccggccgtgactgctcccgccgcagcagccccgtctacgtgggccgggtggtg<br>agtggcatggtcaactgcaacgatgaccagggtgtgctgctgggacgctgggacaacaactacggggacggc<br>gtcagccccatgtcctggatcggcagcgtggacatcctgcggcgctggaagaaccacggctgccagcgcgtca

TGM2 AB+C	atggccgaggagctggtcttagagaggtgtgatctggagctggagaccaatggccgagaccaccacacggcc<br>gacctgtgccgggagaagctggtggtgcgacggggccagcccttctggctgaccctgcactttgagggccgca<br>actacgaggccagtgtagacagtctcaccttcagtgtcgtgaccggcccagcccctagccaggaggccgggacc<br>aaggcccgttttccactaagagatgctgtggaggagggtgactggacagccaccgtggtggaccagcaagact<br>gcaccctctcgctgcagctcaccaccccggccaacgcccccatcggcctgtatcgcctcagcctggaggcctccac<br>tggctaccagggatccagctttgtgctgggccacttcattttgctcttcaacgcctggtgcccagcggatgctgtgta<br>cctggactcggaagaggagcggcaggagtatgtcctcacccagcagggctttatctaccagggctcggccaagt<br>tcatcaagaacataccttggaattttgggcagtttgaagatgggatcctagacatctgcctgatccttctagatgtca<br>accccaagttcctgaagaacgccggccgtgactgctcccgccgcagcagccccgtctacgtgggccgggtggtg<br>agtggcatggtcaactgcaacgatgaccagggtgtgctgctgggacgctgggacaacaactacggggacggc<br>gtcagccccatgtcctggatcggcagcgtggacatcctgcggcgctggaagaaccacggctgccagcgcgtca<br>agtatggccagtgctgggtcttcgccgccgtggcctgcacagtgctgaggtgcctgggcatccctacccgcgtcg
TGM2 C227S	atggccgaggagctggtcttagagaggtgtgatctggagctggagaccaatggccgagaccaccacacggcc<br>gacctgtgccgggagaagctggtggtgcgacggggccagcccttctggctgaccctgcactttgagggccgca<br>actacgaggccagtgtagacagtctcaccttcagtgtcgtgaccggcccagcccctagccaggaggccgggacc<br>aaggcccgttttccactaagagatgctgtggaggagggtgactggacagccaccgtggtggaccagcaagact<br>gcaccctctcgctgcagctcaccaccccggccaacgcccccatcggcctgtatcgcctcagcctggaggcctccac<br>tggctaccagggatccagctttgtgctgggccacttcattttgctcttcaacgcctggtgcccagcggatgctgtgta<br>cctggactcggaagaggagcggcaggagtatgtcctcacccagcagggctttatctaccagggctcggccaagt<br>tcatcaagaacataccttggaattttgggcagtttgaagatgggatcctagacatctgcctgatccttctagatgtca<br>accccaagttcctgaagaacgccggccgtgactgctcccgccgcagcagccccgtctacgtgggccgggtggtg<br>agtggcatggtcaactgcaacgatgaccagggtgtgctgctgggacgctgggacaacaactacggggacggc<br>gtcagccccatgtcctggatcggcagcgtggacatcctgcggcgctggaagaaccacggctgccagcgcgtca<br>agtatggccagt**C**ctgggtcttcgccgccgtggcctgcacagtgctgaggtgcctgggcatccctacccgcgtcg<br>tgaccaactacaactcggcccatgaccagaacagcaaccttctcatcgagtacttccgcaatgagtttggggagat<br>ccagggtgacaagagcgagatgatctggaacttccactgctgggtggagtcgtggatgaccaggccggacctg<br>cagccggggtacgagggctggcaggccctggacccaacgccccaggagaagagcgaagggacgtactgctg<br>tggcccagttccagttcgtgccatcaaggagggcgacctgagcaccaagtacgatgcgccctttgtctttgcgga<br>ggtcaatgccgacgtggtagactggatccagcaggacgatgggtctgtgcacaaatccatcaaccgttccctgat
TGM2 W241A	atggccgaggagctggtcttagagaggtgtgatctggagctggagaccaatggccgagaccaccacacggcc<br>gacctgtgccgggagaagctggtggtgcgacggggccagcccttctggctgaccctgcactttgagggccgca<br>actacgaggccagtgtagacagtctcaccttcagtgtcgtgaccggcccagcccctagccaggaggccgggacc<br>aaggcccgttttccactaagagatgctgtggaggagggtgactggacagccaccgtggtggaccagcaagact<br>gcaccctctcgctgcagctcaccaccccggccaacgcccccatcggcctgtatcgcctcagcctggaggcctccac<br>tggctaccagggatccagctttgtgctgggccacttcattttgctcttcaacgcctggtgcccagcggatgctgtgta<br>cctggactcggaagaggagcggcaggagtatgtcctcacccagcagggctttatctaccagggctcggccaagt<br>tcatcaagaacataccttggaattttgggcagtttgaagatgggatcctagacatctgcctgatccttctagatgtca<br>accccaagttcctgaagaacgccggccgtgactgctcccgccgcagcagccccgtctacgtgggccgggtggtg<br>agtggcatggtcaactgcaacgatgaccagggtgtgctgctgggacgc**GC**ggacaacaactacggggacgg<br>cgtcagccccatgtcctggatcggcagcgtggacatcctgcggcgctggaagaaccacggctgccagcgcgtc<br>aagtatggccagtgctgggtcttcgccgccgtggcctgcacagtgctgaggtgcctgggcatccctacccgcgtc<br>gtgaccaactacaactcggcccatgaccagaacagcaaccttctcatcgagtacttccgcaatgagtttggggaga<br>tccagggtgacaagagcgagatgatctggaacttccactgctgggtggagtcgtggatgaccaggccggacct<br>gcagccggggtacgagggctggcaggccctggacccaacgccccaggagaagagcgaagggacgtactgct<br>gtggcccagttccagttcgtgccatcaaggagggcgacctgagcaccaagtacgatgcgccctttgtctttgcgg<br>aggtcaatgccgacgtggtagactggatccagcaggacgatgggtctgtgcacaaatccatcaaccgttccctga

R580A	atggccgaggagctggtcttagagaggtgtgatctggagctggagaccaatggccgagaccaccacacggcc<br>gacctgtgccgggagaagctggtggtgcgacggggccagcccttctggctgaccctgcactttgagggccgca<br>actacgaggccagtgtagacagtctcaccttcagtgtcgtgaccggcccagcccctagccaggaggccgggacc<br>aaggcccgttttccactaagagatgctgtggaggagggtgactggacagccaccgtggtggaccagcaagact<br>gcaccctctcgctgcagctcaccaccccggccaacgcccccatcggcctgtatcgcctcagcctggaggcctccac<br>tggctaccagggatccagctttgtgctgggccacttcattttgctcttcaacgcctggtgcccagcggatgctgtgta<br>cctggactcggaagaggagcggcaggagtatgtcctcacccagcagggctttatctaccagggctcggccaagt<br>tcatcaagaacataccttggaattttgggcagtttgaagatgggatcctagacatctgcctgatccttctagatgtca<br>accccaagttcctgaagaacgccggccgtgactgctcccgccgcagcagccccgtctacgtgggccgggtggtg<br>agtggcatggtcaactgcaacgatgaccagggtgtgctgctgggacgctgggacaacaactacggggacggc<br>gtcagccccatgtcctggatcggcagcgtggacatcctgcggcgctggaagaaccacggctgccagcgcgtca<br>agtatggccagtgctgggtcttcgccgccgtggcctgcacagtgctgaggtgcctgggcatccctacccgcgtcg<br>tgaccaactacaactcggcccatgaccagaacagcaaccttctcatcgagtacttccgcaatgagtttggggagat<br>ccagggtgacaagagcgagatgatctggaacttccactgctgggtggagtcgtggatgaccaggccggacctg<br>cagccggggtacgagggctggcaggccctggacccaacgccccaggagaagagcgaagggacgtactgctg<br>tggcccagttccagttcgtgccatcaaggagggcgacctgagcaccaagtacgatgcgccctttgtctttgcgga<br>ggtcaatgccgacgtggtagactggatccagcaggacgatgggtctgtgcacaaatccatcaaccgttccctgat
Y516F	atggccgaggagctggtcttagagaggtgtgatctggagctggagaccaatggccgagaccaccacacggcc<br>gacctgtgccgggagaagctggtggtgcgacggggccagcccttctggctgaccctgcactttgagggccgca<br>actacgaggccagtgtagacagtctcaccttcagtgtcgtgaccggcccagcccctagccaggaggccgggacc<br>aaggcccgttttccactaagagatgctgtggaggagggtgactggacagccaccgtggtggaccagcaagact<br>gcaccctctcgctgcagctcaccaccccggccaacgcccccatcggcctgtatcgcctcagcctggaggcctccac<br>tggctaccagggatccagctttgtgctgggccacttcattttgctcttcaacgcctggtgcccagcggatgctgtgta<br>cctggactcggaagaggagcggcaggagtatgtcctcacccagcagggctttatctaccagggctcggccaagt<br>tcatcaagaacataccttggaattttgggcagtttgaagatgggatcctagacatctgcctgatccttctagatgtca<br>accccaagttcctgaagaacgccggccgtgactgctcccgccgcagcagccccgtctacgtgggccgggtggtg<br>agtggcatggtcaactgcaacgatgaccagggtgtgctgctgggacgctgggacaacaactacggggacggc<br>gtcagccccatgtcctggatcggcagcgtggacatcctgcggcgctggaagaaccacggctgccagcgcgtca<br>agtatggccagtgctgggtcttcgccgccgtggcctgcacagtgctgaggtgcctgggcatccctacccgcgtcg<br>tgaccaactacaactcggcccatgaccagaacagcaaccttctcatcgagtacttccgcaatgagtttggggagat<br>ccagggtgacaagagcgagatgatctggaacttccactgctgggtggagtcgtggatgaccaggccggacctg<br>cagccggggtacgagggctggcaggccctggacccaacgccccaggagaagagcgaagggacgtactgctg<br>tggcccagttccagttcgtgccatcaaggagggcgacctgagcaccaagtacgatgcgccctttgtctttgcgga<br>ggtcaatgccgacgtggtagactggatccagcaggacgatgggtctgtgcacaaatccatcaaccgttccctgat
TG2 S68A	TGAACCGTCAGATCGCCTGGAGACGCCATCCACGCTGTTTTGACCTCCATAGAAGATT<br>CTAGAGCTAGCGAATTCATGGCCGAGGAGCTGGTCTTAGAGAGGTGTGATCTGGAGC<br>TGGAGACCAATGGCCGAGACCACCACACGGCCGACCTGTGCCGGGAGAAGCTGGTG<br>GTGCGACGGGGCCAGCCCTTCTGGCTGACCCTGCACTTTGAGGGCCGCAACTACGAG<br>GCCAGTGTAGACAGTCTCACCTTCAGTGTCGTGACCGGCCCAGCCCCTGCCCAGGAG<br>GCCGGGACCAAGGCCCGTTTTCCACTAAGAGATGCTGTGGAGGAGGGTGACTGGAC<br>AGCCACCGTGGTGGACCAGCAAGACTGCACCCTCTCGCTGCAGCTCACCACCCCGG<br>CCAACGCCCCCATCGGCCTGTATCGCCTCAGCCTGGAGGCCTCCACTGGCTACCAGG<br>GATCCAGCTTTGTGCTGGGCCACTTCATTTTGCTCTTCAACGCCTGGTGCCCAGCGGA<br>TGCTGTGTACCTGGACTCGGAAGAGGAGCGGCAGGAGTATGTCCTCACCCAGCAGG<br>GCTTTATCTACCAGGGCTCGGCCAAGTTCATCAAGAACATACCTTGGAATTTTGGGCA<br>GTTTGAAGATGGGATCCTAGACATCTGCCTGATCCTTCTAGATGTCAACCCCAAGTTC<br>CTGAAGAACGCCGGCCGTGACTGCTCCCGCCGCAGCAGCCCCGTCTACGTGGGCCG<br>GGTGGTGAGTGGCATGGTCAACTGCAACGATGACCAGGGTGTGCTGCTGGGACGCT<br>GGGACAACAACTACGGGGACGGCGTCAGCCCCATGTCCTGGATCGGCAGCGTGGAC<br>ATCCTGCGGCGCTGGAAGAACCACGGCTGCCAGCGCGTCAAGTATGGCCAGTGCTG<br>GGTCTTCGCCGCCGTGGCCTGCACAGTGCTGAGGTGCCTAGGCATCCCTACCCGCGT<br>CGTGACCAACTACAACTCGGCCCATGACCAGAACAGCAACCTTCTCATCGAGTACTT<br>CCGCAATGAGTTTGGGGAGATCCAGGGTGACAAGAGCGAGATGATCTGGAACTTCC<br>ACTGCTGGGTGGAGTCGTGGATGACCAGGCCGGACCTGCAGCCGGGGTACGAGGGC<br>TGGCAGGCCCTGGACCCAACGCCCCAGGAGAAGAGCGAAGGGACGTACTGCTGTGG<br>CCCAGTTCCAGTTCGTGCCATCAAGGAGGGCGACCTGAGCACCAAGTACGATGCGCC<br>CTTTGTCTTTGCGGAGGTCAATGCCGACGTGGTAGACTGGATCCAGCAGGACGATGG<br>GTCTGTGCACAAATCCATCAACCGTTCCCTGATCGTTGGGCTGAAGATCAGCACTAAG

TG2 T162A	CCACGCTGTTTTGACCTCCATAGAAGATTCTAGAGCTAGCGAATTCATGGCCGAGGA<br>GCTGGTCTTAGAGAGGTGTGATCTGGAGCTGGAGACCAATGGCCGAGACCACCACA<br>CGGCCGACCTGTGCCGGGAGAAGCTGGTGGTGCGACGGGGCCAGCCCTTCTGGCTG<br>ACCCTGCACTTTGAGGGCCGCAACTACGAGGCCAGTGTAGACAGTCTCACCTTCAGT<br>GTCGTGACCGGCCCAGCCCCTAGCCAGGAGGCCGGGACCAAGGCCCGTTTTCCACT<br>AAGAGATGCTGTGGAGGAGGGTGACTGGACAGCCACCGTGGTGGACCAGCAAGACT<br>GCACCCTCTCGCTGCAGCTCACCACCCCGGCCAACGCCCCCATCGGCCTGTATCGCC<br>TCAGCCTGGAGGCCTCCACTGGCTACCAGGGATCCAGCTTTGTGCTGGGCCACTTCA<br>TTTTGCTCTTCAACGCCTGGTGCCCAGCGGATGCTGTGTACCTGGACTCGGAAGAGG<br>AGCGGCAGGAGTATGTCCTCGCCCAGCAGGGCTTTATCTACCAGGGCTCGGCCAAGT<br>TCATCAAGAACATACCTTGGAATTTTGGGCAGTTTGAAGATGGGATCCTAGACATCTG<br>CCTGATCCTTCTAGATGTCAACCCCAAGTTCCTGAAGAACGCCGGCCGTGACTGCTCC<br>CGCCGCAGCAGCCCCGTCTACGTGGGCCGGGTGGTGAGTGGCATGGTCAACTGCAA<br>CGATGACCAGGGTGTGCTGCTGGGACGCTGGGACAACAACTACGGGGACGGCGTCA<br>GCCCCATGTCCTGGATCGGCAGCGTGGACATCCTGCGGCGCTGGAAGAACCACGGC<br>TGCCAGCGCGTCAAGTATGGCCAGTGCTGGGTCTTCGCCGCCGTGGCCTGCACAGTG<br>CTGAGGTGCCTAGGCATCCCTACCCGCGTCGTGACCAACTACAACTCGGCCCATGAC<br>CAGAACAGCAACCTTCTCATCGAGTACTTCCGCAATGAGTTTGGGGAGATCCAGGGT<br>GACAAGAGCGAGATGATCTGGAACTTCCACTGCTGGGTGGAGTCGTGGATGACCAG<br>GCCGGACCTGCAGCCGGGGTACGAGGGCTGGCAGGCCCTGGACCCAACGCCCCAG<br>GAGAAGAGCGAAGGGACGTACTGCTGTGGCCCAGTTCCAGTTCGTGCCATCAAGGA<br>GGGCGACCTGAGCACCAAGTACGATGCGCCCTTTGTCTTTGCGGAGGTCAATGCCGA<br>CGTGGTAGACTGGATCCAGCAGGACGATGGGTCTGTGCACAAATCCATCAACCGTTC<br>CCTGATCGTTGGGCTGAAGATCAGCACTAAGAGCGTGGGCCGAGACGAGCGGGAGG
TG2 S68A+T162A	GTGaACCGTCAGATCGCCTGGAGACGCCATCCACGCTGTTTTGACCTCCATAGAAGAT<br>TCTAGAGCTAGCGAATTCATGGCCGAGGAGCTGGTCTTAGAGAGGTGTGATCTGGAG<br>CTGGAGACCAATGGCCGAGACCACCACACGGCCGACCTGTGCCGGGAGAAGCTGGT<br>GGTGCGACGGGGCCAGCCCTTCTGGCTGACCCTGCACTTTGAGGGCCGCAACTACGA<br>GGCCAGTGTAGACAGTCTCACCTTCAGTGTCGTGACCGGCCCAGCCCCTGCCCAGGA<br>GGCCGGGACCAAGGCCCGTTTTCCACTAAGAGATGCTGTGGAGGAGGGTGACTGGA<br>CAGCCACCGTGGTGGACCAGCAAGACTGCACCCTCTCGCTGCAGCTCACCACCCCG<br>GCCAACGCCCCCATCGGCCTGTATCGCCTCAGCCTGGAGGCCTCCACTGGCTACCAG<br>GGATCCAGCTTTGTGCTGGGCCACTTCATTTTGCTCTTCAACGCCTGGTGCCCAGCGG<br>ATGCTGTGTACCTGGACTCGGAAGAGGAGCGGCAGGAGTATGTCCTCGCCCAGCAG<br>GGCTTTATCTACCAGGGCTCGGCCAAGTTCATCAAGAACATACCTTGGAATTTTGGGC<br>AGTTTGAAGATGGGATCCTAGACATCTGCCTGATCCTTCTAGATGTCAACCCCAAGTT<br>CCTGAAGAACGCCGGCCGTGACTGCTCCCGCCGCAGCAGCCCCGTCTACGTGGGCC<br>GGGTGGTGAGTGGCATGGTCAACTGCAACGATGACCAGGGTGTGCTGCTGGGACGC<br>TGGGACAACAACTACGGGGACGGCGTCAGCCCCATGTCCTGGATCGGCAGCGTGGA<br>CATCCTGCGGCGCTGGAAGAACCACGGCTGCCAGCGCGTCAAGTATGGCCAGTGCT<br>GGGTCTTCGCCGCCGTGGCCTGCACAGTGCTGAGGTGCCTAGGCATCCCTACCCGCG<br>TCGTGACCAACTACAACTCGGCCCATGACCAGAACAGCAACCTTCTCATCGAGTACT<br>TCCGCAATGAGTTTGGGGAGATCCAGGGTGACAAGAGCGAGATGATCTGGAACTTCC<br>ACTGCTGGGTGGAGTCGTGGATGACCAGGCCGGACCTGCAGCCGGGGTACGAGGGC<br>TGGCAGGCCCTGGACCCAACGCCCCAGGAGAAGAGCGAAGGGACGTACTGCTGTGG<br>CCCAGTTCCAGTTCGTGCCATCAAGGAGGGCGACCTGAGCACCAAGTACGATGCGCC<br>CTTTGTCTTTGCGGAGGTCAATGCCGACGTGGTAGACTGGATCCAGCAGGACGATGG
