# Supplementary material for: Nuclear Transglutaminase 2 interacts with topoisomerase II⍺ to promote DNA damage repair in lung cancer cells
Source: J Exp Clin Cancer Res. 2021 Jul 5;40:224. doi: 10.1186/s13046-021-02009-2 (PMC8258933; doi:10.1186/s13046-021-02009-2)
Supplement: Supplementary file 11 — Additional file 11. [file 13046_2021_2009_MOESM11_ESM.pdf]

# NetPhos 3.1 Server - prediction results

## Technical University of Denmark

---

```
>AAH03551.1      548 amino acids
#
# netphos-3.1b prediction results
#
# Sequence                   # x   Context     Score   Kinase    Answer
# -------------------------------------------------------------------
# AAH03551.1                16 T   LELETNGRD   0.486   CKII        .
# AAH03551.1                16 T   LELETNGRD   0.461   GSK3        .
# AAH03551.1                16 T   LELETNGRD   0.432   CaM-II      .
# AAH03551.1                16 T   LELETNGRD   0.411   cdc2        .
# AAH03551.1                16 T   LELETNGRD   0.367   CKI         .
# AAH03551.1                16 T   LELETNGRD   0.342   DNAPK       .
# AAH03551.1                16 T   LELETNGRD   0.302   p38MAPK     .
# AAH03551.1                16 T   LELETNGRD   0.267   PKC         .
# AAH03551.1                16 T   LELETNGRD   0.240   ATM         .
# AAH03551.1                16 T   LELETNGRD   0.237   PKG         .
# AAH03551.1                16 T   LELETNGRD   0.192   RSK         .
# AAH03551.1                16 T   LELETNGRD   0.171   cdk5        .
# AAH03551.1                16 T   LELETNGRD   0.088   PKA         .
# AAH03551.1                16 T   LELETNGRD   0.085   PKB         .
# AAH03551.1                16 T   LELETNGRD   0.029   unsp        .
#
# AAH03551.1                23 T   RDHHTADLC   0.568   CKII       YES
# AAH03551.1                23 T   RDHHTADLC   0.452   CaM-II      .
# AAH03551.1                23 T   RDHHTADLC   0.448   GSK3        .
# AAH03551.1                23 T   RDHHTADLC   0.388   cdc2        .
# AAH03551.1                23 T   RDHHTADLC   0.361   CKI         .
# AAH03551.1                23 T   RDHHTADLC   0.343   PKG         .
# AAH03551.1                23 T   RDHHTADLC   0.342   DNAPK       .
# AAH03551.1                23 T   RDHHTADLC   0.325   p38MAPK     .
# AAH03551.1                23 T   RDHHTADLC   0.260   ATM         .
# AAH03551.1                23 T   RDHHTADLC   0.224   PKA         .
# AAH03551.1                23 T   RDHHTADLC   0.201   RSK         .
# AAH03551.1                23 T   RDHHTADLC   0.153   cdk5        .
# AAH03551.1                23 T   RDHHTADLC   0.090   PKC         .
# AAH03551.1                23 T   RDHHTADLC   0.087   PKB         .
# AAH03551.1                23 T   RDHHTADLC   0.029   unsp        .
#
# AAH03551.1                42 T   PFWLTLHFE   0.513   PKC        YES
# AAH03551.1                42 T   PFWLTLHFE   0.493   cdc2        .
# AAH03551.1                42 T   PFWLTLHFE   0.442   CaM-II      .
# AAH03551.1                42 T   PFWLTLHFE   0.432   GSK3        .
# AAH03551.1                42 T   PFWLTLHFE   0.391   DNAPK       .
# AAH03551.1                42 T   PFWLTLHFE   0.359   PKG         .
# AAH03551.1                42 T   PFWLTLHFE   0.359   CKI         .
# AAH03551.1                42 T   PFWLTLHFE   0.324   CKII        .
# AAH03551.1                42 T   PFWLTLHFE   0.315   p38MAPK     .
# AAH03551.1                42 T   PFWLTLHFE   0.260   RSK         .
# AAH03551.1                42 T   PFWLTLHFE   0.231   ATM         .
# AAH03551.1                42 T   PFWLTLHFE   0.188   cdk5        .
# AAH03551.1                42 T   PFWLTLHFE   0.177   PKA         .
# AAH03551.1                42 T   PFWLTLHFE   0.145   unsp        .
# AAH03551.1                42 T   PFWLTLHFE   0.101   PKB         .
#
# AAH03551.1                50 Y   EGRNYEASV   0.480   SRC         .
# AAH03551.1                50 Y   EGRNYEASV   0.420   INSR        .
# AAH03551.1                50 Y   EGRNYEASV   0.336   EGFR        .
# AAH03551.1                50 Y   EGRNYEASV   0.178   unsp        .
```

```
#
# AAH03551.1            53 S   NYEASVDSL   0.471   CaM-II     .
# AAH03551.1            53 S   NYEASVDSL   0.461   PKA        .
# AAH03551.1            53 S   NYEASVDSL   0.436   GSK3       .
# AAH03551.1            53 S   NYEASVDSL   0.423   cdc2       .
# AAH03551.1            53 S   NYEASVDSL   0.386   CKII       .
# AAH03551.1            53 S   NYEASVDSL   0.383   RSK        .
# AAH03551.1            53 S   NYEASVDSL   0.365   CKI        .
# AAH03551.1            53 S   NYEASVDSL   0.360   DNAPK      .
# AAH03551.1            53 S   NYEASVDSL   0.349   p38MAPK    .
# AAH03551.1            53 S   NYEASVDSL   0.326   PKC        .
# AAH03551.1            53 S   NYEASVDSL   0.287   ATM        .
# AAH03551.1            53 S   NYEASVDSL   0.198   PKG        .
# AAH03551.1            53 S   NYEASVDSL   0.188   PKB        .
# AAH03551.1            53 S   NYEASVDSL   0.170   cdk5       .
# AAH03551.1            53 S   NYEASVDSL   0.162   unsp       .
#
# AAH03551.1            56 S   ASVDSLTFS   0.558   unsp      YES
# AAH03551.1            56 S   ASVDSLTFS   0.497   cdc2       .
# AAH03551.1            56 S   ASVDSLTFS   0.487   CKI        .
# AAH03551.1            56 S   ASVDSLTFS   0.475   GSK3       .
# AAH03551.1            56 S   ASVDSLTFS   0.434   CaM-II     .
# AAH03551.1            56 S   ASVDSLTFS   0.391   DNAPK      .
# AAH03551.1            56 S   ASVDSLTFS   0.358   p38MAPK    .
# AAH03551.1            56 S   ASVDSLTFS   0.311   CKII       .
# AAH03551.1            56 S   ASVDSLTFS   0.298   RSK        .
# AAH03551.1            56 S   ASVDSLTFS   0.289   ATM        .
# AAH03551.1            56 S   ASVDSLTFS   0.280   PKA        .
# AAH03551.1            56 S   ASVDSLTFS   0.260   PKG        .
# AAH03551.1            56 S   ASVDSLTFS   0.219   cdk5       .
# AAH03551.1            56 S   ASVDSLTFS   0.095   PKC        .
# AAH03551.1            56 S   ASVDSLTFS   0.090   PKB        .
#
# AAH03551.1            58 T   VDSLTFSVV   0.619   PKC       YES
# AAH03551.1            58 T   VDSLTFSVV   0.431   cdc2       .
# AAH03551.1            58 T   VDSLTFSVV   0.430   CKI        .
# AAH03551.1            58 T   VDSLTFSVV   0.429   GSK3       .
# AAH03551.1            58 T   VDSLTFSVV   0.407   CaM-II     .
# AAH03551.1            58 T   VDSLTFSVV   0.384   p38MAPK    .
# AAH03551.1            58 T   VDSLTFSVV   0.349   DNAPK      .
# AAH03551.1            58 T   VDSLTFSVV   0.348   CKII       .
# AAH03551.1            58 T   VDSLTFSVV   0.263   ATM        .
# AAH03551.1            58 T   VDSLTFSVV   0.241   PKG        .
# AAH03551.1            58 T   VDSLTFSVV   0.236   RSK        .
# AAH03551.1            58 T   VDSLTFSVV   0.168   cdk5       .
# AAH03551.1            58 T   VDSLTFSVV   0.130   PKA        .
# AAH03551.1            58 T   VDSLTFSVV   0.082   PKB        .
# AAH03551.1            58 T   VDSLTFSVV   0.050   unsp       .
#
# AAH03551.1            60 S   SLTFSVVTG   0.471   CaM-II     .
# AAH03551.1            60 S   SLTFSVVTG   0.464   GSK3       .
# AAH03551.1            60 S   SLTFSVVTG   0.446   cdc2       .
# AAH03551.1            60 S   SLTFSVVTG   0.382   CKI        .
# AAH03551.1            60 S   SLTFSVVTG   0.354   cdk5       .
# AAH03551.1            60 S   SLTFSVVTG   0.354   unsp       .
# AAH03551.1            60 S   SLTFSVVTG   0.348   DNAPK      .
# AAH03551.1            60 S   SLTFSVVTG   0.341   ATM        .
# AAH03551.1            60 S   SLTFSVVTG   0.315   CKII       .
# AAH03551.1            60 S   SLTFSVVTG   0.304   p38MAPK    .
# AAH03551.1            60 S   SLTFSVVTG   0.275   RSK        .
# AAH03551.1            60 S   SLTFSVVTG   0.244   PKC        .
# AAH03551.1            60 S   SLTFSVVTG   0.239   PKG        .
# AAH03551.1            60 S   SLTFSVVTG   0.204   PKA        .
# AAH03551.1            60 S   SLTFSVVTG   0.080   PKB        .
#
# AAH03551.1            63 T   FSVVTGPAP   0.640   PKC       YES
# AAH03551.1            63 T   FSVVTGPAP   0.459   CaM-II     .
# AAH03551.1            63 T   FSVVTGPAP   0.434   GSK3       .
# AAH03551.1            63 T   FSVVTGPAP   0.377   CKI        .
```

```
# AAH03551.1          63 T   FSVVTGPAP   0.361   cdc2        .
# AAH03551.1          63 T   FSVVTGPAP   0.342   DNAPK       .
# AAH03551.1          63 T   FSVVTGPAP   0.310   CKII        .
# AAH03551.1          63 T   FSVVTGPAP   0.299   p38MAPK     .
# AAH03551.1          63 T   FSVVTGPAP   0.274   cdk5        .
# AAH03551.1          63 T   FSVVTGPAP   0.262   ATM         .
# AAH03551.1          63 T   FSVVTGPAP   0.238   PKG         .
# AAH03551.1          63 T   FSVVTGPAP   0.205   RSK         .
# AAH03551.1          63 T   FSVVTGPAP   0.120   PKA         .
# AAH03551.1          63 T   FSVVTGPAP   0.085   PKB         .
# AAH03551.1          63 T   FSVVTGPAP   0.027   unsp        .
#
# AAH03551.1          68 S   GPAPSQEAG   0.990   unsp        YES
# AAH03551.1          68 S   GPAPSQEAG   0.607   DNAPK       YES
# AAH03551.1          68 S   GPAPSQEAG   0.578   ATM         YES
# AAH03551.1          68 S   GPAPSQEAG   0.514   CKI         YES
# AAH03551.1          68 S   GPAPSQEAG   0.458   GSK3        .
# AAH03551.1          68 S   GPAPSQEAG   0.438   CaM-II      .
# AAH03551.1          68 S   GPAPSQEAG   0.389   cdc2        .
# AAH03551.1          68 S   GPAPSQEAG   0.363   CKII        .
# AAH03551.1          68 S   GPAPSQEAG   0.291   p38MAPK     .
# AAH03551.1          68 S   GPAPSQEAG   0.239   RSK         .
# AAH03551.1          68 S   GPAPSQEAG   0.237   PKG         .
# AAH03551.1          68 S   GPAPSQEAG   0.190   cdk5        .
# AAH03551.1          68 S   GPAPSQEAG   0.141   PKA         .
# AAH03551.1          68 S   GPAPSQEAG   0.115   PKC         .
# AAH03551.1          68 S   GPAPSQEAG   0.078   PKB         .
#
# AAH03551.1          73 T   QEAGTKARF   0.840   PKC         YES
# AAH03551.1          73 T   QEAGTKARF   0.437   GSK3        .
# AAH03551.1          73 T   QEAGTKARF   0.426   CaM-II      .
# AAH03551.1          73 T   QEAGTKARF   0.386   cdc2        .
# AAH03551.1          73 T   QEAGTKARF   0.377   p38MAPK     .
# AAH03551.1          73 T   QEAGTKARF   0.367   CKI         .
# AAH03551.1          73 T   QEAGTKARF   0.354   DNAPK       .
# AAH03551.1          73 T   QEAGTKARF   0.346   CKII        .
# AAH03551.1          73 T   QEAGTKARF   0.334   unsp        .
# AAH03551.1          73 T   QEAGTKARF   0.293   PKG         .
# AAH03551.1          73 T   QEAGTKARF   0.241   ATM         .
# AAH03551.1          73 T   QEAGTKARF   0.224   RSK         .
# AAH03551.1          73 T   QEAGTKARF   0.208   cdk5        .
# AAH03551.1          73 T   QEAGTKARF   0.088   PKA         .
# AAH03551.1          73 T   QEAGTKARF   0.079   PKB         .
#
# AAH03551.1          89 T   EGDWTATVV   0.453   GSK3        .
# AAH03551.1          89 T   EGDWTATVV   0.443   CaM-II      .
# AAH03551.1          89 T   EGDWTATVV   0.400   p38MAPK     .
# AAH03551.1          89 T   EGDWTATVV   0.377   CKII        .
# AAH03551.1          89 T   EGDWTATVV   0.369   CKI         .
# AAH03551.1          89 T   EGDWTATVV   0.354   DNAPK       .
# AAH03551.1          89 T   EGDWTATVV   0.342   cdc2        .
# AAH03551.1          89 T   EGDWTATVV   0.328   PKG         .
# AAH03551.1          89 T   EGDWTATVV   0.273   ATM         .
# AAH03551.1          89 T   EGDWTATVV   0.184   RSK         .
# AAH03551.1          89 T   EGDWTATVV   0.174   PKA         .
# AAH03551.1          89 T   EGDWTATVV   0.161   unsp        .
# AAH03551.1          89 T   EGDWTATVV   0.158   cdk5        .
# AAH03551.1          89 T   EGDWTATVV   0.092   PKB         .
# AAH03551.1          89 T   EGDWTATVV   0.065   PKC         .
#
# AAH03551.1          91 T   DWTATVVDQ   0.670   unsp        YES
# AAH03551.1          91 T   DWTATVVDQ   0.485   cdc2        .
# AAH03551.1          91 T   DWTATVVDQ   0.455   CaM-II      .
# AAH03551.1          91 T   DWTATVVDQ   0.413   GSK3        .
# AAH03551.1          91 T   DWTATVVDQ   0.395   CKII        .
# AAH03551.1          91 T   DWTATVVDQ   0.394   PKC         .
# AAH03551.1          91 T   DWTATVVDQ   0.361   CKI         .
# AAH03551.1          91 T   DWTATVVDQ   0.342   DNAPK       .
# AAH03551.1          91 T   DWTATVVDQ   0.317   p38MAPK     .
```

```
# AAH03551.1            91 T   DWTATVVDQ   0.259   RSK         .
# AAH03551.1            91 T   DWTATVVDQ   0.256   ATM         .
# AAH03551.1            91 T   DWTATVVDQ   0.220   PKG         .
# AAH03551.1            91 T   DWTATVVDQ   0.200   cdk5        .
# AAH03551.1            91 T   DWTATVVDQ   0.126   PKA         .
# AAH03551.1            91 T   DWTATVVDQ   0.095   PKB         .
#
# AAH03551.1            99 T   QQDCTLSLQ   0.449   GSK3        .
# AAH03551.1            99 T   QQDCTLSLQ   0.442   cdc2        .
# AAH03551.1            99 T   QQDCTLSLQ   0.428   CaM-II      .
# AAH03551.1            99 T   QQDCTLSLQ   0.419   DNAPK       .
# AAH03551.1            99 T   QQDCTLSLQ   0.366   CKI         .
# AAH03551.1            99 T   QQDCTLSLQ   0.342   CKII        .
# AAH03551.1            99 T   QQDCTLSLQ   0.337   p38MAPK     .
# AAH03551.1            99 T   QQDCTLSLQ   0.286   PKG         .
# AAH03551.1            99 T   QQDCTLSLQ   0.262   PKA         .
# AAH03551.1            99 T   QQDCTLSLQ   0.249   ATM         .
# AAH03551.1            99 T   QQDCTLSLQ   0.238   PKC         .
# AAH03551.1            99 T   QQDCTLSLQ   0.215   RSK         .
# AAH03551.1            99 T   QQDCTLSLQ   0.172   cdk5        .
# AAH03551.1            99 T   QQDCTLSLQ   0.081   PKB         .
# AAH03551.1            99 T   QQDCTLSLQ   0.008   unsp        .
#
# AAH03551.1           101 S   DCTLSLQLT   0.590   PKA         YES
# AAH03551.1           101 S   DCTLSLQLT   0.476   DNAPK       .
# AAH03551.1           101 S   DCTLSLQLT   0.449   CaM-II      .
# AAH03551.1           101 S   DCTLSLQLT   0.434   GSK3        .
# AAH03551.1           101 S   DCTLSLQLT   0.386   cdc2        .
# AAH03551.1           101 S   DCTLSLQLT   0.365   CKI         .
# AAH03551.1           101 S   DCTLSLQLT   0.363   p38MAPK     .
# AAH03551.1           101 S   DCTLSLQLT   0.328   RSK         .
# AAH03551.1           101 S   DCTLSLQLT   0.312   CKII        .
# AAH03551.1           101 S   DCTLSLQLT   0.285   ATM         .
# AAH03551.1           101 S   DCTLSLQLT   0.227   PKG         .
# AAH03551.1           101 S   DCTLSLQLT   0.210   PKC         .
# AAH03551.1           101 S   DCTLSLQLT   0.170   cdk5        .
# AAH03551.1           101 S   DCTLSLQLT   0.087   PKB         .
# AAH03551.1           101 S   DCTLSLQLT   0.005   unsp        .
#
# AAH03551.1           105 T   SLQLTTPAN   0.473   cdc2        .
# AAH03551.1           105 T   SLQLTTPAN   0.461   CaM-II      .
# AAH03551.1           105 T   SLQLTTPAN   0.455   GSK3        .
# AAH03551.1           105 T   SLQLTTPAN   0.388   PKC         .
# AAH03551.1           105 T   SLQLTTPAN   0.373   CKI         .
# AAH03551.1           105 T   SLQLTTPAN   0.345   DNAPK       .
# AAH03551.1           105 T   SLQLTTPAN   0.323   CKII        .
# AAH03551.1           105 T   SLQLTTPAN   0.277   PKG         .
# AAH03551.1           105 T   SLQLTTPAN   0.271   p38MAPK     .
# AAH03551.1           105 T   SLQLTTPAN   0.268   ATM         .
# AAH03551.1           105 T   SLQLTTPAN   0.262   RSK         .
# AAH03551.1           105 T   SLQLTTPAN   0.191   cdk5        .
# AAH03551.1           105 T   SLQLTTPAN   0.091   PKA         .
# AAH03551.1           105 T   SLQLTTPAN   0.085   PKB         .
# AAH03551.1           105 T   SLQLTTPAN   0.031   unsp        .
#
# AAH03551.1           106 T   LQLTTPANA   0.527   p38MAPK     YES
# AAH03551.1           106 T   LQLTTPANA   0.479   GSK3        .
# AAH03551.1           106 T   LQLTTPANA   0.447   CaM-II      .
# AAH03551.1           106 T   LQLTTPANA   0.406   cdk5        .
# AAH03551.1           106 T   LQLTTPANA   0.380   cdc2        .
# AAH03551.1           106 T   LQLTTPANA   0.365   CKI         .
# AAH03551.1           106 T   LQLTTPANA   0.340   DNAPK       .
# AAH03551.1           106 T   LQLTTPANA   0.311   CKII        .
# AAH03551.1           106 T   LQLTTPANA   0.298   PKG         .
# AAH03551.1           106 T   LQLTTPANA   0.252   RSK         .
# AAH03551.1           106 T   LQLTTPANA   0.243   ATM         .
# AAH03551.1           106 T   LQLTTPANA   0.221   unsp        .
# AAH03551.1           106 T   LQLTTPANA   0.117   PKA         .
# AAH03551.1           106 T   LQLTTPANA   0.098   PKC         .
```

```
# AAH03551.1            106 T   LQLTTPANA    0.081    PKB          .
#
# AAH03551.1            115 Y   PIGLYRLSL    0.349    INSR         .
# AAH03551.1            115 Y   PIGLYRLSL    0.329    EGFR         .
# AAH03551.1            115 Y   PIGLYRLSL    0.327    SRC          .
# AAH03551.1            115 Y   PIGLYRLSL    0.010    unsp         .
#
# AAH03551.1            118 S   LYRLSLEAS    0.957    unsp         YES
# AAH03551.1            118 S   LYRLSLEAS    0.740    PKA          YES
# AAH03551.1            118 S   LYRLSLEAS    0.492    DNAPK        .
# AAH03551.1            118 S   LYRLSLEAS    0.466    GSK3         .
# AAH03551.1            118 S   LYRLSLEAS    0.419    CaM-II       .
# AAH03551.1            118 S   LYRLSLEAS    0.417    CKI          .
# AAH03551.1            118 S   LYRLSLEAS    0.394    cdc2         .
# AAH03551.1            118 S   LYRLSLEAS    0.383    PKC          .
# AAH03551.1            118 S   LYRLSLEAS    0.351    ATM          .
# AAH03551.1            118 S   LYRLSLEAS    0.350    RSK          .
# AAH03551.1            118 S   LYRLSLEAS    0.332    CKII         .
# AAH03551.1            118 S   LYRLSLEAS    0.275    p38MAPK      .
# AAH03551.1            118 S   LYRLSLEAS    0.259    PKG          .
# AAH03551.1            118 S   LYRLSLEAS    0.166    cdk5         .
# AAH03551.1            118 S   LYRLSLEAS    0.083    PKB          .
#
# AAH03551.1            122 S   SLEASTGYQ    0.504    cdc2         YES
# AAH03551.1            122 S   SLEASTGYQ    0.477    GSK3         .
# AAH03551.1            122 S   SLEASTGYQ    0.452    CaM-II       .
# AAH03551.1            122 S   SLEASTGYQ    0.400    CKI          .
# AAH03551.1            122 S   SLEASTGYQ    0.356    DNAPK        .
# AAH03551.1            122 S   SLEASTGYQ    0.310    ATM          .
# AAH03551.1            122 S   SLEASTGYQ    0.309    RSK          .
# AAH03551.1            122 S   SLEASTGYQ    0.302    CKII         .
# AAH03551.1            122 S   SLEASTGYQ    0.294    p38MAPK      .
# AAH03551.1            122 S   SLEASTGYQ    0.261    PKG          .
# AAH03551.1            122 S   SLEASTGYQ    0.232    PKC          .
# AAH03551.1            122 S   SLEASTGYQ    0.232    PKA          .
# AAH03551.1            122 S   SLEASTGYQ    0.226    cdk5         .
# AAH03551.1            122 S   SLEASTGYQ    0.091    PKB          .
# AAH03551.1            122 S   SLEASTGYQ    0.044    unsp         .
#
# AAH03551.1            123 T   LEASTGYQG    0.516    cdc2         YES
# AAH03551.1            123 T   LEASTGYQG    0.437    CaM-II       .
# AAH03551.1            123 T   LEASTGYQG    0.423    GSK3         .
# AAH03551.1            123 T   LEASTGYQG    0.376    CKII         .
# AAH03551.1            123 T   LEASTGYQG    0.366    CKI          .
# AAH03551.1            123 T   LEASTGYQG    0.353    DNAPK        .
# AAH03551.1            123 T   LEASTGYQG    0.239    p38MAPK      .
# AAH03551.1            123 T   LEASTGYQG    0.235    ATM          .
# AAH03551.1            123 T   LEASTGYQG    0.234    PKG          .
# AAH03551.1            123 T   LEASTGYQG    0.208    RSK          .
# AAH03551.1            123 T   LEASTGYQG    0.174    PKC          .
# AAH03551.1            123 T   LEASTGYQG    0.156    cdk5         .
# AAH03551.1            123 T   LEASTGYQG    0.141    unsp         .
# AAH03551.1            123 T   LEASTGYQG    0.119    PKA          .
# AAH03551.1            123 T   LEASTGYQG    0.093    PKB          .
#
# AAH03551.1            125 Y   ASTGYQGSS    0.473    EGFR         .
# AAH03551.1            125 Y   ASTGYQGSS    0.456    unsp         .
# AAH03551.1            125 Y   ASTGYQGSS    0.449    INSR         .
# AAH03551.1            125 Y   ASTGYQGSS    0.388    SRC          .
#
# AAH03551.1            128 S   GYQGSSFVL    0.453    CaM-II       .
# AAH03551.1            128 S   GYQGSSFVL    0.453    GSK3         .
# AAH03551.1            128 S   GYQGSSFVL    0.440    cdc2         .
# AAH03551.1            128 S   GYQGSSFVL    0.392    PKA          .
# AAH03551.1            128 S   GYQGSSFVL    0.381    CKI          .
# AAH03551.1            128 S   GYQGSSFVL    0.345    DNAPK        .
# AAH03551.1            128 S   GYQGSSFVL    0.342    CKII         .
# AAH03551.1            128 S   GYQGSSFVL    0.317    PKC          .
# AAH03551.1            128 S   GYQGSSFVL    0.311    p38MAPK      .
```

```
# AAH03551.1            128 S   GYQGSSFVL   0.294   RSK         .
# AAH03551.1            128 S   GYQGSSFVL   0.293   ATM         .
# AAH03551.1            128 S   GYQGSSFVL   0.292   PKG         .
# AAH03551.1            128 S   GYQGSSFVL   0.187   cdk5        .
# AAH03551.1            128 S   GYQGSSFVL   0.078   PKB         .
# AAH03551.1            128 S   GYQGSSFVL   0.004   unsp        .
#
# AAH03551.1            129 S   YQGSSFVLG   0.607   cdc2        YES
# AAH03551.1            129 S   YQGSSFVLG   0.438   GSK3        .
# AAH03551.1            129 S   YQGSSFVLG   0.427   CaM-II      .
# AAH03551.1            129 S   YQGSSFVLG   0.372   CKI         .
# AAH03551.1            129 S   YQGSSFVLG   0.345   DNAPK       .
# AAH03551.1            129 S   YQGSSFVLG   0.343   CKII        .
# AAH03551.1            129 S   YQGSSFVLG   0.307   ATM         .
# AAH03551.1            129 S   YQGSSFVLG   0.293   p38MAPK     .
# AAH03551.1            129 S   YQGSSFVLG   0.258   PKA         .
# AAH03551.1            129 S   YQGSSFVLG   0.255   PKG         .
# AAH03551.1            129 S   YQGSSFVLG   0.248   RSK         .
# AAH03551.1            129 S   YQGSSFVLG   0.222   PKC         .
# AAH03551.1            129 S   YQGSSFVLG   0.178   cdk5        .
# AAH03551.1            129 S   YQGSSFVLG   0.110   PKB         .
# AAH03551.1            129 S   YQGSSFVLG   0.021   unsp        .
#
# AAH03551.1            149 Y   ADAVYLDSE   0.636   unsp        YES
# AAH03551.1            149 Y   ADAVYLDSE   0.425   SRC         .
# AAH03551.1            149 Y   ADAVYLDSE   0.411   EGFR        .
# AAH03551.1            149 Y   ADAVYLDSE   0.389   INSR        .
#
# AAH03551.1            152 S   VYLDSEEER   0.813   unsp        YES
# AAH03551.1            152 S   VYLDSEEER   0.697   CKII        YES
# AAH03551.1            152 S   VYLDSEEER   0.460   CKI         .
# AAH03551.1            152 S   VYLDSEEER   0.430   GSK3        .
# AAH03551.1            152 S   VYLDSEEER   0.419   CaM-II      .
# AAH03551.1            152 S   VYLDSEEER   0.388   cdc2        .
# AAH03551.1            152 S   VYLDSEEER   0.380   RSK         .
# AAH03551.1            152 S   VYLDSEEER   0.342   p38MAPK     .
# AAH03551.1            152 S   VYLDSEEER   0.340   DNAPK       .
# AAH03551.1            152 S   VYLDSEEER   0.303   ATM         .
# AAH03551.1            152 S   VYLDSEEER   0.293   PKG         .
# AAH03551.1            152 S   VYLDSEEER   0.179   PKC         .
# AAH03551.1            152 S   VYLDSEEER   0.138   cdk5        .
# AAH03551.1            152 S   VYLDSEEER   0.123   PKA         .
# AAH03551.1            152 S   VYLDSEEER   0.079   PKB         .
#
# AAH03551.1            159 Y   ERQEYVLTQ   0.751   unsp        YES
# AAH03551.1            159 Y   ERQEYVLTQ   0.532   EGFR        YES
# AAH03551.1            159 Y   ERQEYVLTQ   0.455   INSR        .
# AAH03551.1            159 Y   ERQEYVLTQ   0.438   SRC         .
#
# AAH03551.1            162 T   EYVLTQQGF   0.531   DNAPK       YES
# AAH03551.1            162 T   EYVLTQQGF   0.475   ATM         .
# AAH03551.1            162 T   EYVLTQQGF   0.471   cdc2        .
# AAH03551.1            162 T   EYVLTQQGF   0.427   p38MAPK     .
# AAH03551.1            162 T   EYVLTQQGF   0.425   GSK3        .
# AAH03551.1            162 T   EYVLTQQGF   0.418   CaM-II      .
# AAH03551.1            162 T   EYVLTQQGF   0.374   CKI         .
# AAH03551.1            162 T   EYVLTQQGF   0.346   CKII        .
# AAH03551.1            162 T   EYVLTQQGF   0.297   PKG         .
# AAH03551.1            162 T   EYVLTQQGF   0.214   unsp        .
# AAH03551.1            162 T   EYVLTQQGF   0.201   RSK         .
# AAH03551.1            162 T   EYVLTQQGF   0.161   PKC         .
# AAH03551.1            162 T   EYVLTQQGF   0.155   cdk5        .
# AAH03551.1            162 T   EYVLTQQGF   0.104   PKA         .
# AAH03551.1            162 T   EYVLTQQGF   0.087   PKB         .
#
# AAH03551.1            168 Y   QGFIYQGSA   0.403   INSR        .
# AAH03551.1            168 Y   QGFIYQGSA   0.361   EGFR        .
# AAH03551.1            168 Y   QGFIYQGSA   0.358   SRC         .
# AAH03551.1            168 Y   QGFIYQGSA   0.165   unsp        .
```

```
#
# AAH03551.1             171 S   IYQGSAKFI   0.858   PKC        YES
# AAH03551.1             171 S   IYQGSAKFI   0.509   cdc2       YES
# AAH03551.1             171 S   IYQGSAKFI   0.470   CaM-II      .
# AAH03551.1             171 S   IYQGSAKFI   0.455   GSK3        .
# AAH03551.1             171 S   IYQGSAKFI   0.368   CKI         .
# AAH03551.1             171 S   IYQGSAKFI   0.354   DNAPK       .
# AAH03551.1             171 S   IYQGSAKFI   0.339   PKG         .
# AAH03551.1             171 S   IYQGSAKFI   0.324   RSK         .
# AAH03551.1             171 S   IYQGSAKFI   0.321   p38MAPK     .
# AAH03551.1             171 S   IYQGSAKFI   0.289   CKII        .
# AAH03551.1             171 S   IYQGSAKFI   0.286   ATM         .
# AAH03551.1             171 S   IYQGSAKFI   0.160   PKA         .
# AAH03551.1             171 S   IYQGSAKFI   0.156   cdk5        .
# AAH03551.1             171 S   IYQGSAKFI   0.130   unsp        .
# AAH03551.1             171 S   IYQGSAKFI   0.092   PKB         .
#
# AAH03551.1             212 S   GRDCSRRSS   0.978   unsp       YES
# AAH03551.1             212 S   GRDCSRRSS   0.746   PKC        YES
# AAH03551.1             212 S   GRDCSRRSS   0.509   RSK        YES
# AAH03551.1             212 S   GRDCSRRSS   0.485   GSK3        .
# AAH03551.1             212 S   GRDCSRRSS   0.449   CaM-II      .
# AAH03551.1             212 S   GRDCSRRSS   0.413   p38MAPK     .
# AAH03551.1             212 S   GRDCSRRSS   0.389   PKA         .
# AAH03551.1             212 S   GRDCSRRSS   0.387   PKG         .
# AAH03551.1             212 S   GRDCSRRSS   0.363   CKI         .
# AAH03551.1             212 S   GRDCSRRSS   0.354   DNAPK       .
# AAH03551.1             212 S   GRDCSRRSS   0.340   cdc2        .
# AAH03551.1             212 S   GRDCSRRSS   0.302   ATM         .
# AAH03551.1             212 S   GRDCSRRSS   0.241   cdk5        .
# AAH03551.1             212 S   GRDCSRRSS   0.210   PKB         .
# AAH03551.1             212 S   GRDCSRRSS   0.202   CKII        .
#
# AAH03551.1             215 S   CSRRSSPVY   0.923   unsp       YES
# AAH03551.1             215 S   CSRRSSPVY   0.683   PKA        YES
# AAH03551.1             215 S   CSRRSSPVY   0.485   PKG         .
# AAH03551.1             215 S   CSRRSSPVY   0.457   GSK3        .
# AAH03551.1             215 S   CSRRSSPVY   0.419   CaM-II      .
# AAH03551.1             215 S   CSRRSSPVY   0.418   cdc2        .
# AAH03551.1             215 S   CSRRSSPVY   0.398   PKC         .
# AAH03551.1             215 S   CSRRSSPVY   0.378   CKI         .
# AAH03551.1             215 S   CSRRSSPVY   0.362   DNAPK       .
# AAH03551.1             215 S   CSRRSSPVY   0.336   RSK         .
# AAH03551.1             215 S   CSRRSSPVY   0.330   p38MAPK     .
# AAH03551.1             215 S   CSRRSSPVY   0.312   ATM         .
# AAH03551.1             215 S   CSRRSSPVY   0.262   CKII        .
# AAH03551.1             215 S   CSRRSSPVY   0.247   cdk5        .
# AAH03551.1             215 S   CSRRSSPVY   0.108   PKB         .
#
# AAH03551.1             216 S   SRRSSPVYV   0.988   unsp       YES
# AAH03551.1             216 S   SRRSSPVYV   0.744   PKA        YES
# AAH03551.1             216 S   SRRSSPVYV   0.668   cdk5       YES
# AAH03551.1             216 S   SRRSSPVYV   0.625   RSK        YES
# AAH03551.1             216 S   SRRSSPVYV   0.507   GSK3       YES
# AAH03551.1             216 S   SRRSSPVYV   0.466   CaM-II      .
# AAH03551.1             216 S   SRRSSPVYV   0.453   p38MAPK     .
# AAH03551.1             216 S   SRRSSPVYV   0.440   cdc2        .
# AAH03551.1             216 S   SRRSSPVYV   0.388   PKG         .
# AAH03551.1             216 S   SRRSSPVYV   0.370   CKI         .
# AAH03551.1             216 S   SRRSSPVYV   0.361   DNAPK       .
# AAH03551.1             216 S   SRRSSPVYV   0.276   ATM         .
# AAH03551.1             216 S   SRRSSPVYV   0.227   PKB         .
# AAH03551.1             216 S   SRRSSPVYV   0.215   CKII        .
# AAH03551.1             216 S   SRRSSPVYV   0.089   PKC         .
#
# AAH03551.1             219 Y   SSPVYVGRV   0.695   unsp       YES
# AAH03551.1             219 Y   SSPVYVGRV   0.452   INSR        .
# AAH03551.1             219 Y   SSPVYVGRV   0.413   SRC         .
# AAH03551.1             219 Y   SSPVYVGRV   0.338   EGFR        .
```

```
#
# AAH03551.1            225 S   GRVVSGMVN   0.607   PKA        YES
# AAH03551.1            225 S   GRVVSGMVN   0.444   GSK3       .
# AAH03551.1            225 S   GRVVSGMVN   0.443   CaM-II     .
# AAH03551.1            225 S   GRVVSGMVN   0.412   RSK        .
# AAH03551.1            225 S   GRVVSGMVN   0.382   PKG        .
# AAH03551.1            225 S   GRVVSGMVN   0.362   CKI        .
# AAH03551.1            225 S   GRVVSGMVN   0.344   DNAPK      .
# AAH03551.1            225 S   GRVVSGMVN   0.335   cdc2       .
# AAH03551.1            225 S   GRVVSGMVN   0.303   p38MAPK    .
# AAH03551.1            225 S   GRVVSGMVN   0.286   PKB        .
# AAH03551.1            225 S   GRVVSGMVN   0.284   ATM        .
# AAH03551.1            225 S   GRVVSGMVN   0.279   CKII       .
# AAH03551.1            225 S   GRVVSGMVN   0.256   unsp       .
# AAH03551.1            225 S   GRVVSGMVN   0.200   PKC        .
# AAH03551.1            225 S   GRVVSGMVN   0.197   cdk5       .
#
# AAH03551.1            245 Y   WDNNYGDGV   0.521   INSR       YES
# AAH03551.1            245 Y   WDNNYGDGV   0.511   unsp       YES
# AAH03551.1            245 Y   WDNNYGDGV   0.430   SRC        .
# AAH03551.1            245 Y   WDNNYGDGV   0.336   EGFR       .
#
# AAH03551.1            250 S   GDGVSPMSW   0.522   p38MAPK    YES
# AAH03551.1            250 S   GDGVSPMSW   0.486   GSK3       .
# AAH03551.1            250 S   GDGVSPMSW   0.442   cdk5       .
# AAH03551.1            250 S   GDGVSPMSW   0.437   CaM-II     .
# AAH03551.1            250 S   GDGVSPMSW   0.430   cdc2       .
# AAH03551.1            250 S   GDGVSPMSW   0.406   CKI        .
# AAH03551.1            250 S   GDGVSPMSW   0.359   ATM        .
# AAH03551.1            250 S   GDGVSPMSW   0.340   DNAPK      .
# AAH03551.1            250 S   GDGVSPMSW   0.311   PKA        .
# AAH03551.1            250 S   GDGVSPMSW   0.287   RSK        .
# AAH03551.1            250 S   GDGVSPMSW   0.273   CKII       .
# AAH03551.1            250 S   GDGVSPMSW   0.244   unsp       .
# AAH03551.1            250 S   GDGVSPMSW   0.224   PKG        .
# AAH03551.1            250 S   GDGVSPMSW   0.105   PKB        .
# AAH03551.1            250 S   GDGVSPMSW   0.055   PKC        .
#
# AAH03551.1            253 S   VSPMSWIGS   0.992   unsp       YES
# AAH03551.1            253 S   VSPMSWIGS   0.581   CKI        YES
# AAH03551.1            253 S   VSPMSWIGS   0.484   GSK3       .
# AAH03551.1            253 S   VSPMSWIGS   0.442   cdc2       .
# AAH03551.1            253 S   VSPMSWIGS   0.439   CaM-II     .
# AAH03551.1            253 S   VSPMSWIGS   0.439   p38MAPK    .
# AAH03551.1            253 S   VSPMSWIGS   0.395   CKII       .
# AAH03551.1            253 S   VSPMSWIGS   0.354   DNAPK      .
# AAH03551.1            253 S   VSPMSWIGS   0.307   ATM        .
# AAH03551.1            253 S   VSPMSWIGS   0.307   PKC        .
# AAH03551.1            253 S   VSPMSWIGS   0.291   RSK        .
# AAH03551.1            253 S   VSPMSWIGS   0.274   cdk5       .
# AAH03551.1            253 S   VSPMSWIGS   0.231   PKG        .
# AAH03551.1            253 S   VSPMSWIGS   0.118   PKA        .
# AAH03551.1            253 S   VSPMSWIGS   0.086   PKB        .
#
# AAH03551.1            257 S   SWIGSVDIL   0.485   CaM-II     .
# AAH03551.1            257 S   SWIGSVDIL   0.451   GSK3       .
# AAH03551.1            257 S   SWIGSVDIL   0.434   DNAPK      .
# AAH03551.1            257 S   SWIGSVDIL   0.399   CKII       .
# AAH03551.1            257 S   SWIGSVDIL   0.366   RSK        .
# AAH03551.1            257 S   SWIGSVDIL   0.363   cdc2       .
# AAH03551.1            257 S   SWIGSVDIL   0.362   CKI        .
# AAH03551.1            257 S   SWIGSVDIL   0.361   ATM        .
# AAH03551.1            257 S   SWIGSVDIL   0.357   p38MAPK    .
# AAH03551.1            257 S   SWIGSVDIL   0.318   PKA        .
# AAH03551.1            257 S   SWIGSVDIL   0.291   PKC        .
# AAH03551.1            257 S   SWIGSVDIL   0.253   PKG        .
# AAH03551.1            257 S   SWIGSVDIL   0.240   cdk5       .
# AAH03551.1            257 S   SWIGSVDIL   0.083   PKB        .
# AAH03551.1            257 S   SWIGSVDIL   0.013   unsp       .
```

```
#
# AAH03551.1            274 Y   QRVKYGQCW   0.383   INSR       .
# AAH03551.1            274 Y   QRVKYGQCW   0.358   EGFR       .
# AAH03551.1            274 Y   QRVKYGQCW   0.338   SRC        .
# AAH03551.1            274 Y   QRVKYGQCW   0.053   unsp       .
#
# AAH03551.1            286 T   AVACTVLRC   0.621   PKC        YES
# AAH03551.1            286 T   AVACTVLRC   0.472   cdc2       .
# AAH03551.1            286 T   AVACTVLRC   0.442   CaM-II     .
# AAH03551.1            286 T   AVACTVLRC   0.438   GSK3       .
# AAH03551.1            286 T   AVACTVLRC   0.365   CKI        .
# AAH03551.1            286 T   AVACTVLRC   0.339   DNAPK      .
# AAH03551.1            286 T   AVACTVLRC   0.336   p38MAPK    .
# AAH03551.1            286 T   AVACTVLRC   0.286   CKII       .
# AAH03551.1            286 T   AVACTVLRC   0.249   PKG        .
# AAH03551.1            286 T   AVACTVLRC   0.237   ATM        .
# AAH03551.1            286 T   AVACTVLRC   0.221   RSK        .
# AAH03551.1            286 T   AVACTVLRC   0.184   cdk5       .
# AAH03551.1            286 T   AVACTVLRC   0.135   PKA        .
# AAH03551.1            286 T   AVACTVLRC   0.080   PKB        .
# AAH03551.1            286 T   AVACTVLRC   0.013   unsp       .
#
# AAH03551.1            295 T   LGIPTRVVT   0.517   unsp       YES
# AAH03551.1            295 T   LGIPTRVVT   0.470   cdc2       .
# AAH03551.1            295 T   LGIPTRVVT   0.450   GSK3       .
# AAH03551.1            295 T   LGIPTRVVT   0.425   CaM-II     .
# AAH03551.1            295 T   LGIPTRVVT   0.391   PKG        .
# AAH03551.1            295 T   LGIPTRVVT   0.352   CKI        .
# AAH03551.1            295 T   LGIPTRVVT   0.348   CKII       .
# AAH03551.1            295 T   LGIPTRVVT   0.342   DNAPK      .
# AAH03551.1            295 T   LGIPTRVVT   0.338   p38MAPK    .
# AAH03551.1            295 T   LGIPTRVVT   0.254   ATM        .
# AAH03551.1            295 T   LGIPTRVVT   0.166   cdk5       .
# AAH03551.1            295 T   LGIPTRVVT   0.162   RSK        .
# AAH03551.1            295 T   LGIPTRVVT   0.159   PKA        .
# AAH03551.1            295 T   LGIPTRVVT   0.118   PKC        .
# AAH03551.1            295 T   LGIPTRVVT   0.087   PKB        .
#
# AAH03551.1            299 T   TRVVTNYNS   0.622   PKG        YES
# AAH03551.1            299 T   TRVVTNYNS   0.477   CaM-II     .
# AAH03551.1            299 T   TRVVTNYNS   0.469   GSK3       .
# AAH03551.1            299 T   TRVVTNYNS   0.388   RSK        .
# AAH03551.1            299 T   TRVVTNYNS   0.356   CKI        .
# AAH03551.1            299 T   TRVVTNYNS   0.345   DNAPK      .
# AAH03551.1            299 T   TRVVTNYNS   0.338   cdc2       .
# AAH03551.1            299 T   TRVVTNYNS   0.289   p38MAPK    .
# AAH03551.1            299 T   TRVVTNYNS   0.269   PKA        .
# AAH03551.1            299 T   TRVVTNYNS   0.252   CKII       .
# AAH03551.1            299 T   TRVVTNYNS   0.249   ATM        .
# AAH03551.1            299 T   TRVVTNYNS   0.247   cdk5       .
# AAH03551.1            299 T   TRVVTNYNS   0.222   unsp       .
# AAH03551.1            299 T   TRVVTNYNS   0.175   PKB        .
# AAH03551.1            299 T   TRVVTNYNS   0.077   PKC        .
#
# AAH03551.1            301 Y   VVTNYNSAH   0.477   EGFR       .
# AAH03551.1            301 Y   VVTNYNSAH   0.433   INSR       .
# AAH03551.1            301 Y   VVTNYNSAH   0.375   SRC        .
# AAH03551.1            301 Y   VVTNYNSAH   0.055   unsp       .
#
# AAH03551.1            303 S   TNYNSAHDQ   0.636   unsp       YES
# AAH03551.1            303 S   TNYNSAHDQ   0.497   PKG        .
# AAH03551.1            303 S   TNYNSAHDQ   0.480   cdc2       .
# AAH03551.1            303 S   TNYNSAHDQ   0.463   GSK3       .
# AAH03551.1            303 S   TNYNSAHDQ   0.460   CaM-II     .
# AAH03551.1            303 S   TNYNSAHDQ   0.364   CKI        .
# AAH03551.1            303 S   TNYNSAHDQ   0.360   CKII       .
# AAH03551.1            303 S   TNYNSAHDQ   0.350   DNAPK      .
# AAH03551.1            303 S   TNYNSAHDQ   0.286   p38MAPK    .
# AAH03551.1            303 S   TNYNSAHDQ   0.279   ATM        .
```

```
# AAH03551.1            303 S   TNYNSAHDQ   0.274   RSK         .
# AAH03551.1            303 S   TNYNSAHDQ   0.174   cdk5        .
# AAH03551.1            303 S   TNYNSAHDQ   0.133   PKA         .
# AAH03551.1            303 S   TNYNSAHDQ   0.099   PKB         .
# AAH03551.1            303 S   TNYNSAHDQ   0.073   PKC         .
#
# AAH03551.1            309 S   HDQNSNLLI   0.491   cdc2        .
# AAH03551.1            309 S   HDQNSNLLI   0.473   CKII        .
# AAH03551.1            309 S   HDQNSNLLI   0.467   CaM-II      .
# AAH03551.1            309 S   HDQNSNLLI   0.447   GSK3        .
# AAH03551.1            309 S   HDQNSNLLI   0.369   CKI         .
# AAH03551.1            309 S   HDQNSNLLI   0.353   RSK         .
# AAH03551.1            309 S   HDQNSNLLI   0.350   DNAPK       .
# AAH03551.1            309 S   HDQNSNLLI   0.306   ATM         .
# AAH03551.1            309 S   HDQNSNLLI   0.269   p38MAPK     .
# AAH03551.1            309 S   HDQNSNLLI   0.253   PKG         .
# AAH03551.1            309 S   HDQNSNLLI   0.164   PKC         .
# AAH03551.1            309 S   HDQNSNLLI   0.151   PKA         .
# AAH03551.1            309 S   HDQNSNLLI   0.143   cdk5        .
# AAH03551.1            309 S   HDQNSNLLI   0.077   PKB         .
# AAH03551.1            309 S   HDQNSNLLI   0.011   unsp        .
#
# AAH03551.1            315 Y   LLIEYFRNE   0.465   INSR        .
# AAH03551.1            315 Y   LLIEYFRNE   0.394   SRC         .
# AAH03551.1            315 Y   LLIEYFRNE   0.331   EGFR        .
# AAH03551.1            315 Y   LLIEYFRNE   0.018   unsp        .
#
# AAH03551.1            328 S   QGDKSEMIW   0.504   CKII       YES
# AAH03551.1            328 S   QGDKSEMIW   0.435   CaM-II      .
# AAH03551.1            328 S   QGDKSEMIW   0.430   GSK3        .
# AAH03551.1            328 S   QGDKSEMIW   0.383   RSK         .
# AAH03551.1            328 S   QGDKSEMIW   0.381   cdc2        .
# AAH03551.1            328 S   QGDKSEMIW   0.361   CKI         .
# AAH03551.1            328 S   QGDKSEMIW   0.347   DNAPK       .
# AAH03551.1            328 S   QGDKSEMIW   0.326   ATM         .
# AAH03551.1            328 S   QGDKSEMIW   0.308   p38MAPK     .
# AAH03551.1            328 S   QGDKSEMIW   0.299   PKG         .
# AAH03551.1            328 S   QGDKSEMIW   0.206   PKA         .
# AAH03551.1            328 S   QGDKSEMIW   0.180   cdk5        .
# AAH03551.1            328 S   QGDKSEMIW   0.156   unsp        .
# AAH03551.1            328 S   QGDKSEMIW   0.089   PKB         .
# AAH03551.1            328 S   QGDKSEMIW   0.073   PKC         .
#
# AAH03551.1            340 S   CWVESWMTR   0.453   CKII        .
# AAH03551.1            340 S   CWVESWMTR   0.448   CaM-II      .
# AAH03551.1            340 S   CWVESWMTR   0.438   GSK3        .
# AAH03551.1            340 S   CWVESWMTR   0.421   cdc2        .
# AAH03551.1            340 S   CWVESWMTR   0.398   CKI         .
# AAH03551.1            340 S   CWVESWMTR   0.390   p38MAPK     .
# AAH03551.1            340 S   CWVESWMTR   0.357   DNAPK       .
# AAH03551.1            340 S   CWVESWMTR   0.308   PKG         .
# AAH03551.1            340 S   CWVESWMTR   0.273   ATM         .
# AAH03551.1            340 S   CWVESWMTR   0.271   cdk5        .
# AAH03551.1            340 S   CWVESWMTR   0.262   RSK         .
# AAH03551.1            340 S   CWVESWMTR   0.255   PKA         .
# AAH03551.1            340 S   CWVESWMTR   0.215   PKC         .
# AAH03551.1            340 S   CWVESWMTR   0.086   PKB         .
# AAH03551.1            340 S   CWVESWMTR   0.004   unsp        .
#
# AAH03551.1            343 T   ESWMTRPDL   0.448   GSK3        .
# AAH03551.1            343 T   ESWMTRPDL   0.446   CaM-II      .
# AAH03551.1            343 T   ESWMTRPDL   0.407   DNAPK       .
# AAH03551.1            343 T   ESWMTRPDL   0.369   CKI         .
# AAH03551.1            343 T   ESWMTRPDL   0.368   p38MAPK     .
# AAH03551.1            343 T   ESWMTRPDL   0.348   CKII        .
# AAH03551.1            343 T   ESWMTRPDL   0.324   cdc2        .
# AAH03551.1            343 T   ESWMTRPDL   0.293   unsp        .
# AAH03551.1            343 T   ESWMTRPDL   0.256   PKG         .
# AAH03551.1            343 T   ESWMTRPDL   0.243   ATM         .
```

```
# AAH03551.1          343 T   ESWMTRPDL   0.228   RSK       .
# AAH03551.1          343 T   ESWMTRPDL   0.191   cdk5      .
# AAH03551.1          343 T   ESWMTRPDL   0.131   PKC       .
# AAH03551.1          343 T   ESWMTRPDL   0.128   PKA       .
# AAH03551.1          343 T   ESWMTRPDL   0.087   PKB       .
#
# AAH03551.1          351 Y   LQPGYEGWQ   0.412   SRC       .
# AAH03551.1          351 Y   LQPGYEGWQ   0.388   INSR      .
# AAH03551.1          351 Y   LQPGYEGWQ   0.377   EGFR      .
# AAH03551.1          351 Y   LQPGYEGWQ   0.192   unsp      .
#
# AAH03551.1          360 T   ALDPTPQEK   0.507   GSK3      YES
# AAH03551.1          360 T   ALDPTPQEK   0.461   p38MAPK   .
# AAH03551.1          360 T   ALDPTPQEK   0.459   CKII      .
# AAH03551.1          360 T   ALDPTPQEK   0.440   cdk5      .
# AAH03551.1          360 T   ALDPTPQEK   0.423   cdc2      .
# AAH03551.1          360 T   ALDPTPQEK   0.410   CaM-II    .
# AAH03551.1          360 T   ALDPTPQEK   0.379   PKG       .
# AAH03551.1          360 T   ALDPTPQEK   0.362   CKI       .
# AAH03551.1          360 T   ALDPTPQEK   0.351   DNAPK     .
# AAH03551.1          360 T   ALDPTPQEK   0.316   unsp      .
# AAH03551.1          360 T   ALDPTPQEK   0.295   ATM       .
# AAH03551.1          360 T   ALDPTPQEK   0.208   RSK       .
# AAH03551.1          360 T   ALDPTPQEK   0.083   PKA       .
# AAH03551.1          360 T   ALDPTPQEK   0.083   PKB       .
# AAH03551.1          360 T   ALDPTPQEK   0.048   PKC       .
#
# AAH03551.1          365 S   PQEKSEGTY   0.595   unsp      YES
# AAH03551.1          365 S   PQEKSEGTY   0.524   CKI       YES
# AAH03551.1          365 S   PQEKSEGTY   0.437   CaM-II    .
# AAH03551.1          365 S   PQEKSEGTY   0.436   GSK3      .
# AAH03551.1          365 S   PQEKSEGTY   0.418   CKII      .
# AAH03551.1          365 S   PQEKSEGTY   0.384   cdc2      .
# AAH03551.1          365 S   PQEKSEGTY   0.344   DNAPK     .
# AAH03551.1          365 S   PQEKSEGTY   0.311   PKG       .
# AAH03551.1          365 S   PQEKSEGTY   0.305   PKA       .
# AAH03551.1          365 S   PQEKSEGTY   0.281   RSK       .
# AAH03551.1          365 S   PQEKSEGTY   0.270   p38MAPK   .
# AAH03551.1          365 S   PQEKSEGTY   0.254   ATM       .
# AAH03551.1          365 S   PQEKSEGTY   0.189   cdk5      .
# AAH03551.1          365 S   PQEKSEGTY   0.097   PKB       .
# AAH03551.1          365 S   PQEKSEGTY   0.080   PKC       .
#
# AAH03551.1          368 T   KSEGTYCCG   0.439   CaM-II    .
# AAH03551.1          368 T   KSEGTYCCG   0.438   GSK3      .
# AAH03551.1          368 T   KSEGTYCCG   0.417   cdc2      .
# AAH03551.1          368 T   KSEGTYCCG   0.409   CKI       .
# AAH03551.1          368 T   KSEGTYCCG   0.407   unsp      .
# AAH03551.1          368 T   KSEGTYCCG   0.382   DNAPK     .
# AAH03551.1          368 T   KSEGTYCCG   0.364   p38MAPK   .
# AAH03551.1          368 T   KSEGTYCCG   0.356   CKII      .
# AAH03551.1          368 T   KSEGTYCCG   0.300   PKC       .
# AAH03551.1          368 T   KSEGTYCCG   0.268   PKG       .
# AAH03551.1          368 T   KSEGTYCCG   0.265   ATM       .
# AAH03551.1          368 T   KSEGTYCCG   0.258   RSK       .
# AAH03551.1          368 T   KSEGTYCCG   0.154   cdk5      .
# AAH03551.1          368 T   KSEGTYCCG   0.136   PKA       .
# AAH03551.1          368 T   KSEGTYCCG   0.099   PKB       .
#
# AAH03551.1          369 Y   SEGTYCCGP   0.971   unsp      YES
# AAH03551.1          369 Y   SEGTYCCGP   0.504   SRC       YES
# AAH03551.1          369 Y   SEGTYCCGP   0.416   INSR      .
# AAH03551.1          369 Y   SEGTYCCGP   0.347   EGFR      .
#
# AAH03551.1          385 S   EGDLSTKYD   0.986   unsp      YES
# AAH03551.1          385 S   EGDLSTKYD   0.444   GSK3      .
# AAH03551.1          385 S   EGDLSTKYD   0.439   CKII      .
# AAH03551.1          385 S   EGDLSTKYD   0.419   CaM-II    .
# AAH03551.1          385 S   EGDLSTKYD   0.385   PKC       .
```

```
# AAH03551.1             385 S   EGDLSTKYD   0.380   cdc2         .
# AAH03551.1             385 S   EGDLSTKYD   0.355   DNAPK        .
# AAH03551.1             385 S   EGDLSTKYD   0.354   CKI          .
# AAH03551.1             385 S   EGDLSTKYD   0.353   RSK          .
# AAH03551.1             385 S   EGDLSTKYD   0.335   ATM          .
# AAH03551.1             385 S   EGDLSTKYD   0.315   PKG          .
# AAH03551.1             385 S   EGDLSTKYD   0.304   PKA          .
# AAH03551.1             385 S   EGDLSTKYD   0.289   p38MAPK      .
# AAH03551.1             385 S   EGDLSTKYD   0.141   cdk5         .
# AAH03551.1             385 S   EGDLSTKYD   0.090   PKB          .
#
# AAH03551.1             386 T   GDLSTKYDA   0.738   PKC         YES
# AAH03551.1             386 T   GDLSTKYDA   0.482   CKI          .
# AAH03551.1             386 T   GDLSTKYDA   0.457   unsp         .
# AAH03551.1             386 T   GDLSTKYDA   0.432   CaM-II       .
# AAH03551.1             386 T   GDLSTKYDA   0.426   GSK3         .
# AAH03551.1             386 T   GDLSTKYDA   0.423   CKII         .
# AAH03551.1             386 T   GDLSTKYDA   0.415   cdc2         .
# AAH03551.1             386 T   GDLSTKYDA   0.371   p38MAPK      .
# AAH03551.1             386 T   GDLSTKYDA   0.344   DNAPK        .
# AAH03551.1             386 T   GDLSTKYDA   0.263   PKG          .
# AAH03551.1             386 T   GDLSTKYDA   0.257   ATM          .
# AAH03551.1             386 T   GDLSTKYDA   0.236   RSK          .
# AAH03551.1             386 T   GDLSTKYDA   0.179   cdk5         .
# AAH03551.1             386 T   GDLSTKYDA   0.097   PKB          .
# AAH03551.1             386 T   GDLSTKYDA   0.096   PKA          .
#
# AAH03551.1             388 Y   LSTKYDAPF   0.410   SRC          .
# AAH03551.1             388 Y   LSTKYDAPF   0.377   INSR         .
# AAH03551.1             388 Y   LSTKYDAPF   0.348   EGFR         .
# AAH03551.1             388 Y   LSTKYDAPF   0.173   unsp         .
#
# AAH03551.1             411 S   QDDGSVHKS   0.730   PKC         YES
# AAH03551.1             411 S   QDDGSVHKS   0.618   unsp        YES
# AAH03551.1             411 S   QDDGSVHKS   0.496   cdc2         .
# AAH03551.1             411 S   QDDGSVHKS   0.463   GSK3         .
# AAH03551.1             411 S   QDDGSVHKS   0.424   CaM-II       .
# AAH03551.1             411 S   QDDGSVHKS   0.376   p38MAPK      .
# AAH03551.1             411 S   QDDGSVHKS   0.374   CKI          .
# AAH03551.1             411 S   QDDGSVHKS   0.370   DNAPK        .
# AAH03551.1             411 S   QDDGSVHKS   0.345   ATM          .
# AAH03551.1             411 S   QDDGSVHKS   0.327   RSK          .
# AAH03551.1             411 S   QDDGSVHKS   0.313   PKG          .
# AAH03551.1             411 S   QDDGSVHKS   0.307   CKII         .
# AAH03551.1             411 S   QDDGSVHKS   0.201   cdk5         .
# AAH03551.1             411 S   QDDGSVHKS   0.133   PKA          .
# AAH03551.1             411 S   QDDGSVHKS   0.084   PKB          .
#
# AAH03551.1             415 S   SVHKSINRS   0.670   unsp        YES
# AAH03551.1             415 S   SVHKSINRS   0.473   GSK3         .
# AAH03551.1             415 S   SVHKSINRS   0.447   cdc2         .
# AAH03551.1             415 S   SVHKSINRS   0.443   CaM-II       .
# AAH03551.1             415 S   SVHKSINRS   0.378   CKI          .
# AAH03551.1             415 S   SVHKSINRS   0.355   CKII         .
# AAH03551.1             415 S   SVHKSINRS   0.345   DNAPK        .
# AAH03551.1             415 S   SVHKSINRS   0.326   PKC          .
# AAH03551.1             415 S   SVHKSINRS   0.319   p38MAPK      .
# AAH03551.1             415 S   SVHKSINRS   0.310   RSK          .
# AAH03551.1             415 S   SVHKSINRS   0.302   ATM          .
# AAH03551.1             415 S   SVHKSINRS   0.291   cdk5         .
# AAH03551.1             415 S   SVHKSINRS   0.269   PKG          .
# AAH03551.1             415 S   SVHKSINRS   0.187   PKA          .
# AAH03551.1             415 S   SVHKSINRS   0.092   PKB          .
#
# AAH03551.1             419 S   SINRSLIVG   0.614   PKA         YES
# AAH03551.1             419 S   SINRSLIVG   0.465   GSK3         .
# AAH03551.1             419 S   SINRSLIVG   0.455   cdc2         .
# AAH03551.1             419 S   SINRSLIVG   0.438   PKG          .
# AAH03551.1             419 S   SINRSLIVG   0.397   DNAPK        .
```

```
# AAH03551.1            419 S   SINRSLIVG   0.393   CaM-II      .
# AAH03551.1            419 S   SINRSLIVG   0.376   CKI         .
# AAH03551.1            419 S   SINRSLIVG   0.310   PKC         .
# AAH03551.1            419 S   SINRSLIVG   0.268   ATM         .
# AAH03551.1            419 S   SINRSLIVG   0.263   CKII        .
# AAH03551.1            419 S   SINRSLIVG   0.262   p38MAPK     .
# AAH03551.1            419 S   SINRSLIVG   0.249   unsp        .
# AAH03551.1            419 S   SINRSLIVG   0.245   RSK         .
# AAH03551.1            419 S   SINRSLIVG   0.216   cdk5        .
# AAH03551.1            419 S   SINRSLIVG   0.078   PKB         .
#
# AAH03551.1            427 S   GLKISTKSV   0.670   PKC        YES
# AAH03551.1            427 S   GLKISTKSV   0.501   PKG        YES
# AAH03551.1            427 S   GLKISTKSV   0.461   cdc2        .
# AAH03551.1            427 S   GLKISTKSV   0.451   GSK3        .
# AAH03551.1            427 S   GLKISTKSV   0.438   CaM-II      .
# AAH03551.1            427 S   GLKISTKSV   0.368   PKA         .
# AAH03551.1            427 S   GLKISTKSV   0.358   CKI         .
# AAH03551.1            427 S   GLKISTKSV   0.339   DNAPK       .
# AAH03551.1            427 S   GLKISTKSV   0.311   RSK         .
# AAH03551.1            427 S   GLKISTKSV   0.297   p38MAPK     .
# AAH03551.1            427 S   GLKISTKSV   0.288   ATM         .
# AAH03551.1            427 S   GLKISTKSV   0.230   CKII        .
# AAH03551.1            427 S   GLKISTKSV   0.174   cdk5        .
# AAH03551.1            427 S   GLKISTKSV   0.108   unsp        .
# AAH03551.1            427 S   GLKISTKSV   0.091   PKB         .
#
# AAH03551.1            428 T   LKISTKSVG   0.496   cdc2        .
# AAH03551.1            428 T   LKISTKSVG   0.434   GSK3        .
# AAH03551.1            428 T   LKISTKSVG   0.433   CaM-II      .
# AAH03551.1            428 T   LKISTKSVG   0.376   DNAPK       .
# AAH03551.1            428 T   LKISTKSVG   0.362   CKI         .
# AAH03551.1            428 T   LKISTKSVG   0.357   CKII        .
# AAH03551.1            428 T   LKISTKSVG   0.342   PKG         .
# AAH03551.1            428 T   LKISTKSVG   0.309   p38MAPK     .
# AAH03551.1            428 T   LKISTKSVG   0.245   ATM         .
# AAH03551.1            428 T   LKISTKSVG   0.216   PKC         .
# AAH03551.1            428 T   LKISTKSVG   0.189   RSK         .
# AAH03551.1            428 T   LKISTKSVG   0.188   cdk5        .
# AAH03551.1            428 T   LKISTKSVG   0.144   PKA         .
# AAH03551.1            428 T   LKISTKSVG   0.136   unsp        .
# AAH03551.1            428 T   LKISTKSVG   0.086   PKB         .
#
# AAH03551.1            430 S   ISTKSVGRD   0.993   unsp       YES
# AAH03551.1            430 S   ISTKSVGRD   0.797   PKC        YES
# AAH03551.1            430 S   ISTKSVGRD   0.447   CaM-II      .
# AAH03551.1            430 S   ISTKSVGRD   0.442   GSK3        .
# AAH03551.1            430 S   ISTKSVGRD   0.371   CKI         .
# AAH03551.1            430 S   ISTKSVGRD   0.369   cdc2        .
# AAH03551.1            430 S   ISTKSVGRD   0.359   RSK         .
# AAH03551.1            430 S   ISTKSVGRD   0.348   DNAPK       .
# AAH03551.1            430 S   ISTKSVGRD   0.336   CKII        .
# AAH03551.1            430 S   ISTKSVGRD   0.334   p38MAPK     .
# AAH03551.1            430 S   ISTKSVGRD   0.288   ATM         .
# AAH03551.1            430 S   ISTKSVGRD   0.233   PKA         .
# AAH03551.1            430 S   ISTKSVGRD   0.208   PKG         .
# AAH03551.1            430 S   ISTKSVGRD   0.197   cdk5        .
# AAH03551.1            430 S   ISTKSVGRD   0.092   PKB         .
#
# AAH03551.1            440 T   REDITHTYK   0.445   GSK3        .
# AAH03551.1            440 T   REDITHTYK   0.445   CaM-II      .
# AAH03551.1            440 T   REDITHTYK   0.409   CKII        .
# AAH03551.1            440 T   REDITHTYK   0.404   cdc2        .
# AAH03551.1            440 T   REDITHTYK   0.370   CKI         .
# AAH03551.1            440 T   REDITHTYK   0.347   DNAPK       .
# AAH03551.1            440 T   REDITHTYK   0.329   p38MAPK     .
# AAH03551.1            440 T   REDITHTYK   0.313   PKG         .
# AAH03551.1            440 T   REDITHTYK   0.256   ATM         .
# AAH03551.1            440 T   REDITHTYK   0.248   RSK         .
```

```
# AAH03551.1             440 T   REDITHTYK   0.184   cdk5         .
# AAH03551.1             440 T   REDITHTYK   0.168   PKC          .
# AAH03551.1             440 T   REDITHTYK   0.110   PKA          .
# AAH03551.1             440 T   REDITHTYK   0.091   PKB          .
# AAH03551.1             440 T   REDITHTYK   0.027   unsp         .
#
# AAH03551.1             442 T   DITHTYKYP   0.867   PKC         YES
# AAH03551.1             442 T   DITHTYKYP   0.486   cdc2         .
# AAH03551.1             442 T   DITHTYKYP   0.440   GSK3         .
# AAH03551.1             442 T   DITHTYKYP   0.439   CaM-II       .
# AAH03551.1             442 T   DITHTYKYP   0.385   CKII         .
# AAH03551.1             442 T   DITHTYKYP   0.368   DNAPK        .
# AAH03551.1             442 T   DITHTYKYP   0.361   CKI          .
# AAH03551.1             442 T   DITHTYKYP   0.325   p38MAPK      .
# AAH03551.1             442 T   DITHTYKYP   0.291   ATM          .
# AAH03551.1             442 T   DITHTYKYP   0.244   RSK          .
# AAH03551.1             442 T   DITHTYKYP   0.239   PKG          .
# AAH03551.1             442 T   DITHTYKYP   0.212   cdk5         .
# AAH03551.1             442 T   DITHTYKYP   0.141   unsp         .
# AAH03551.1             442 T   DITHTYKYP   0.139   PKA          .
# AAH03551.1             442 T   DITHTYKYP   0.098   PKB          .
#
# AAH03551.1             443 Y   ITHTYKYPE   0.429   SRC          .
# AAH03551.1             443 Y   ITHTYKYPE   0.423   INSR         .
# AAH03551.1             443 Y   ITHTYKYPE   0.359   EGFR         .
# AAH03551.1             443 Y   ITHTYKYPE   0.080   unsp         .
#
# AAH03551.1             445 Y   HTYKYPEGS   0.437   INSR         .
# AAH03551.1             445 Y   HTYKYPEGS   0.345   EGFR         .
# AAH03551.1             445 Y   HTYKYPEGS   0.317   SRC          .
# AAH03551.1             445 Y   HTYKYPEGS   0.055   unsp         .
#
# AAH03551.1             449 S   YPEGSSEER   0.994   unsp        YES
# AAH03551.1             449 S   YPEGSSEER   0.640   CKII        YES
# AAH03551.1             449 S   YPEGSSEER   0.476   cdc2         .
# AAH03551.1             449 S   YPEGSSEER   0.462   GSK3         .
# AAH03551.1             449 S   YPEGSSEER   0.402   CaM-II       .
# AAH03551.1             449 S   YPEGSSEER   0.386   DNAPK        .
# AAH03551.1             449 S   YPEGSSEER   0.384   CKI          .
# AAH03551.1             449 S   YPEGSSEER   0.342   PKG          .
# AAH03551.1             449 S   YPEGSSEER   0.277   ATM          .
# AAH03551.1             449 S   YPEGSSEER   0.272   p38MAPK      .
# AAH03551.1             449 S   YPEGSSEER   0.237   RSK          .
# AAH03551.1             449 S   YPEGSSEER   0.201   PKA          .
# AAH03551.1             449 S   YPEGSSEER   0.161   cdk5         .
# AAH03551.1             449 S   YPEGSSEER   0.088   PKC          .
# AAH03551.1             449 S   YPEGSSEER   0.078   PKB          .
#
# AAH03551.1             450 S   PEGSSEERE   0.987   unsp        YES
# AAH03551.1             450 S   PEGSSEERE   0.562   CKII        YES
# AAH03551.1             450 S   PEGSSEERE   0.474   cdc2         .
# AAH03551.1             450 S   PEGSSEERE   0.431   CaM-II       .
# AAH03551.1             450 S   PEGSSEERE   0.428   GSK3         .
# AAH03551.1             450 S   PEGSSEERE   0.363   CKI          .
# AAH03551.1             450 S   PEGSSEERE   0.351   DNAPK        .
# AAH03551.1             450 S   PEGSSEERE   0.299   RSK          .
# AAH03551.1             450 S   PEGSSEERE   0.297   ATM          .
# AAH03551.1             450 S   PEGSSEERE   0.268   p38MAPK      .
# AAH03551.1             450 S   PEGSSEERE   0.205   PKG          .
# AAH03551.1             450 S   PEGSSEERE   0.177   cdk5         .
# AAH03551.1             450 S   PEGSSEERE   0.115   PKA          .
# AAH03551.1             450 S   PEGSSEERE   0.083   PKB          .
# AAH03551.1             450 S   PEGSSEERE   0.063   PKC          .
#
# AAH03551.1             457 T   REAFTRANH   0.463   GSK3         .
# AAH03551.1             457 T   REAFTRANH   0.444   cdc2         .
# AAH03551.1             457 T   REAFTRANH   0.443   CaM-II       .
# AAH03551.1             457 T   REAFTRANH   0.412   CKI          .
# AAH03551.1             457 T   REAFTRANH   0.384   CKII         .
```

```
# AAH03551.1          457 T   REAFTRANH   0.353   PKC         .
# AAH03551.1          457 T   REAFTRANH   0.344   DNAPK       .
# AAH03551.1          457 T   REAFTRANH   0.315   p38MAPK     .
# AAH03551.1          457 T   REAFTRANH   0.280   PKG         .
# AAH03551.1          457 T   REAFTRANH   0.265   ATM         .
# AAH03551.1          457 T   REAFTRANH   0.222   PKA         .
# AAH03551.1          457 T   REAFTRANH   0.189   RSK         .
# AAH03551.1          457 T   REAFTRANH   0.168   cdk5        .
# AAH03551.1          457 T   REAFTRANH   0.089   PKB         .
# AAH03551.1          457 T   REAFTRANH   0.036   unsp        .
#
# AAH03551.1          471 T   EKEETGMAM   0.449   CKII        .
# AAH03551.1          471 T   EKEETGMAM   0.440   GSK3        .
# AAH03551.1          471 T   EKEETGMAM   0.429   CaM-II      .
# AAH03551.1          471 T   EKEETGMAM   0.371   CKI         .
# AAH03551.1          471 T   EKEETGMAM   0.345   DNAPK       .
# AAH03551.1          471 T   EKEETGMAM   0.328   cdc2        .
# AAH03551.1          471 T   EKEETGMAM   0.296   p38MAPK     .
# AAH03551.1          471 T   EKEETGMAM   0.232   PKG         .
# AAH03551.1          471 T   EKEETGMAM   0.232   ATM         .
# AAH03551.1          471 T   EKEETGMAM   0.218   RSK         .
# AAH03551.1          471 T   EKEETGMAM   0.140   cdk5        .
# AAH03551.1          471 T   EKEETGMAM   0.113   PKA         .
# AAH03551.1          471 T   EKEETGMAM   0.111   PKC         .
# AAH03551.1          471 T   EKEETGMAM   0.098   unsp        .
# AAH03551.1          471 T   EKEETGMAM   0.087   PKB         .
#
# AAH03551.1          482 S   RVGQSMNMG   0.483   cdc2        .
# AAH03551.1          482 S   RVGQSMNMG   0.472   unsp        .
# AAH03551.1          482 S   RVGQSMNMG   0.444   CaM-II      .
# AAH03551.1          482 S   RVGQSMNMG   0.430   GSK3        .
# AAH03551.1          482 S   RVGQSMNMG   0.383   DNAPK       .
# AAH03551.1          482 S   RVGQSMNMG   0.364   CKI         .
# AAH03551.1          482 S   RVGQSMNMG   0.327   CKII        .
# AAH03551.1          482 S   RVGQSMNMG   0.320   RSK         .
# AAH03551.1          482 S   RVGQSMNMG   0.285   ATM         .
# AAH03551.1          482 S   RVGQSMNMG   0.277   p38MAPK     .
# AAH03551.1          482 S   RVGQSMNMG   0.275   PKA         .
# AAH03551.1          482 S   RVGQSMNMG   0.258   PKG         .
# AAH03551.1          482 S   RVGQSMNMG   0.248   PKC         .
# AAH03551.1          482 S   RVGQSMNMG   0.181   cdk5        .
# AAH03551.1          482 S   RVGQSMNMG   0.114   PKB         .
#
# AAH03551.1          487 S   MNMGSDFDV   0.457   CKII        .
# AAH03551.1          487 S   MNMGSDFDV   0.438   GSK3        .
# AAH03551.1          487 S   MNMGSDFDV   0.429   CaM-II      .
# AAH03551.1          487 S   MNMGSDFDV   0.400   cdc2        .
# AAH03551.1          487 S   MNMGSDFDV   0.368   CKI         .
# AAH03551.1          487 S   MNMGSDFDV   0.341   DNAPK       .
# AAH03551.1          487 S   MNMGSDFDV   0.323   p38MAPK     .
# AAH03551.1          487 S   MNMGSDFDV   0.315   PKG         .
# AAH03551.1          487 S   MNMGSDFDV   0.298   ATM         .
# AAH03551.1          487 S   MNMGSDFDV   0.273   unsp        .
# AAH03551.1          487 S   MNMGSDFDV   0.251   RSK         .
# AAH03551.1          487 S   MNMGSDFDV   0.235   PKA         .
# AAH03551.1          487 S   MNMGSDFDV   0.174   cdk5        .
# AAH03551.1          487 S   MNMGSDFDV   0.084   PKC         .
# AAH03551.1          487 S   MNMGSDFDV   0.083   PKB         .
#
# AAH03551.1          496 T   FAHITNNTA   0.502   CKII        YES
# AAH03551.1          496 T   FAHITNNTA   0.450   CaM-II      .
# AAH03551.1          496 T   FAHITNNTA   0.429   GSK3        .
# AAH03551.1          496 T   FAHITNNTA   0.400   CKI         .
# AAH03551.1          496 T   FAHITNNTA   0.362   cdc2        .
# AAH03551.1          496 T   FAHITNNTA   0.353   p38MAPK     .
# AAH03551.1          496 T   FAHITNNTA   0.337   DNAPK       .
# AAH03551.1          496 T   FAHITNNTA   0.330   PKG         .
# AAH03551.1          496 T   FAHITNNTA   0.262   ATM         .
# AAH03551.1          496 T   FAHITNNTA   0.236   RSK         .
```

```
# AAH03551.1          496 T   FAHITNNTA   0.180   cdk5        .
# AAH03551.1          496 T   FAHITNNTA   0.123   PKA         .
# AAH03551.1          496 T   FAHITNNTA   0.082   PKC         .
# AAH03551.1          496 T   FAHITNNTA   0.078   PKB         .
# AAH03551.1          496 T   FAHITNNTA   0.017   unsp        .
#
# AAH03551.1          499 T   ITNNTAEEY   0.492   CKII        .
# AAH03551.1          499 T   ITNNTAEEY   0.448   PKG         .
# AAH03551.1          499 T   ITNNTAEEY   0.421   GSK3        .
# AAH03551.1          499 T   ITNNTAEEY   0.418   CaM-II      .
# AAH03551.1          499 T   ITNNTAEEY   0.392   CKI         .
# AAH03551.1          499 T   ITNNTAEEY   0.379   DNAPK       .
# AAH03551.1          499 T   ITNNTAEEY   0.376   cdc2        .
# AAH03551.1          499 T   ITNNTAEEY   0.330   unsp        .
# AAH03551.1          499 T   ITNNTAEEY   0.314   p38MAPK     .
# AAH03551.1          499 T   ITNNTAEEY   0.249   ATM         .
# AAH03551.1          499 T   ITNNTAEEY   0.206   RSK         .
# AAH03551.1          499 T   ITNNTAEEY   0.188   PKA         .
# AAH03551.1          499 T   ITNNTAEEY   0.153   cdk5        .
# AAH03551.1          499 T   ITNNTAEEY   0.094   PKC         .
# AAH03551.1          499 T   ITNNTAEEY   0.091   PKB         .
#
# AAH03551.1          503 Y   TAEEYVCRL   0.877   unsp        YES
# AAH03551.1          503 Y   TAEEYVCRL   0.442   INSR        .
# AAH03551.1          503 Y   TAEEYVCRL   0.376   SRC         .
# AAH03551.1          503 Y   TAEEYVCRL   0.373   EGFR        .
#
# AAH03551.1          513 T   LCARTVSYN   0.491   PKC         .
# AAH03551.1          513 T   LCARTVSYN   0.477   cdc2        .
# AAH03551.1          513 T   LCARTVSYN   0.469   CaM-II      .
# AAH03551.1          513 T   LCARTVSYN   0.444   GSK3        .
# AAH03551.1          513 T   LCARTVSYN   0.367   CKI         .
# AAH03551.1          513 T   LCARTVSYN   0.344   DNAPK       .
# AAH03551.1          513 T   LCARTVSYN   0.320   PKG         .
# AAH03551.1          513 T   LCARTVSYN   0.288   CKII        .
# AAH03551.1          513 T   LCARTVSYN   0.276   p38MAPK     .
# AAH03551.1          513 T   LCARTVSYN   0.230   RSK         .
# AAH03551.1          513 T   LCARTVSYN   0.230   ATM         .
# AAH03551.1          513 T   LCARTVSYN   0.180   PKA         .
# AAH03551.1          513 T   LCARTVSYN   0.179   cdk5        .
# AAH03551.1          513 T   LCARTVSYN   0.080   PKB         .
# AAH03551.1          513 T   LCARTVSYN   0.009   unsp        .
#
# AAH03551.1          515 S   ARTVSYNGI   0.655   PKA         YES
# AAH03551.1          515 S   ARTVSYNGI   0.487   CaM-II      .
# AAH03551.1          515 S   ARTVSYNGI   0.448   GSK3        .
# AAH03551.1          515 S   ARTVSYNGI   0.443   RSK         .
# AAH03551.1          515 S   ARTVSYNGI   0.427   ATM         .
# AAH03551.1          515 S   ARTVSYNGI   0.419   DNAPK       .
# AAH03551.1          515 S   ARTVSYNGI   0.391   cdc2        .
# AAH03551.1          515 S   ARTVSYNGI   0.389   PKG         .
# AAH03551.1          515 S   ARTVSYNGI   0.379   CKI         .
# AAH03551.1          515 S   ARTVSYNGI   0.281   p38MAPK     .
# AAH03551.1          515 S   ARTVSYNGI   0.257   PKB         .
# AAH03551.1          515 S   ARTVSYNGI   0.230   CKII        .
# AAH03551.1          515 S   ARTVSYNGI   0.204   cdk5        .
# AAH03551.1          515 S   ARTVSYNGI   0.176   PKC         .
# AAH03551.1          515 S   ARTVSYNGI   0.106   unsp        .
#
# AAH03551.1          516 Y   RTVSYNGIL   0.482   INSR        .
# AAH03551.1          516 Y   RTVSYNGIL   0.344   EGFR        .
# AAH03551.1          516 Y   RTVSYNGIL   0.317   SRC         .
# AAH03551.1          516 Y   RTVSYNGIL   0.072   unsp        .
#
# AAH03551.1          526 T   PECGTKYLL   0.432   CaM-II      .
# AAH03551.1          526 T   PECGTKYLL   0.431   GSK3        .
# AAH03551.1          526 T   PECGTKYLL   0.422   cdc2        .
# AAH03551.1          526 T   PECGTKYLL   0.408   unsp        .
# AAH03551.1          526 T   PECGTKYLL   0.404   CKII        .
```

```
# AAH03551.1         526 T   PECGTKYLL   0.362   DNAPK       .
# AAH03551.1         526 T   PECGTKYLL   0.356   CKI         .
# AAH03551.1         526 T   PECGTKYLL   0.346   p38MAPK     .
# AAH03551.1         526 T   PECGTKYLL   0.337   PKA         .
# AAH03551.1         526 T   PECGTKYLL   0.268   PKG         .
# AAH03551.1         526 T   PECGTKYLL   0.243   ATM         .
# AAH03551.1         526 T   PECGTKYLL   0.201   RSK         .
# AAH03551.1         526 T   PECGTKYLL   0.168   PKC         .
# AAH03551.1         526 T   PECGTKYLL   0.166   cdk5        .
# AAH03551.1         526 T   PECGTKYLL   0.090   PKB         .
#
# AAH03551.1         528 Y   CGTKYLLNL   0.600   unsp        YES
# AAH03551.1         528 Y   CGTKYLLNL   0.426   INSR        .
# AAH03551.1         528 Y   CGTKYLLNL   0.339   EGFR        .
# AAH03551.1         528 Y   CGTKYLLNL   0.319   SRC         .
#
# AAH03551.1         538 S   LEPFSGKAL   0.950   unsp        YES
# AAH03551.1         538 S   LEPFSGKAL   0.460   GSK3        .
# AAH03551.1         538 S   LEPFSGKAL   0.437   CaM-II      .
# AAH03551.1         538 S   LEPFSGKAL   0.387   cdc2        .
# AAH03551.1         538 S   LEPFSGKAL   0.379   CKI         .
# AAH03551.1         538 S   LEPFSGKAL   0.365   CKII        .
# AAH03551.1         538 S   LEPFSGKAL   0.345   DNAPK       .
# AAH03551.1         538 S   LEPFSGKAL   0.282   p38MAPK     .
# AAH03551.1         538 S   LEPFSGKAL   0.279   PKC         .
# AAH03551.1         538 S   LEPFSGKAL   0.276   ATM         .
# AAH03551.1         538 S   LEPFSGKAL   0.223   PKG         .
# AAH03551.1         538 S   LEPFSGKAL   0.219   RSK         .
# AAH03551.1         538 S   LEPFSGKAL   0.204   PKA         .
# AAH03551.1         538 S   LEPFSGKAL   0.192   cdk5        .
# AAH03551.1         538 S   LEPFSGKAL   0.079   PKB         .
#
# AAH03551.1         544 S   KALCSWSIC   0.445   CaM-II      .
# AAH03551.1         544 S   KALCSWSIC   0.443   cdc2        .
# AAH03551.1         544 S   KALCSWSIC   0.440   GSK3        .
# AAH03551.1         544 S   KALCSWSIC   0.423   CKII        .
# AAH03551.1         544 S   KALCSWSIC   0.380   CKI         .
# AAH03551.1         544 S   KALCSWSIC   0.374   RSK         .
# AAH03551.1         544 S   KALCSWSIC   0.361   PKA         .
# AAH03551.1         544 S   KALCSWSIC   0.350   DNAPK       .
# AAH03551.1         544 S   KALCSWSIC   0.345   PKC         .
# AAH03551.1         544 S   KALCSWSIC   0.339   p38MAPK     .
# AAH03551.1         544 S   KALCSWSIC   0.315   PKG         .
# AAH03551.1         544 S   KALCSWSIC   0.297   ATM         .
# AAH03551.1         544 S   KALCSWSIC   0.197   cdk5        .
# AAH03551.1         544 S   KALCSWSIC   0.092   PKB         .
# AAH03551.1         544 S   KALCSWSIC   0.006   unsp        .
#
# AAH03551.1         546 S   LCSWSIC--   0.447   CaM-II      .
# AAH03551.1         546 S   LCSWSIC--   0.442   GSK3        .
# AAH03551.1         546 S   LCSWSIC--   0.376   cdc2        .
# AAH03551.1         546 S   LCSWSIC--   0.373   CKII        .
# AAH03551.1         546 S   LCSWSIC--   0.364   CKI         .
# AAH03551.1         546 S   LCSWSIC--   0.350   DNAPK       .
# AAH03551.1         546 S   LCSWSIC--   0.314   RSK         .
# AAH03551.1         546 S   LCSWSIC--   0.310   ATM         .
# AAH03551.1         546 S   LCSWSIC--   0.307   PKA         .
# AAH03551.1         546 S   LCSWSIC--   0.291   PKG         .
# AAH03551.1         546 S   LCSWSIC--   0.290   p38MAPK     .
# AAH03551.1         546 S   LCSWSIC--   0.171   cdk5        .
# AAH03551.1         546 S   LCSWSIC--   0.083   PKC         .
# AAH03551.1         546 S   LCSWSIC--   0.078   PKB         .
# AAH03551.1         546 S   LCSWSIC--   0.006   unsp        .
#
    MAEELVLERCDLELETNGRDHHTADLCREKLVVRRGQPFWLTLHFEGRNY   #    50
    EASVDSLTFSVVTGPAPSQEAGTKARFPLRDAVEEGDWTATVVDQQDCTL   #   100
    SLQLTTPANAPIGLYRLSLEASTGYQGSSFVLGHFILLFNAWCPADAVYL   #   150
    DSEEERQEYVLTQQGFIYQGSAKFIKNIPWNFGQFEDGILDICLILLDVN   #   200
    PKFLKNAGRDCSRRSSPVYVGRVVSGMVNCNDDQGVLLGRWDNNYGDGVS   #   250
```

```
     PMSWIGSVDILRRWKNHGCQRVKYGQCWVFAAVACTVLRCLGIPTRVVTN    #    300
     YNSAHDQNSNLLIEYFRNEFGEIQGDKSEMIWNFHCWVESWMTRPDLQPG    #    350
     YEGWQALDPTPQEKSEGTYCCGPVPVRAIKEGDLSTKYDAPFVFAEVNAD    #    400
     VVDWIQQDDGSVHKSINRSLIVGLKISTKSVGRDEREDITHTYKYPEGSS    #    450
     EEREAFTRANHLNKLAEKEETGMAMRIRVGQSMNMGSDFDVFAHITNNTA    #    500
     EEYVCRLLLCARTVSYNGILGPECGTKYLLNLNLEPFSGKALCSWSIC      #    550
%1   ......................T..................T........    #     50
%1   .....S.T....T....S....T.................T.........    #    100
%1   S....T...........S...ST.....S...................Y.    #    150
%1   .S......Y..T........S.............................    #    200
%1   ...........S..SS..Y.....S...................Y....S    #    250
%1   ..S................................T........T...T.    #    300
%1   ..S........................S......................    #    350
%1   .........T....S...Y...............ST..............    #    400
%1   ..........S...S...S.......S..S...........T......SS    #    450
%1   .............................................T....    #    500
%1   ..Y...........S............Y.........S..........
```

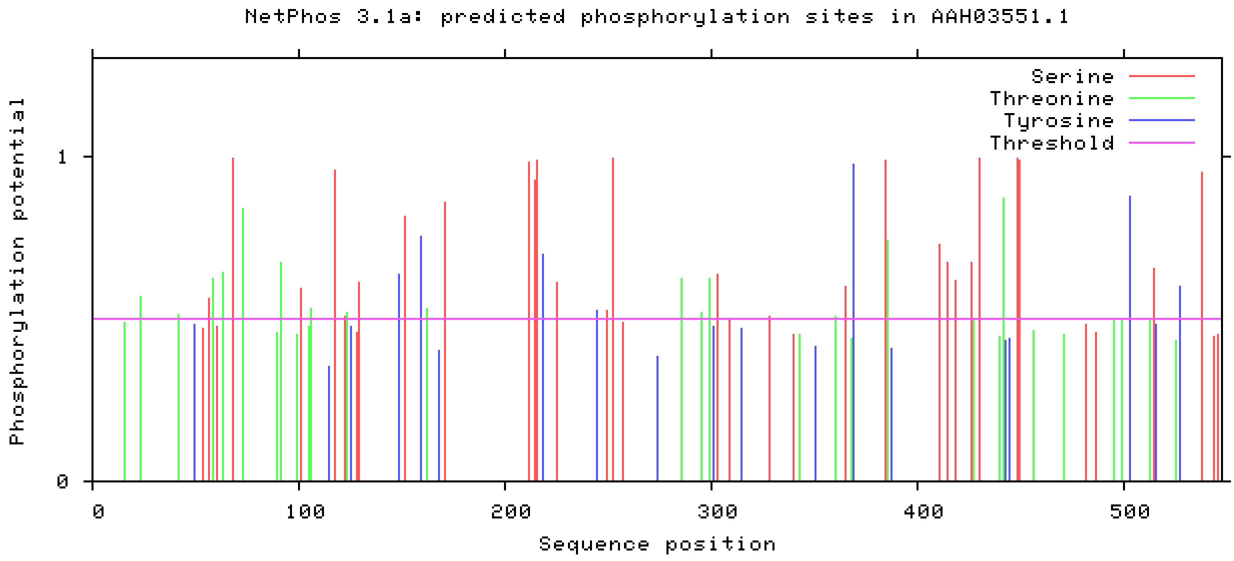


**Explain** the output. Go **back**.
