# Supplementary material for: Nuclear Transglutaminase 2 interacts with topoisomerase II⍺ to promote DNA damage repair in lung cancer cells
Source: J Exp Clin Cancer Res. 2021 Jul 5;40:224. doi: 10.1186/s13046-021-02009-2 (PMC8258933; doi:10.1186/s13046-021-02009-2)
Supplement: Supplementary file 14 — Additional file 14. [file 13046_2021_2009_MOESM14_ESM.docx]

**Table S6 TG2 expression and clinical parameters.**

| Parameters | TG2 expression | | Total  (n=80) | P value |
| --- | --- | --- | --- | --- |
|  | Low TG2 | High TG2 |  |  |
| Age (yr) |  |  |  |  |
| <60 | 20 | 21 | 41 | 1 |
| >60 | 20 | 19 | 39 |  |
| Sex |  |  |  |  |
| Male | 29 | 25 | 54 | 0.4739 |
| Female | 11 | 15 | 26 |  |
| Invasion |  |  |  |  |
| Yes | 8 | 9 | 17 | 1 |
| No | 32 | 31 | 63 |  |
| Histology |  |  |  |  |
| Adenocarcinoma | 21 | 28 | 47 | 0.1685 |
| No-Ade. | 19 | 12 | 31 |  |
| Adjuvant chemotherapy |  |  |  |  |
| Yes | 12 | 20 | 32 | 0.1101 |
| No | 28 | 20 | 48 |  |
| Smoking history |  |  |  |  |
| Yes | 18 | 11 | 29 | 0.1629 |
| No | 22 | 29 | 51 |  |
